# Transcriptome-Wide Prediction of miRNA Targets in Human and Mouse Using FASTH

**DOI:** 10.1371/journal.pone.0005745

**Published:** 2009-05-29

**Authors:** Chikako Ragan, Nicole Cloonan, Sean M. Grimmond, Michael Zuker, Mark A. Ragan

**Affiliations:** 1 The University of Queensland, Institute for Molecular Bioscience, and ARC Centre of Excellence in Bioinformatics, Brisbane, Australia; 2 Rensselaer Polytechnic Institute, Troy, New York, United States of America; Lawrence Berkeley National Laboratory, United States of America

## Abstract

Transcriptional regulation by microRNAs (miRNAs) involves complementary base-pairing at target sites on mRNAs, yielding complex secondary structures. Here we introduce an efficient computational approach and software (FASTH) for genome-scale prediction of miRNA target sites based on minimizing the free energy of duplex structure. We apply our approach to identify miRNA target sites in the human and mouse transcriptomes. Our results show that short sequence motifs in the 5′ end of miRNAs frequently match mRNAs perfectly, not only at validated target sites but additionally at many other, energetically favourable sites. High-quality matching regions are abundant and occur at similar frequencies in all mRNA regions, not only the 3′UTR. About one-third of potential miRNA target sites are reassigned to different mRNA regions, or gained or lost altogether, among different transcript isoforms from the same gene. Many potential miRNA target sites predicted in human are not found in mouse, and *vice-versa*, but among those that do occur in orthologous human and mouse mRNAs most are situated in corresponding mRNA regions, *i.e.* these sites are themselves orthologous. Using a luciferase assay in HEK293 cells, we validate four of six predicted miRNA-mRNA interactions, with the mRNA level reduced by an average of 73%. We demonstrate that a thermodynamically based computational approach to prediction of miRNA binding sites on mRNAs can be scaled to analyse complete mammalian transcriptome datasets. These results confirm and extend the scope of miRNA-mediated species- and transcript-specific regulation in different cell types, tissues and developmental conditions.

## Introduction

miRNAs are endogenous short (∼22 nt) RNAs that exert regulatory control of many cellular processes, negatively regulating specific mRNAs *via* complementary base-pairing at a target site. miRNAs of plants bind the targeted mRNA with high complementarity and thereby mark it for degradation, whereas animal miRNAs more typically bind with sub-optimal complementarity and inhibit or diminish productive translation [1].

Target sites for complementary base-pairing by miRNAs can be inferred using computational methods based on empirically determined features of how known miRNAs bind *in vivo* to validated target sites. For example, perfect Watson-Crick (WC) matching over a 6- or 7-nt “seed” region at the 5′ end of the miRNA [2]–[4] is very important for target recognition and can by itself repress translation [4]. Base-pairing elsewhere in the miRNA, including at a so-called T1A site extending the seed region [3] and in the 3′ region [3], [4], has also been variously proposed to contribute to target binding. Although not every validated miRNA-mRNA pair exhibits a short region of perfect WC complementarity [5]–[9] and computational methods based entirely on such seeds will fail to find every likely target, many experimentally validated target sites exhibit perfect WC matches in the seed region.

Several distinct algorithmic approaches are utilized to predict miRNA targets. The most widely used approach is a sequence-based search, in which a local sequence alignment tool is used to find target sites with near-perfect WC complementarity to the query miRNA [10]–[14]. In this approach, the existence of this seed region is often assumed. Because miRNA target sites are short and may exhibit only limited complementarity outside the seed region, additional criteria based on abstractions of *in vivo* processes are then employed to improve the efficiency of the extension step; these include requiring that targets be conserved across homologous mRNAs from different species, or requiring multiple matches by one or more miRNAs. Target sites predicted using this approach may subsequently be ranked according to hybridization energy score [12], [13], but the actual search is alignment-based.

In a second approach, machine learning [15] and pattern discovery [9] have been applied to capture features of mRNAs that are known or suspected to bind mRNAs. This approach builds on numerous other applications in genomics and bioinformatics, but has two important limitations. As relatively few miRNA target sites have been functionally validated, such predictors cannot yet be precise, and discovery must continually be re-run on the entire database as new miRNAs are discovered and new binding sites validated. Further, biologically relevant features that contribute to the predictions may not be captured or, if captured, may remain unknown to the user.

Both of the above approaches are complicated by regions of unpaired nucleotides (loops and bulges) known to be present in many miRNA-target interactions [16]. To accommodate these regions of imperfect pairing in duplexes between the 3′ end of a miRNA and its mRNA target site, sophisticated sequence-based methods may search for near-perfect WC complementarity in the seed region (miRNA positions 2–7, 2–8 or 1–7) [11], [14] then extend the search *e.g.* to the 3′ end of the miRNA, rather than attempting to align the entire miRNA in a single operation.

A third approach [16], [17] is based on the perspective that it is the thermodynamic stability of the RNA-RNA duplex, not its abstraction as a string-match or model, which allows miRNAs to bind targets *in vivo*. Potential target sites are identified as those at which the free energy of hybridization is minimized; complex structures involving loops and bulges can readily be accommodated in this calculation. We adopt this third approach in the work reported here. We introduce the FASTH (FAST Hybridization) program and, as a first stage, use it to predict potential miRNA-mRNA interaction sites. The name was chosen to be similar to FASTA [18] because both programs employ similar search strategies and both allow bulges (gaps) in the local alignment. With FASTH, however, we follow a purely biophysical path, searching for binding targets taking into account (a) RNA base-pair and base-pair-stacking free energies [19] in perfect helices, (b) the unfavorable contributions from interior loops and bulges, (c) energy contributions from single-stranded bases and from mismatched pairs adjacent to a base pair, and (d) the initiation energy required to bring the two molecules together and initiate duplex formation. We assume that the population of duplexes actually formed between a miRNA and its target region on an individual mRNA is best represented by the duplex with the minimum free energy possible, even if this implies the presence of non-WC base pairs, loops, bulges and/or mismatches.

In many cases, RNA-RNA hybridization results in the formation of a population of duplexes, one (or a few) with minimum free energy and many others with increasingly suboptimal energies. miRNA-mRNA duplexes with slightly suboptimal free energies probably occur *in vivo*, perhaps more transiently or at lower frequencies, and might be biologically relevant. Finding these suboptimal structures is computationally expensive, and previous work [16], [17] employed dynamic programming to search the target database. FASTH uses a fast heuristic (*i.e.* not dynamic programming) for the database search, with search time scaling sub-linearly with database size; it can return a user-specified number (hundreds or thousands) of suboptimal sites, and process multiple databases with rigorous minimum free-energy calculations, in a single search.

In the second stage of our approach, we then select those FASTH results that satisfy empirically derived rules unrelated to energy minimization *per se*, including perfect WC complementarity to the target at the 5′ end of miRNAs, minimum numbers of WC base pairs at the 3′ end, and/or specified free energy score thresholds. As we demonstrate, further criteria can be imposed as well, capturing features *e.g.* of the specific mRNA region bound (5′UTR, CDS, or 3′ UTR), presence of orthologs in other transcriptomes, and/or levels of sequence conservation. Energy-based prediction has recently been extended to include a penalty term that reflects the free-energy cost associated with disruption of pre-existing secondary structure at potential target sites on the mRNA [20]–[22]; such a term could, if desired, be easily incorporated into our approach.

With few exceptions, miRNA target-prediction methods have until now been applied to EST data, focusing on only the 3′UTR. Here we apply our approach to the complete human and mouse transcriptomes as represented in RefSeq. We parameterized the second-stage requirements within biologically reasonable ranges, and observe the effects on number of predicted sites and on statistical significance. We demonstrate that short strings of matching nucleotides (usually 6 or 7 in length) appear more frequently in the 5′ end than in the 3′ end of human miRNAs. We show that many energetically favourable binding sites with perfect seed matches, *i.e.* potential miRNA targets, occur in all mRNA regions, and that a substantial number of these sites are reassigned between mRNA regions depending on the specific transcript isoform. This opens the possibility that, at least in human, alternative transcripts of many genomic regions may be differentially regulated by miRNAs. Selected results were thereafter taken forward into laboratory-based validation. Although the results reported below focus on results with 313 human miRNAs, very similar results were also obtained for 233 mouse miRNAs and are presented in the Supplementary Material S1.

## Results

### Predicted target sites for an individual miRNA

**Figure 1:**
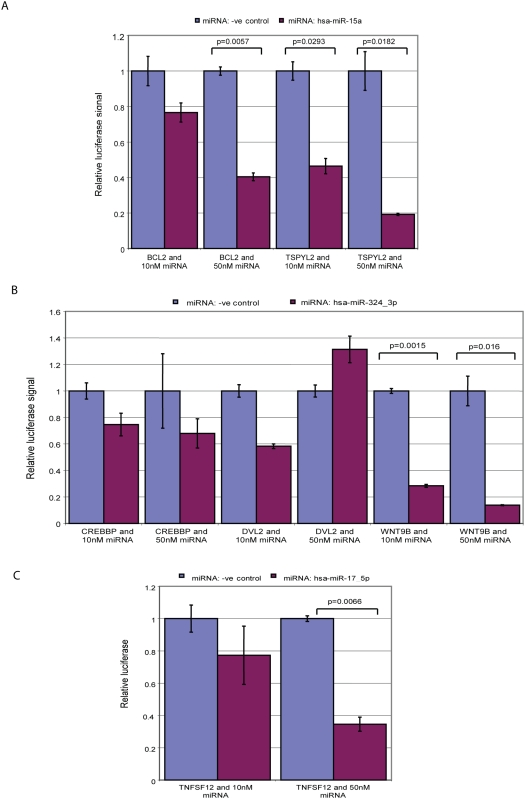
Number of predicted miRNA target sites for selected miRNAs in human RefSeq mRNAs distributed through a range of free energy scores. Numbers of predicted target sites per miRNA and its control sequences for (A) miR-1 and its controls with WC nt 2–8; if miR-1 hybridized with perfect WC complementarity this would yield −30.8 kcal/mol (see Methods); (B) let-7a and imposing only the requirement of WC base pairs within nucleotide positions 2–8; let-7a perfect WC complementarity would yield −33.2 kcal/mol; (C) miR-17-5p and its controls with WC nt 2–8; perfect WC complementarity would yield −44.5 kcal/mol; (D) miR-324-3p and its controls with WC nt 2–8; perfect WC complementarity would yield −52.8 kcal/mol; and (E) miR-129 and its controls with WC nt 2–8; perfect WC complementarity would yield −41.4 kcal/mol. Blue bars show distributions for native miRNAs, red bars for mononucleotide-shuffled controls (MS), and green bars for first-order Markov controls (FOM) (see Supplementary Text).

For each miRNA it is always the case that our approach predicts a set of targets, distributed through a range of free energy scores. To assess their quality, we compare them statistically against the targets predicted, under the same criteria, for a set of control miRNAs (Figure 1A–E). To this end, for each miRNA we employ two sets of controls (mononucleotide shuffled, or MS, and first-order Markov, or FOM) derived to reflect different assumptions, as described in the Supplementary Text S1; each control miRNA finds a set of targets, likewise distributed over a range of free energies. The targets predicted for the real miRNA may be distributed through better, similar, or worse free energies than are the targets predicted for its controls. For example, targets predicted for let-7a are shifted toward better free energies than are those predicted for both sets of controls (Figure 1B), whereas targets predicted for miR-17-5p and its controls show similar energy distributions (Figure 1C). In some cases the true miRNA found more target sites than did, on average, the corresponding controls (miR-324-3p, Figure 1D), whereas in other cases each control, on average, found more targets than did the miRNA (miR-129, Figure 1E).

Over all these miRNAs, the sets of predicted targets also vary by free energy score. The targets predicted for some miRNAs (and for the corresponding controls) show high (poor) energies, with miR-1 among the most extreme in this regard (Figure 1A), whereas better energies are predicted for others. Panels B, C and D of Figure 1 show the increasingly better energy-score ranges for predicted targets of let-7a, miR-17-5p and miR-324-3p. The diversity of these examples with respect to the range (kcal/mol) and shape of the distribution of energy scores, and number of target sites predicted with real *versus* control sequences, reflects an estimate of the diversity of molecular interactions over the transcriptome. We return below to the distribution of predicted targets over all human miRNAs.

For binding sites experimentally validated in human [23], the actual hybridisation energies returned by FASTH are similar to those calculated by Tafer and Hofacker using RNAduplex and RNAplex [17], but are quite different from those computed by Rehmsmeier *et al*. using RNAhybrid [16].

### Predicted target sites over all human miRNAs

**Figure 2:**
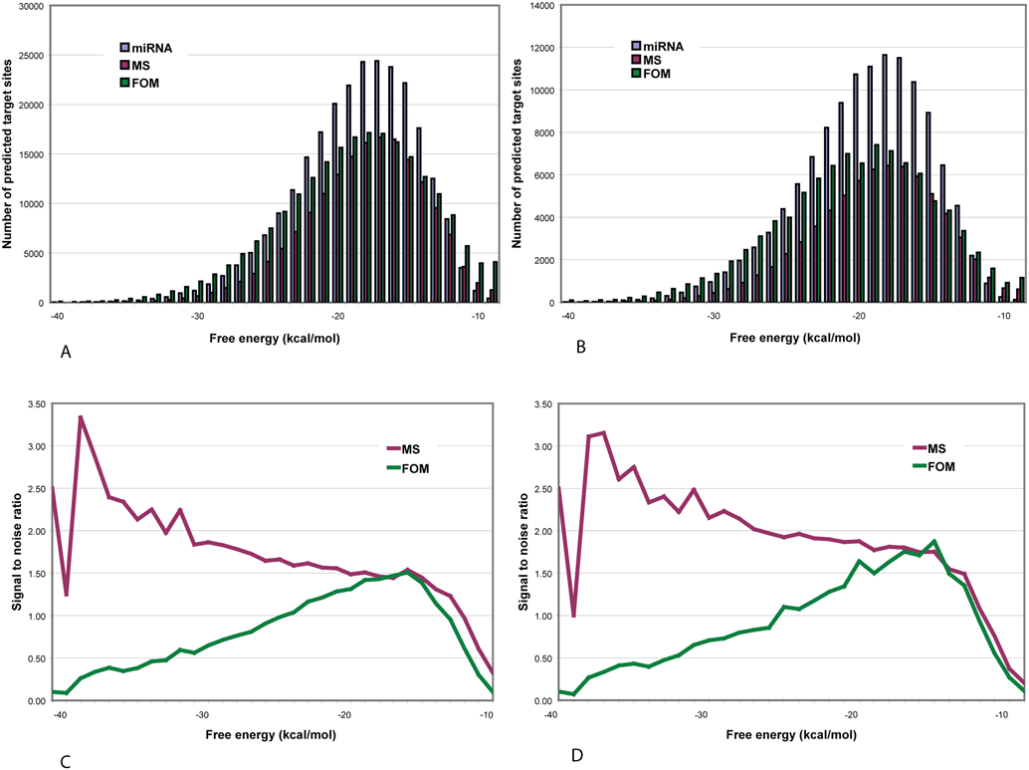
Number of predicted miRNA target sites and signal-to-noise ratio in human RefSeq mRNAs distributed through a range of free energy scores. Numbers of predicted target sites (A) imposing only the requirement of WC base pairs within nucleotide positions 2–8; (B) requiring WC base pairs at positions 2–8, plus <6 mismatches and GU pairs at position 15 and beyond (see text). For (A) and (B), blue bars show distributions for native miRNAs, red bars for mononucleotide shuffled (MS) controls, and green bars for first-order Markov (FOM) controls (see Methods). Signal-to-noise ratio based on MS controls (red bars) and FOM controls (green bars), (C) requiring WC base pairs at nucleotide positions 2–8 alone, and (D) requiring WC base pairs at positions 2–8, plus <6 mismatches and GU pairs at position 15 and beyond.

As we have seen, the set of predicted targets for an individual miRNA can show diversity in number (frequency) and free energy profile. In Figure 2A–D this case-to-case variation is aggregated over all sites predicted for the 313 human miRNAs considered in this study; panels A and C show results with minimal second-stage filtering (requiring only perfect WC complementarity at nucleotide positions 2–8), while panels B and D reflect the imposition of an additional criterion (that there be <6 mismatches and GU pairs at positions ≥15).

In either case, at the lower free energies in aggregate these miRNAs find more predicted target sites than do control sequences of the same length and nucleotide composition (Figure 2A,B). At free energies better than about −15 kcal/mol the signal-to-noise ratio (S∶N, see Methods) remains above 1.5, but this ratio diminishes rapidly at higher free energies (Figure 2C,D). At predicted free energies better than about −12 kcal/mol, on average these miRNAs find more target sites than do the MS controls. In comparison with the FOM controls, however, the miRNAs find more target sites only at energies between about −13 and −23 kcal/mol (Figure 2A). The greatest fold difference reaches 3.33 against the MS controls (albeit for small numbers of predictions, at −38 kcal/mol) but only 1.51 against the FOM set (Figure 2C). Similar behaviour is seen for the subset of predictions that survive more-rigorous second-stage filtering (Figure 2B,D). Thus at least for miRNA-mRNA binding sites with perfect WC complementarity in a 7-nt seed region, dinucleotide frequency bias is a main contributor to stability of the predicted duplex.

The S∶N ratio diminishes rapidly at free energies greater (*i.e.* worse) than about −15 kcal/mol regardless of whether MS or FOM controls are used, falling below 1.00 at energies above about −14 and −11 kcal/mol when calculated against the FOM and MS control sets respectively (Figure 2C). Very similar results are seen after second-stage filtering (Figure 2D), with S∶N falling below 1.00 at energies above about −12 kcal/mol. These results indicate that an energy-based approach will be increasingly unproductive as the free energy of duplex formation becomes progressively weaker beyond a threshold determined, in part, by details of the second-stage filtering criteria; conversely, ignoring such weak duplexes should reduce the frequency of false-positive predictions. Restricting allowable free energies by introduction of a higher energy threshold improves the overall S∶N ratio by around 10–20% for this set of miRNAs (Supplementary Table S1, Supplementary Table S2, and Methods).

At energies more-favorable than about −17 kcal/mol, the S∶N ratio either decreases or increases depending on whether the FOM or MS control set is used (Figure 2C–D). As argued above, we suspect that use of FOM underestimates S∶N, while use of MS may overestimate S∶N.

### Free energies of predicted target duplexes are correlated with the potential binding energy of each miRNA

**Figure 3:**
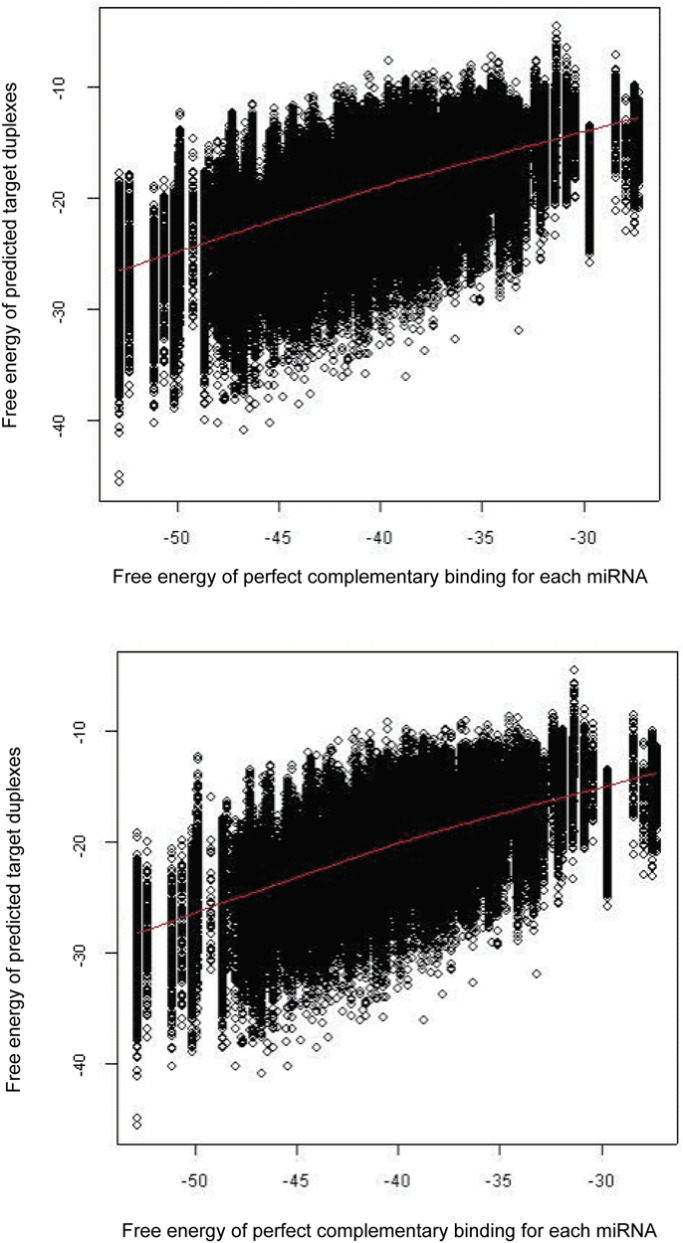
Calculated free energy of duplex formation at the miRNA target sites predicted by our approach, *versus* the minimum possible free energy for that miRNA binding with perfect WC base-pair complementarity to a (theoretical) target site. Free energy scores of predicted target sites (y-axis) are plotted against the free energy score of each miRNA, where each of 313 human miRNAs binds to a target site with perfect WC base pair complementary, imposing the requirement(s) of (A) WC base pairs within nucleotide positions 2–8; and (B) WC base pairs within positions 2–8, plus <6 mismatches-and-GU pairs at position 15 and beyond (see text for details). The red line is the non-parametric local fitted line.

Figure 3 shows, over all these 313 human miRNAs, the free energies of our predicted target duplexes plotted against the potential binding energy if each miRNA were bound to an ideal target site with perfect WC complementarity. These miRNAs bind at their potential target sites within a wide band of energies, bounded on one side by the energy score at perfect WC base pair complementarity and on the other by the score associated with the minimal extent of interaction we consider (*e.g.* only the seed regions). The actual predicted free energies are broadly correlated with the best possible energies but with a wide range of sub-optimality.

Experimentally validated targets are not restricted to mRNAs with the energetically most-favorable target sites. For example, five experimentally validated targets of miR-1 (Figure 1A) have energy scores between −10.9 and −14.5 kcal/mol (for details see Table 1). Thus a high (weak) energy score does not by itself disqualify a predicted site. We are not hypothesizing that true miRNAs are those that bind most stably, only that stability (unlike its abstraction as a string) is important in prediction of potential target sites. Indeed a degree of energetic sub-optimality may be necessary, where single miRNA has to bind many different target sites in different mRNAs.

**Table 1:** miRNA targets experimentally validated previously in human from TarBase [23], and our predictions.

Num	Validated miR	Gene	Reference	Prediction	Predicted miR	ID of predicted mRNAs	FE	UTR/CDS
1	miR-189	SLITRK1	37	* **	hsa-miR-189	NM_052910	−17.7	3′
2	miR-1	GJA1	38		hsa-miR-1	NM_000165	−14.3	3′
3	miR-206	GJA1	38	**	hsa-miR-206	NM_000165	−18.4	3′
4	miR-133	PTBP2	39		hsa-miR-133a	NM_021190	−18.9	3′
5	miR-1	HDAC4	40		hsa-miR-1	NM_006037	−14.1	3′
5	miR-1	HDAC4	40		hsa-miR-1	NM_006037	−13.9	3′
6	miR-133	SRF	40	not predicted				
7	miR-15a	BCL2	28		hsa-miR-15a	NM_000633	−19.0	3′
8	miR-16	BCL2	28		hsa-miR-16	NM_000633	−18.9	3′
9	miR-223	NFIA	41	*	hsa-miR-223	NM_005595	−18.6	3′
10	miR-221	KIT	42	not predicted				
11	miR-222	KIT	42	not predicted				
12	miR-10a	HOXA1	43	* **	hsa-miR-10a	NM_005522	−13.9	3′
13	miR-130	MAFB	43	not predicted				
14	let-7	KRAS	44	not predicted				
15	let-7	NRAS	44	not predicted				
16	miR-192	SIP1	45	not predicted				
17	let-7b	LIN28	46	*	hsa-let-7b	NM_024674	−22.0	3′
18	let-7e	SMC1L1	46	*	hsa-let-7e	NM_006306	−21.7	3′
19	miR-141	CLOCK	46	*	hsa-miR-141	NM_004898	−15.8	3′
20	miR-15	DMTF1	46		hsa-miR-15a	NM_021145	−20.9	3′
21	miR-16	CGI-38	46		hsa-miR-16	NM_015964	−20.8	3′
22	miR-199b	LAMC2	46		hsa-miR-199b	NM_005562	−21.4	3′
23	miR-24	MARK14	46		hsa-miR-24	NM_139012	−27.1	3′
24	miR-24	MARK14	46		hsa-miR-24	NM_139014	−27.1	3′
24	let-7b	MTPN	14	**	hsa-let-7b	NM_145808	−15.6	3′
25	miR-124	MAPK14	14	**	hsa-miR-124a	NM_001315	−15.8	3′
26	miR-124	MTPN	14	not predicted				
27	miR-375	ADIPOR2	14	not predicted				
28	miR-375	C1QBP	14	not predicted				
29	miR-375	JAK2	14	not predicted				
30	miR-375	USP1	14	not predicted				
31	miR-1	BDNF	11	*	hsa-miR-1	NM_001709	−14.5	3′
31	miR-1	BDNF	11	*	hsa-miR-1	NM_170732	−14.5	3′
31	miR-1	BDNF	11	*	hsa-miR-1	NM_170735	−14.5	3′
31	miR-1	BDNF	11	*	hsa-miR-1	NM_170734	−14.5	3′
31	miR-1	BDNF	11	*	hsa-miR-1	NM_170733	−14.5	3′
31	miR-1	BDNF	11	*	hsa-miR-1	NM_170731	−14.5	3′
32	miR-1	G6PD	11	**	hsa-miR-1	NM_000402	−10.8	3′
33	miR-101	MYCN	11	**	hsa-miR-101	NM_005378	−13.9	3′
33	miR-101	MYCN	11	**	hsa-miR-101	NM_005378	−13.5	3′
34	miR-19a	PTEN	11	* predicted in CDS	hsa-miR-19a	NM_000314	−15.6	CDS
35	miR-23	POU4F2	11	**	hsa-miR-19a	NM_004575	−13.3	3′
36	miR-23a	CXCL12	11	* predicted in CDS	hsa-miR-23a	NM_000609	−12.5	CDS
37	miR-26a	SMAD1	11	**	hsa-miR-26a	NM_005900	−13.6	3′
38	miR-34	DLL1	11		hsa-miR-34a	NM_005618	−23.2	3′
39	miR-34	DLL1	11		hsa-miR-34a	NM_005618	−15.5	3′
39	miR-34	NOTCH1	11	*	hsa-miR-34a	NM_017617	−21.8	3′
40	miR-155	AGTR1	47	not predicted				
41	miR-21	PTEN	48	not predicted				
42	miR-181	HOXA11	49		hsa-miR-181b	NM_005522	−18.6	3′
42	miR-181	HOXA11	49		hsa-miR-181b	NM_153620	−18.6	3′
42	miR-181	HOXA11	49		hsa-miR-181d	NM_005522	−17.8	3′
42	miR-181	HOXA11	49		hsa-miR-181d	NM_153620	−17.8	3′
43	miR-17-5p	E2F1	50	not predicted				
44	miR-20	E2F1	50	not predicted				
45	miR-375	MTPN	51	not predicted				
46	miR-206	FSTL1	52	not predicted				
47	miR-206	UTRN	52	not predicted				
48	miR-127	BCL6	53	not predicted				
49	miR-125a	ERBB2	54	* **	hsa-miR-125a	NM_001005862	−15.5	3′
50	miR-125a	ERBB3	54	* **	hsa-miR-125a	NM_004448	−15.5	3′
51	miR-27b	CYP1B1	55		hsa-miR-27b	NM_000104	−26.0	3′
52	miR-140	HDAC4	56	not predicted				
53	miR-106a	RB1	57	not predicted				
54	miR-20a	TGFBR2	57	not predicted				
55	miR-26a	PLAG1	57	**	hsa-miR-26a	NM_002655	−14.7	3′
56	miR-372	LAST2	58	**	hsa-miR-372	NM_014572	−13.1	3′
57	miR-373	LAST2	58	**	hsa-miR-373	NM_014572	−15.7	3′
58	miR-34a	E2F3	59	*	hsa-miR-34a	NM_001949	−17.6	3′
59	miR-133	ERG	60	predicted in CDS	hsa-miR-133a	NM_004449	−18.8	CDS
59	miR-133	ERG	60		hsa-miR-133a	NM_182918	−18.8	CDS
60	miR-196	HOXA7	7	not predicted				
61	miR-196	HOXC8	7		hsa-miR-196a	NM_022658	−15.8	3′
62	miR-196	HOXD8	7	** predicted in CDS	hsa-miR-196a	NM_019558	−13.5	CDS
63	miR-1	HAND2	61	* **	hsa-miR-1	NM_021973	−10.9	3′
64	miR-1	TMSB4X	61		hsa-miR-1	NM_021109	−16.9	3′
65	miR-21	TPM1	62	not predicted				

* not predicted with the second-stage parameter “under 6 mismatches-and-GU-pairs for nt ≥15”.

** not predicted with 40% FE threshold.

### For known miRNAs, occurrence of a 6–7 nucleotide region of perfect WC base-pairing with mRNAs is biased toward the 5′ end of miRNAs

The minimum sequence complementarity and thermodynamics of hybridization required for a region of mRNA to serve as a miRNA target site *in vitro* are not fully understood. However, many computational and experimental studies have addressed how miRNAs recognize and pair with their mRNA target sites [2], [4], [10], [12]–[14], [24]. One of the most important determinants of this interaction is the so-called seed region, a stretch of 6 or 7 contiguous nucleotides at or near the 5′ end of a miRNA involved in perfect WC base-pairing with the mRNA [2].

**Figure 4:**
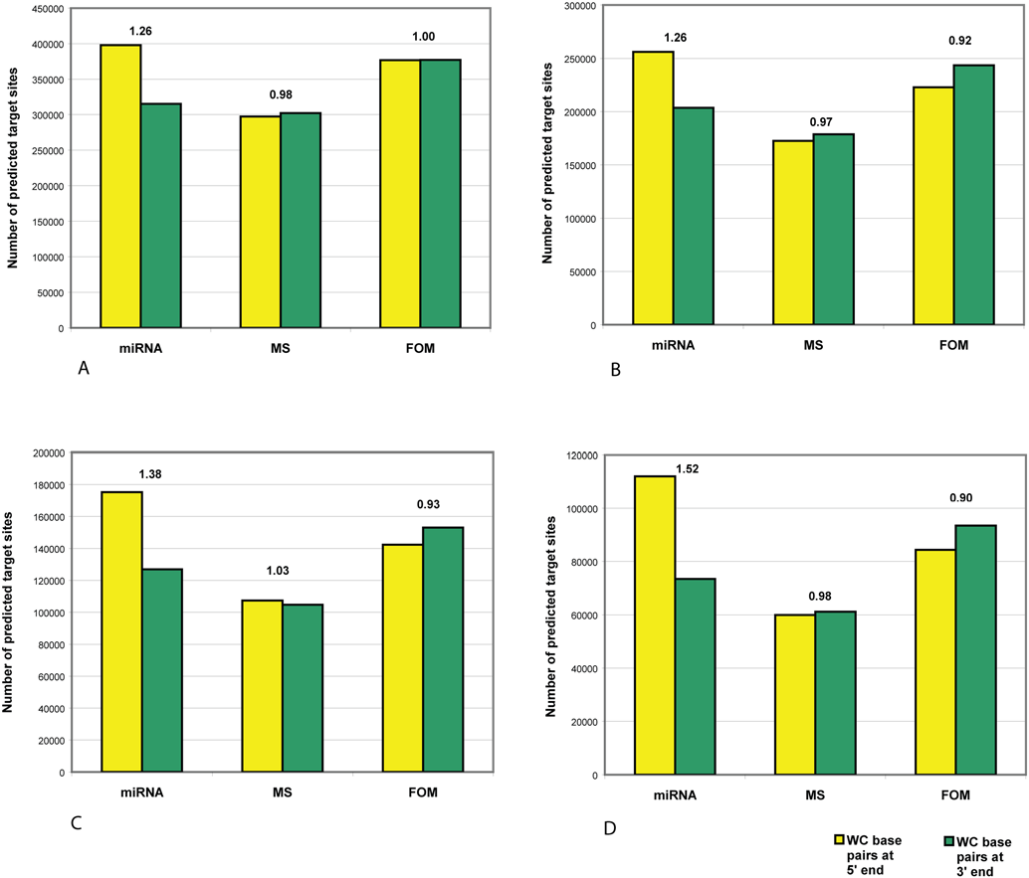
Number of predicted miRNA target sites with perfect WC base-pair complementarity at the 5′ end and at the 3′ end of miRNAs in human RefSeq mRNAs. Numbers of predicted target sites and ratios (the number of predicted target sites with perfect WC base pairs at 5′ end divided by the number of predicted target sites with perfect WC base pairs at 3′ end) for known miRNAs, and mononucleotide shuffled (MS) and first-order Markov (FOM) controls (see Methods), with perfect WC base pair complementary at the 5′ end (yellow bars) and 3′ end (green bars) of miRNAs, imposing the requirement(s) of (A) WC base pairs within nucleotide positions 2–7; (B) WC base-pairs within positions 2–8; (C) WC base pairs within positions 2–7. plus <6 mismatches-and-GU pairs at position 15 and beyond, and 40% free energy threshold; and (D) WC base pairs within positions 2–8, plus <6 mismatches and GU pairs at position 15 and beyond, and 40% free energy threshold (see text for details). ‘WC base pairs at 5′ end’ indicates the number of target sites when positions are counted from the 5′ end of the miRNAs, and ‘WC base pairs at 3′ end’ indicates these values when positions are counted from the 3′ end of the miRNAs (*i.e.* enforcing perfect WC base pairs at the 3′ end of miRNAs).

We examined whether our predicted targets possess such a 6–7 nt region of perfect WC complementarity, and if so, whether it is preferentially complementary to the 5′ end or to the 3′ end of the corresponding miRNA. To compare preferences to the two ends, we made perfect WC complementarity the second-stage filtering criterion, and sorted the results into one set of potential targets with perfect complementarity to the 5′ ends of miRNAs, and another set with perfect complementarity to the 3′ ends. As FASTH identifies targets based only on free energy of hybridization, without information about or bias toward any particular region of the miRNA, our null hypothesis is that all miRNA regions (including 3′ and 5′ ends) should show equal numbers of perfectly complementary target sites. We then did the same using randomized miRNA sequences as controls (see Methods). We carried out these experimental and control procedures using four sets of conditions that represent 6-nt and 7-nt regions of perfect WC matching, with and without conditions on base-pairing at the 3′ end of miRNA and on binding-energy threshold (see legend to Figure 4 for details).

Figure 4 shows that predicted target sites complementary to the 5′ end of the 313 human miRNAs are much more numerous than those complementary to the 3′ end, whereas little or no such difference is observed with the corresponding MS or FOM controls. For these miRNAs, the 5′ end is favored by a ratio of 1.26–1.52 (depending on second-stage filtering conditions) compared with 0.97–1.03 for the MS and 0.90–1.00 for the FOM controls (Figure 4A–D).

**Figure 5:**
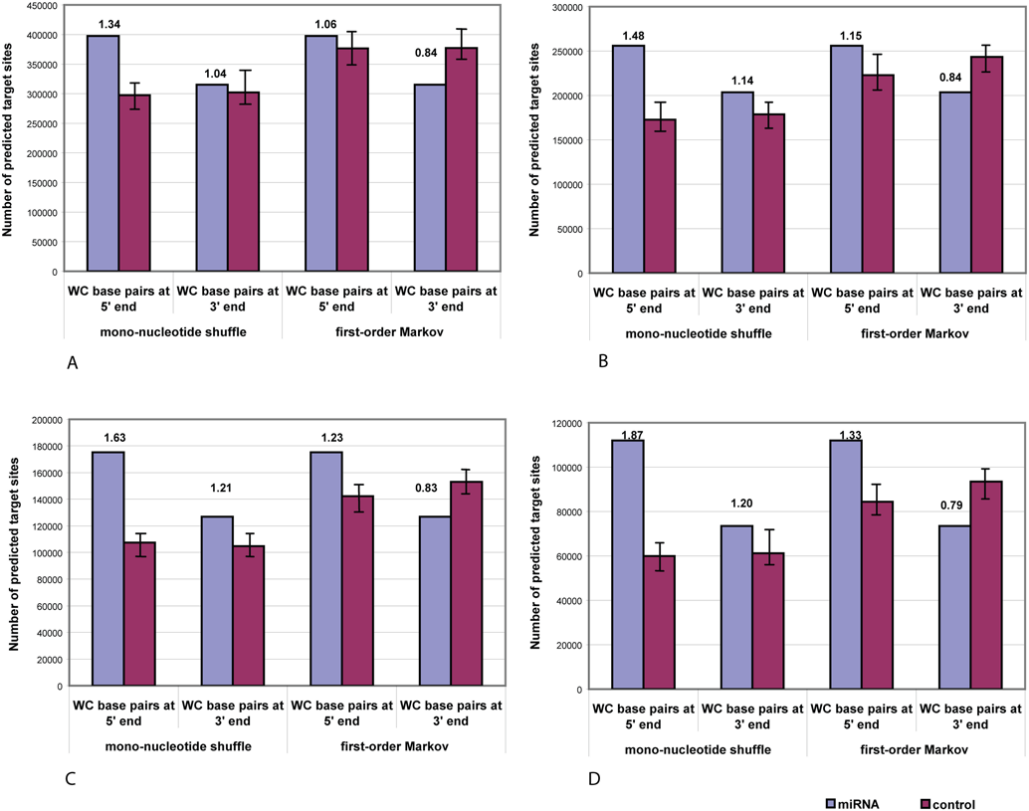
Number of predicted miRNA target sites with perfect WC base-pair complementarity at the 5′ end and at the 3′ end of miRNAs in human RefSeq mRNAs, and their signal-to-noise ratios. Numbers of predicted target sites and signal-to-noise ratios for known miRNAs (blue bars) and mononucleotide shuffled and first-order Markov controls (red bars), with perfect WC base pair complementarity at the 5′ end (yellow bars) and 3′ end (green bars) of miRNAs, imposing the requirement(s) of (A) WC base pairs within nucleotide positions 2–7; (B) WC base pairs within positions 2–8; (C) WC base pairs within positions 2–7, plus <6 mismatches-and-GU pairs at position 15 and beyond, and 40% free energy threshold; and (D) WC base pairs within positions 2–8, plus <6 mismatches-and-GU pairs at position 15 and beyond, and 40% free energy threshold (see text for details). ‘WC base pairs at 5′ end’ indicates the number of target sites when positions are counted from the 5′ end of the miRNAs, and ‘WC base pairs at 3′ end’ indicates these values when positions are counted from the 3′ end of the miRNAs (*i.e.* enforcing perfect WC base pairs at the 3′ end of miRNAs).

Figure 5 presents a second perspective on these results. The number of target sites predicted using known miRNAs, divided by the number predicted using the randomized controls, can be considered as a signal-to-noise ratio. In S∶N ratio as well as in number of predicted targets in mRNAs, we observe a clear preference for perfect WC base pairs involving the 5′ end of miRNAs (Figure 5A–D). The number of high-quality potential binding sites with a 6–7 nt region of perfect complementarity at their 5′ end (*i.e.* a seed region) is substantially (1.34–1.87 times) greater than expected under the null model reflected in the MS control set, and slightly (1.06–1.33 times) greater than expected under the null model reflected by FOM. By contrast, the corresponding ratios for complementarity at the 3′ end are 1.04–1.20 and 0.79–0.84 for the MS and FOM control sets respectively. The ratio of these ratios (1.28–1.55 with MS, 1.26–1.68 with FOM) describes the *selectivity*, here favoring the 5′ end, at each filtering condition. Thus motifs with perfect WC complementarity to a 6–7 nt region at the 5′ end of known miRNAs occur preferentially in mRNAs, relative to motifs complementary to the 3′ end.

For the simplest (although not necessarily biologically meaningful) case considered here, *i.e.* perfect WC base-pairing of the six nucleotides constituting positions 2–7 from either the 5′ or the 3′ end of the (real or randomized) sequences, similar numbers of target sites are predicted at each end of the MS controls and at the 3′ end of real miRNAs (Figure 5A). When positions 2–8 are considered instead (Figure 5B), only predicted sites at the two ends of the MS controls remain similar in number; and as second-stage filtering is made more-stringent by imposing mismatch and free energy thresholds (Figure 5C–D) the predicted target-site numbers, S∶N ratios and 5′-over-3′ selectivity deviate farther. For the conditions reported in Figure 5A–D, (1) more-stringent filtering always reduces the number of predicted target sites; (2) except for 3′ ends under FOM, more-stringent filtering increases the S∶N ratio; (3) more-stringent filtering tends to increase 5′-over-3′ end selectivity; and (4) the FOM controls find more predicted targets than do the corresponding real miRNAs at 3′ ends of mRNA, resulting in S∶N<1.00. We explore some of these parameter conditions in further detail below.

Exact matching in seed regions does not, of course, tell the whole story. A high-quality match may not only involve, but also extend, a seed region. It may also be the case that some sites with perfect WC complementarity in the seed region show poor overall free energy scores and consequently are not identified, by our approach, as potential targets. As demonstrated previously [3], mismatches (including GU pairs) in the seed region may be compensated by optimal complementarity elsewhere. In agreement with many previous studies, though, we are able to use the recognition of a seed region in the 5′ end of miRNAs to identity many energetically favorable candidate mRNA target sites.

### Parameters of binding at the 3′ end of miRNAs affect the number and S∶N ratios of predicted target sites

**Figure 6:**
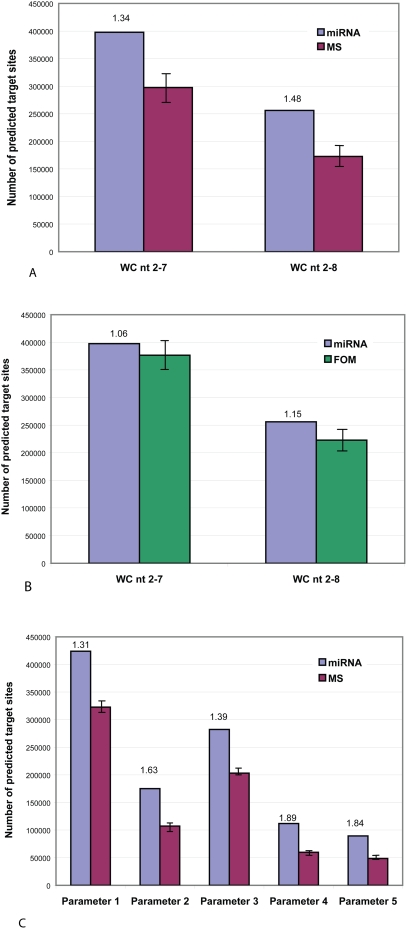
Effects of other binding parameters. Number of predicted target sites and signal-to-noise ratio of prediction with different seed lengths: WC base pairs at nucleotide positions 2–7 and 2–8 inclusive, (A) on mononucleotide-shuffled (MS) controls, and (B) on first-order Markov (FOM) controls. (C) Number of predicted target sites (blue bars) and signal-to-noise ratio (red bars) when different parameters are used, based on MS controls. Parameter 1: WC base pairs with at most one GU pair within nt 2–7, plus <6 mismatches and GU pairs at nt ≥15. Parameter 2: WC base pairs within nt 2–7, plus <6 mismatches and GU pairs at nt ≥15. Parameter 3: WC base pairs with at most one GU pair within nt 2–8, plus <6 mismatches and GU pairs at nt ≥15. Parameter 4: WC base pairs within nt 2–8, plus <6 mismatches and GU pairs at nt ≥15. Parameter 5: WC base pairs within nt 2–8, maximum of one loop at nt 9–14, plus <6 mismatches and GU pairs at nt ≥15. A 40% free energy threshold was applied in every case.

We examined the effect of binding parameters at the 3′ end of miRNAs by constraining the total number of mismatches and GU pairs at nt positions ≥15 to be <6 (Figure 6). Except at the weakest energies this improved the mean S∶N ratio, particularly as calculated against the MS control set (Figure 6C *versus* D), although at the cost of a ∼50% reduction in number of predicted targets. However, 13 of 40 of the miRNA target sites identified by our approach and that were previously validated by others (Table 1) show >6 (mismatches and GU pairs) beyond nt position 15 (Table 1). Grimson *et al*. (2007) have likewise reported that additional WC matching at positions 12–17 (especially 13–16) enhances miRNA targeting [24].

### Other parameters of binding affect the number and signal:noise ratios of predicted target sites

Identifying the seed as nt positions 2–8 instead of positions 2–7 decreased the number of predicted targets but improved the S∶N ratio of prediction by around 10% (Figure 6A–B). As allowing one GU pair in the seed region very greatly increased the number of potential target sites, for each miRNA we ordered our target-site predictions by free energy score and restricted our examination to those scoring ≥40% of the best score observed (Figure 6C). The results demonstrate that allowing up to one GU pair in the seed region increases the number of predicted targets (parameter conditions 1 and 3 above), but decreases the S∶N ratio of prediction; applying more-stringent conditions improves the S∶N ratio; and allowing only a single loop in the middle of the binding site does not affect the S∶N ratio (although many predicted sites contain such a loop). These results are summarized in Supplementary Table S1.

### Energetically favorable binding sites are not restricted to 3′UTRs of mRNAs

For short interfering RNAs (siRNAs), whether a target site is located within a translated region or a non-coding region has only marginal effects on RNA interference [25]. Plant miRNAs bind with near-perfect complementarity at sites that are primarily, though not exclusively, located within the CDS [26]. In animals, all previously validated miRNA target sites lie in the 3′UTR, and searches are often, although not always [2], restricted to 3′UTR data; modest targeting on CDS has, however, been reported [27]. For this set of known human and mouse miRNAs, however, the S∶N ratios of our predicted binding sites are very similar, regardless of location in the 5′UTR, CDS or 3′UTR (Table 2 and Table 3). Energetically favorable binding sites were more-frequent in the CDS than in any other region even after normalization by relative size (nt) of region. Predicted sites were slightly under-represented in 3′UTRs relative to mRNA length, perhaps due to their lower GC content, but show a higher S∶N ratio regardless of parameterization. Thus energetically favorable candidate target sites may occur in all mRNA regions. Interestingly, a recent report shows that in mouse, miRNAs regulate cell development through target sites in CDS regions [28].

**Table 2:** Number of predicted target sites and signal-to-noise (S∶N) ratio in different mRNA regions in human, under four sets of defining conditions (A–D).

Condition		Total mRNA	5′UTR	Coding regions	3′UTR
A	miRNA	397673	23302	250028	124343
			5.86%	62.87%	31.27%
	S∶N (MS)	1.34	1.07	1.33	1.43
	S∶N (FOM)	1.06	1.05	1.05	1.06
	DB size		7.21%	56.68%	36.11%
	DB GC		52.95%	51.99%	40.46%
B	miRNA	175207	10397	113534	51276
			5.93%	64.80%	29.27%
	S∶N (MS)	1.63	1.27	1.62	1.77
	S∶N (FOM)	1.23	1.19	1.23	1.24
	DB size		7.21%	56.68%	36.11%
	DB GC		52.95%	51.99%	40.46%
C	miRNA	256092	14541	162466	79085
			5.68%	63.44%	30.88%
	S∶N (MS)	1.48	1.18	1.48	1.57
	S∶N (FOM)	1.15	1.05	1.14	1.19
	DB size		7.21%	56.68%	36.11%
	DB GC		52.95%	51.99%	40.46%
D	miRNA	111981	6476	73147	32358
			5.78%	65.32%	28.90%
	S∶N (MS)	1.87	1.48	1.89	1.93
	S∶N (FOM)	1.33	1.23	1.33	1.35
	DB size		7.21%	56.68%	36.11%
	DB GC		52.95%	51.99%	40.46%

Database (DB) size is the proportion of nucleotides in RefSeq mRNA database (see text). Conditions: **A**, WC base pairs at nt 2–7; **B**, WC base pairs at nt 2–7, <6 mismatches-and-GU-pairs at nt ≥15, and 40% FE threshold; **C**, WC base pairs at nt 2–8; and **D**, WC base pairs at nt 2–8, <6 mismatches-and-GU-pairs at nt ≥15, and 40% FE threshold.

**Table 3:** Number of predicted target sites and signal-to-noise (S∶N) ratio in different mRNA regions in mouse, under the same four sets of defining conditions (A–D) as for human (Table 2).

Condition		Total mRNA	5′UTR	Coding regions	3′UTR
A	miRNA	287040	15577	183912	87551
			5.43%	64.72%	30.50%
	S∶N (MS)	1.36	1.08	1.35	1.45
	S∶N (FOM)	1.12	0.96	1.13	1.13
	DB size		6.77%	58.10%	35.13%
	DB GC		48.25%	51.35%	39.18%
B	miRNA	114035	6326	74562	33147
			5.55%	65.39%	29.07%
	S∶N (MS)	1.68	1.63	1.64	1.78
	S∶N (FOM)	1.27	1.20	1.27	1.28
	DB size		6.77%	58.10%	35.13%
	DB GC		48.25%	51.35%	39.18%
C	miRNA	187673	9950	121003	56720
			5.30%	64.48%	30.22%
	S∶N (MS)	1.56	1.26	1.55	1.66
	S∶N (FOM)	1.16	1.01	1.17	1.17
	DB size		6.77%	58.10%	35.13%
	DB GC		48.25%	51.35%	39.18%
D	miRNA	74455	4018	48979	21458
			5.40%	65.78%	28.82%
	S∶N (MS)	1.94	1.56	1.94	2.04
	S∶N (FOM)	1.34	1.23	1.34	1.36
	DB size		6.77%	58.10%	35.13%
	DB GC		48.25%	51.35%	39.18%

Database (DB) size is the proportion of nucleotides in RefSeq mRNA database (see text). Conditions: **A**, WC base pairs at nt 2–7; **B**, WC base pairs at nt 2–7, <6 mismatches-and-GU-pairs at nt ≥15, and 40% FE threshold; **C**, WC base pairs at nt 2–8; and **D**, WC base pairs at nt 2–8, <6 mismatches-and-GU-pairs at nt ≥15, and 40% FE threshold.

**Figure 7:**
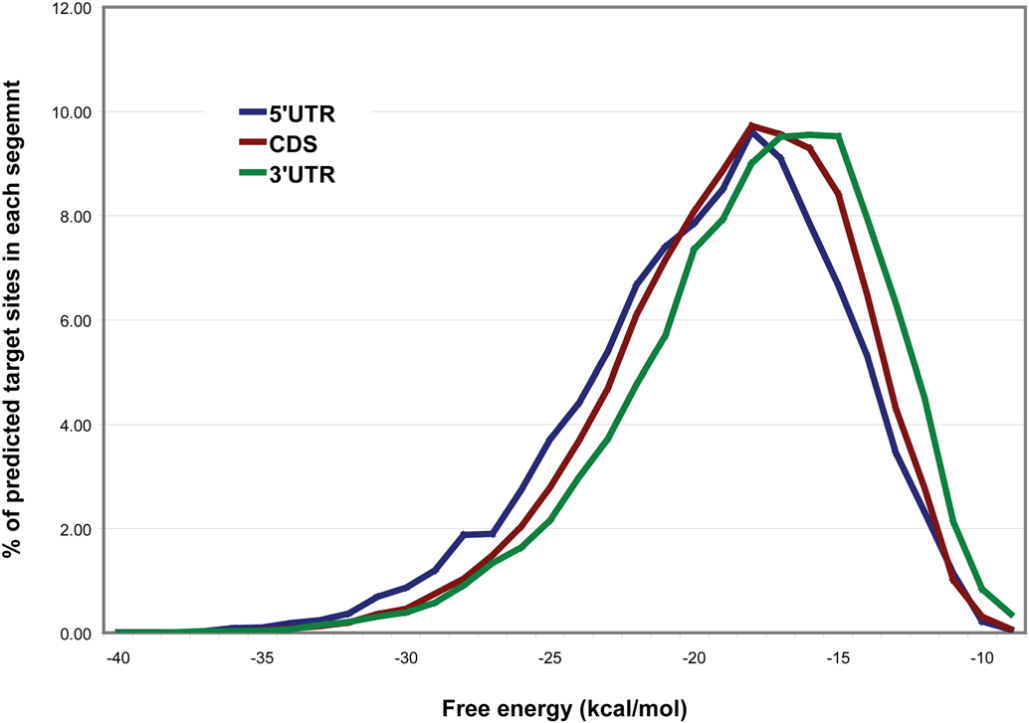
Proportion of predicted miRNA target sites (for 313 human miRNAs) as a function of free energy score, in each segment (5′ UTR, CDS and 3′ UTR) of human RefSeq mRNAs. Line smoothed for ease of interpretation.

Evidence has been presented that for miRNAs with perfectly WC-complementary 8-nt seed regions, target sites in the 3′UTR are more efficacious in depressing mRNA levels than are sites in the 5′UTR or CDS [24]. Efficacious sites tend to be flanked ∼30 nt both upstream and downstream by AU-enriched regions [24]. Our data confirm that predicted target sites in higher-GC regions (5′UTR and CDS) have lower (more-favorable) energy scores than our predicted target sites in lower-GC regions (3′UTRs) (Figure 7). Higher-GC regions are expected to contain more-stably folded local structure, whereas sites in lower-GC regions may be, on average, more accessible. As a consequence, miRNAs targeting lower-GC regions may form duplexes at relatively weaker energies and still be functional, whereas those targeting the 5′UTR or CDS may require more-favorable energies to compete with pre-existing structure, and this may require hybridization that extends well beyond the seed region and near perfect complementary binding to the target sites. An approach based on hybridization energy, such as ours, tends to give prominence to target sites in higher-GC regions such as 5′UTR or CDS. Optimizing the prediction of target sites in regions of more-stably folded local structure (*i.e.* higher-GC regions) may therefore require more-strict conditions at the secondary filtering stage.

### Individual binding sites are predicted in the coding region or the UTR, or be absent altogether, depending on mRNA isoform

As described immediately above, many potential energetically favorable binding sites can be found in 5′UTRs and coding regions, as well as in the 3′UTRs. RefSeq (www.ncbi.nlm.nih.gov/RefSeq) identifies 2943 protein-coding genes as having more than one transcriptional isoform in human; these 2943 genes are represented by 7965 RefSeq mRNAs, among which we predict no miRNA binding site in any transcript of 64 genes. Removing these 64 genes and their 146 transcripts, we are left with 66381 predicted binding sites distributed across 7819 transcript isoforms of 2879 multi-transcript human genes. For each of these genes in turn, we next asked whether a predicted binding site was found in the same mRNA region in every transcript isoform, reassigned to a different mRNA region in at least one isoform, or lost altogether in at least one isoform (Table 4).

**Table 4:** Numbers of predicted target sites reassigned to a different mRNA region, or gained or lost, among different isoforms of the same transcriptional region (gene), for the 2943 human protein-coding genes annotated (RefSeq) as having more than one transcriptional isoform, under six sets of defining conditions (A–F).

Condition	WC nt =	Overlap exclude at edge of CDS (# nt)	Number of:	Genes with ≥2 isoforms	Genes with ≥2 isoforms, and with target sites	Target sites remaining in same region	Target sites reassigned among regions	Target sites gained/lost in one isoform
A	2–7	None	Unique genes	2943	2879	2658	777	2022
				1947	1896	1811	409	1213
				996	983	847	368	809
			Isoforms	7965	7819	7023	2485	5823
				3894	3792	3622	818	2426
				4071	4027	3401	1667	3397
			Target sites	66381	66381	44972	6519	14890
				32422	32422	25540	1946	4936
				33959	33959	19432	4573	9954
B	2–8	None	Unique genes	2943	2783	2435	574	1720
				1947	1828	1669	295	1010
				996	955	766	279	710
			Isoforms	7965	7579	6386	1885	5044
				3894	3656	3338	590	2020
				4071	3923	3048	1295	3024
			Target sites	42690	42690	28717	4251	9722
				20726	20726	16334	1252	3240
				21964	21964	12383	2999	6582
C	2–7	5	Unique genes	2943	2877	2658	777	2021
			Isoforms	7965	7815	7023	2485	5821
			Target sites	66271	66271	44908	6511	14852
D	2–7	10	Unique genes	2943	2877	2658	777	2020
			Isoforms	7965	7815	7023	2485	5819
			Target sites	66166	66166	44831	6501	14834
E	2–8	5	Unique genes	2943	2782	2435	574	1718
			Isoforms	7965	7577	6386	1885	5039
			Target sites	42629	42629	28683	4243	9703
F	2–8	10	Unique genes	2943	2782	2435	574	1718
			Isoforms	7965	7577	6386	1885	5039
			Target sites	42557	42557	28622	4243	9692

All defining conditions include <6 mismatches-and-GU-pairs at nt ≥15, and 40% FE threshold, but differ by extent of base-paired region, and by correction (or not) for edge effects. In blocks A–B, the top number in each cell refers to total; the middle number, to genes with exactly two transcriptional isoforms; and the bottom number, to genes with ≥3 transcriptional isoforms. “Target sites remaining in same region” means neither reassigned, nor gained or lost, among different isoforms.

Using the parameterization that yields the highest S∶N ratio (Figure 6C), in this set we found 2658 genes (92.3% of 2879) with 7023 isoforms (89.8% of 7819) in which at least one predicted miRNA target site remains in the same mRNA region in every isoform; 44972 target sites (67.7% of 66381) thus resist reassignment or loss across known transcript isoforms. We found 777 genes (27.0%) with 2485 isoforms (31.8%) in which at least one predicted target site is reassigned to a different mRNA region in at least one isoform, with 6519 (9.8%) of predicted miRNA targets in this category; most of these cases involve a site found in the 5′UTR of one isoform but in the CDS of a second (*e.g.* the energetically most-favorable miR-17-5p target site on TNFSF12 mRNAs: Supplementary Figure S1), or in the CDS of one but in the 3′UTR of a second, although in a small number of isoform sets a binding site is reassigned among all three regions. Finally, in 2022 genes (70.2%) with 5823 isoforms (74.5%) at least one predicted target site is lost (or gained) among different isoforms; 14890 target sites (22.4%) are in this category. Clearly, many genes (thus many sets of transcripts) contain target sites that fall into more than one of these situations. A proportionally similar distribution of fates is seen whether the seed region is taken to be nt 2–7, or nt 2–8 (Table 4).

It is interesting to ask how these frequencies of predicted target-site reassignment and gain/loss among transcript-isoform sets compare with the corresponding frequencies for nucleotides (whether associated with a predicted miRNA target site or not). Calculation of nucleotide reassignment and gain/loss is straightforward for transcript sets with only two isoforms (see Methods), although more-complicated for larger isoform sets. Of these 2943 genes with multiple isoforms, 1947 have only two isoforms and the other 996 have three or more (range 3 to 23). Among these 1947, on average 3.4% of nucleotides are reassigned among mRNA regions, while nucleotide loss averages 9.8% based on the longer isoform and 18.5% based on the shorter (Supplementary Table S3). By contrast, for predicted target sites we observe 6.0% reassignment and 15.2–15.6% gain/loss among transcript sets for genes with only two isoforms (Table 4). Correction for edge effects scarcely alters these proportions (Table 4 and Supplementary Table S3). Thus the observed frequency of target site reassignment exceeds that expected under a length-proportional model based on these data, whereas the frequency of gain/loss may not be significantly different from expectation.

### miRNA targets with orthologs in mouse

One motivation for this work has been to predict miRNA target sites (and thus the mRNAs in which these sites exist) without taking into account their conservation across different species. However, most known miRNAs and many validated target sites are conserved across species, and we can use this information to improve our prediction accuracy.

Using those 181 pairs of miRNAs orthologous between human and mouse for which sequence positions 1–8 are identical, we predicted target sites using two parameterization conditions and the same sets of randomized human sequences as controls in each case. Requiring orthology improves the S∶N ratio (1.87 compared to 1.50 for one parameterization, 2.97 compared to 1.81 for the other against MS controls; 1.81 compared to 1.21 for one parameterization, 2.61 compared to 1.34 for the other against FOM) although at the cost of a 72–81% reduction in number of predicted sites (Table 5).

**Table 5:** Total numbers of predicted targets and signal-to-noise (S∶N) ratios among human (RefSeq) mRNAs for the 181 human miRNAs with an ortholog in mouse, with and without requiring that the orthologous mouse miRNA have a target (under the same second-stage criterion) in the orthologous mouse mRNA.

Control set	Condition	Number of targets	S∶N ratio	Fold change
MS	WC bp (in human)	268375	1.50	(1.00)
	WC bp with match in orthologous mouse mRNA	74232	1.87	+0.25
	WC bp, no match in orthologous mouse mRNA	194143	1.40	−0.07
	WC bp and 40% FE threshold (in human)	116117	1.81	(1.00)
	WC bp and 40% FE threshold, with match in orthologous mouse mRNA	21574	2.97	+0.64
	WC bp and with 40% FE threshold, no match in orthologous mouse mRNA	94543	1.66	−0.08
FOM	WC bp (in human)	268375	1.21	(1.00)
	WC bp with match in orthologous mouse mRNA	74232	1.81	+0.50
	WC bp, no match in orthologous mouse mRNA	194143	1.08	−0.11
	WC bp, <6 mismatches and GU pairs at nt ≥15 and 40% FE threshold (in human)	116117	1.34	(1.00)
	WC bp, <6 mismatches and GU pairs at nt ≥15 and 40% FE threshold, with match in orthologous mouse mRNA	21574	2.61	+0.95
	WC bp, <6 mismatches and GU pairs at nt ≥15 and with 40% FE threshold, no match in orthologous mouse mRNA	94543	1.21	−0.10
Lewis	WC bp (in human)	112797	1.15	(1.00)
	WC bp with match in orthologous mouse mRNA	28456	1.69	+0.47
	WC bp, no match in orthologous mouse mRNA	84341	1.04	−0.10
	WC bp, <6 mismatches and GU pairs at nt ≥15 and 40% FE threshold (in human)	46469	1.44	(1.00)
	WC bp, <6 mismatches and GU pairs at nt ≥15 and 40% FE threshold, with match in orthologous mouse mRNA	8684	2.66	+0.85
	WC bp, <6 mismatches and GU pairs at nt ≥15 and with 40% FE threshold, no match in orthologous mouse mRNA	37785	1.30	−0.10

Watson-Crick base pairs (WC bp) are at nucleotide positions 2–7 in every case. MS, mononucleotide shuffle; FOM, first-order Markov process; Lewis, control sequences from Lewis *et al.* [2] but based on only 74 miRNAs.

Under these two conditions, 83–87% of the predicted target sites present in orthologous mRNAs (human and mouse) occur in the same mRNA region (*e.g.* 3′UTR); these values are uncorrected for the few cases in which an mRNA in one species is annotated as having two or more orthologs in the other. To a first approximation, miRNA target sites in the same region of orthologous mRNAs can be considered orthologous sites. Thus most miRNA target sites present in homologous mRNAs between human and mouse are themselves orthologous.

miRNA sequences can be highly conserved across different species, but this does not imply that their target sites are necessarily similarly conserved in sequence. Under these two parameterizations, 72–81% of predicted target sites do not have a counterpart in an orthologous mRNA (Table 5). Many taxon-specific miRNA-mRNA interactions may be thus available to regulate taxon-specific developmental processes in human and mouse.

### Method evaluation

In our hands, FASTH is about 30-fold faster than RNAhybrid version 2.1 [16] in finding target sites for a single miRNA among 500 mRNAs (∼1.5 Mbp); on a single AMD64 core (1 Gb memory) FASTH completes this in 4 seconds, and RNAhybrid in 125 seconds. RNAplex version 1.0.0 is claimed to be 10–27 times faster than RNAhybrid [17] but could not process even our 500-mRNA database, as it cannot deal with large sequences (>∼4100 nt) at least on the above-mentioned hardware. Search time scales linearly with database size for RNAhybrid and RNAplex, whereas with FASTH it scales sub-linearly (see Supplementary Text).

We examined the efficiency of our approach in recovering those miRNA target sites that have been experimentally validated in human [23] (Table 1) or mouse [9]. Of 82 miRNA binding sites experimentally validated in human (all of which are in the 3′UTR), 65 occur in RefSeq and were available for discovery (see Methods). Of these we recovered 40 using the most permissive search criterion (Table 1), *i.e.* recall was 62%. However, many of these 40 have poor free energy scores, and about half failed to meet one or more of the secondary filtering criteria we used to improve the S∶N ratio.

To provide a broader test, we applied the same computational approach to the RefSeq mouse mRNA database (see Methods), asking what proportion of the target sites experimentally validated by Miranda *et al*. [9] we recovered. As Miranda *et al.* set different criteria for target prediction (*e.g.* allowing GU pairs and a mismatch in the seed regions) and selected sites for validation on that basis, not all of their validated sites could have been discovered by our approach; we correct for this difference, to the extent possible, by removing from consideration those targets (in [9]) that have GU pairs and/or mismatches in the seed region. Miranda *et al.* also give details of their predicted sites that failed validation (*i.e.* were false positive predictions); in principle this makes it possible for us to calculate the sensitivity as well as specificity of our approach (see Methods). On this basis, our approach shows high specificity (range 0.50–0.93) but moderate sensitivity (range 0.33–0.80 depending on miRNA and filtering criteria) (Table 6). As the number of false positives is small and the data apply to mouse only, these specificity values should be interpreted as only indicative for our approach.

**Table 6:** For three miRNAs, our predictions (condition: perfect Watson-Crick complementarity at nt 2–7) on targets experimentally validated by Miranda *et al*. [9].

miRNA	Condition	Number of targets		
miR-134	WC bp at nt 2–7	True positives	43	Sensitivity = 0.551
		False negatives	35	Specificity = 0.666
		False positives	3	
		True negatives	6	
		Total	87	
	WC bp at nt 2–7, and 40% FE threshold (−18.64)	True positives	36	Sensitivity = 0.462
		False negatives	42	Specificity = 0.666
		False positives	3	
		True negatives	6	
		Total	87	
miR-296	WC bp at nt 2–7	True positives	8	Sensitivity = 0.80
		False negatives	2	Specificity = 0.50
		False positives	1	
		True negatives	1	
		Total	12	
	WC bp at nt 2–7, and 40% FE threshold (−19.44)	True positives	7	Sensitivity = 0.70
		False negatives	3	Specificity = 0.50
		False positives	1	
		True negatives	1	
		Total	12	
miR-375	WC bp at nt 2–7	True positives	9	Sensitivity = 0.375
		False negatives	15	Specificity = 0.929
		False positives	1	
		True negatives	13	
		Total	38	
	WC bp at nt 2–7, and 40% FE threshold (−16.68)	True positives	8	Sensitivity = 0.333
		False negatives	16	Specificity = 0.929
		False positives	1	
		True negatives	13	
		Total	38	

Of 158 genes experimentally tested for regulation by miR-134, 85 occur in our database, as do 14 of 24 tested for regulation by miR-296, and 22 of 44 tested for regulation by miR-375. As Miranda *et al.* set different criteria for target prediction (*e.g.* allowing GU pairs and a mismatch in the seed regions) and selected sites for validation on that basis, not all of their validated sites could have been discovered by our approach. To correct (to the extent possible) for this difference, we excluded a further 20 target sites with GU pairs and mismatches in the seed region for miR-134, and 8 target sites for miR-296 (all target sites for miR-375 have WC matches in the seed region), and report the results of 65 target genes examined for miR-134, 6 for miR-296 (for miR-296, all six sites examined were validated), and 22 for miR-375. Miranda *et al.* also give details of predicted sites that failed validation (*i.e.* were false positive predictions); in principle this makes it possible for us to calculate the specificity of our approach. In some cases, our 40% free energy threshold is more-stringent than that of Miranda *et al*. (∼−16.4 kcal/mol), reducing the sensitivity calculated for our approach. As the number of false positives is small, specificity values should be interpreted as indicative only.

### Comparison with other methods

We compared our predicted target sites to those identified using the commonly used methods PicTar [14], TargetScan [11] and MiRanda [13], restricting our comparison to 3′UTR regions of human mRNAs. Overlap between our predicted target sites and those of the other methods was 15–19% with PicTar, 13–16% with TargetScan, and 4–5% with MiRanda depending on the parameter values used in our second-stage filtering. The overlap with MiRanda is smaller in part because the number of targets predicted using MiRanda is smaller (22,896) than with PicTar (61,820) or TargetScan (44,657) (see Supplementary Table S4). Pairwise overlaps among the other three methods ranged from 8% (PicTar *vs* MiRanda) to 55% (PicTar *vs* TargetScan) (Supplementary Table S5). Substantial overlap between predictions of PicTar and TargetScan has been observed by others [21].

### Experimental validation

The Gene Ontology [29] terms most-enriched among our lowest-energy targets include *protein kinase cascade*, *signalling pathway*, *negative regulator of transcription*, and *anti-apoptosis* (Supplementary Table S6).

We selected, for experimental validation, targets to three of these miRNAs: hsa-miR-17-5p (one target: see below), hsa-miR-15a (two targets), and hsa-miR-324-3p (three targets). This selection was made on the basis of functional association with cancer (hsa-miR-17-5p, hsa-miR-15a) or predicted targets in the Wnt signalling pathway (hsa-miR-324-3p) as described in Supplementary Table S7. Sequence corresponding to individual predicted target sites was cloned into the 3′UTR of a luciferase-expressing vector, and luciferase activity (directly proportional to translation from the plasmid) was measured in the presence of either the test miRNA, or a control miRNA-like sequence. For replication, normalization and statistical analyses see Methods.

**Figure 8:**
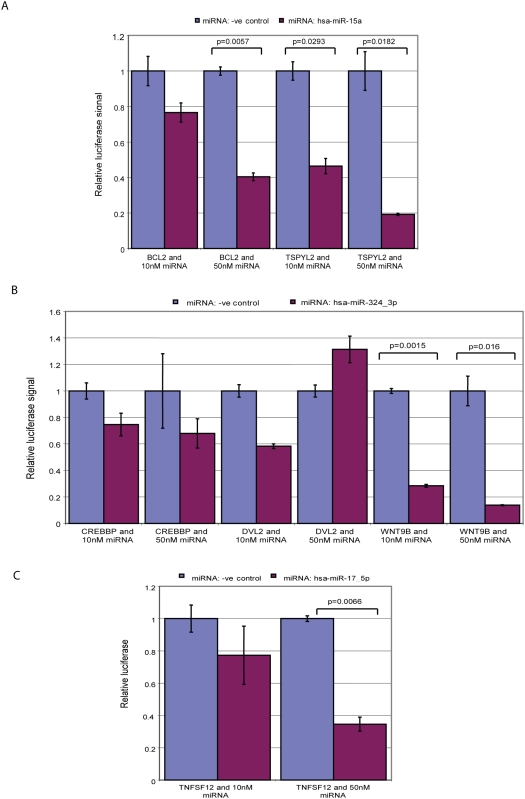
Experimental validation of predicted target sites. Y-axis: relative luciferase activity (see Methods). (A) has-miR-15a on TSPYL2 and BCL2; (B) has-miR-324-3p on CREBBP, DBL2 and WNT9B; and (C) has-miR-17-5p on TNFSF12.

For five of the six targets subjected to experimental validation, expression was inhibited by the corresponding miRNA (Figure 8). Assessed by *p*-value, this decrease was significant for the TSPYL2 and WNT9B constructs at 10 and 50 nM, and for the BCL2 and TNFSF12 constructs at 50 nM. BCL2 mRNA has previously been validated as a target for has-miR-15a inhibition [30]. The CREBBP construct showed ∼32% mean reduction at 50 nM, although this reduction is not significant as assessed by *p*-value. The sixth mRNA construct, for DVL2, showed > 40% mean reduction with small variance at 10 nM (p<0.467), but an increased expression at 50 nM (Figure 8B).

Five of the target sites (hsa-miR-15a/TSPYL2, hsa-miR-15a/BCL2, hsa-miR-17-5p/TNFSF12, hsa-miR-324-3p/CREBBP and hsa-miR-324-3p/WNT9B) exhibit perfect WC complementarity in the seed regions, while has-miR-324-3p/DVL2 has one GU pair in the same region (Supplementary Figure S1). Although the binding site of hsa-miR-324-3p/DVL2 has very low (stable) free energy, the presence of a GU pair in the seed region means that binding at the 3′ end of the miRNA would have to be optimal to compensate (if indeed 3′-end hybridization can compensate at all); in this case, however, two unpaired bases and two GU pairs are predicted in the 3′ end, blocking even the possibility of adequate compensation.

## Discussion

### Methodological approach

Computational prediction may be motivated by different aims, *e.g.* to annotate sequences, to guide experimental validation, or to describe a potential solution space. Common to these is the goal of generating a ranked list of candidates that contains most (ideally all) true interactions and excludes most (ideally all) non-interactions. Biological relevance may, however, be contingent on features other than those being maximized or minimized. Animal miRNAs, for example, often bind to mRNAs with suboptimal complementarity as assessed by string-matching [1] because *in vivo*, potential binding sites are arrayed dynamically within double-stranded regions, single-stranded loops, and bulges that are imperfectly represented as strings but nonetheless contribute to duplex stability. Other features, *e.g.* post-translational editing of miRNAs and spatiotemporal co-location within the cell, may be even more-difficult to represent, or be unknown.

Here we present a scalable approach and software for predicting miRNA-mRNA interactions and interaction sites, based on minimizing the free energy of duplex structure. Our method finds and can report not only the energetically optimal duplex, but also arbitrarily many energetically suboptimal structures, in genome-scale data. In the specific context at hand, for each miRNA we initially identify a large set of candidate sites, and subsequently remove those that fail to meet additional criteria that reflect biologically relevant parameters of known or inferred miRNA-mRNA interactions. In other contexts, the FASTH software might better be paired with machine-learning or other technologies, *e.g.* to assess the relative contributions of specified parameters.

### Short regions perfectly complementary to mRNA targets are preferentially found in the 5′ end of miRNAs

In many validated miRNA-mRNA interactions, regions of 6–7 contiguous nucleotides having (near-)perfect WC complementarity to the mRNA target occur at the 5′ end of the miRNA. Our results confirm this observation, and extend it to energetically favorable potential target sites not only for validated interactions but more broadly. Sequence motifs complementary to the 5′ end of miRNAs are preferentially distributed in mRNAs, compared to motifs that are complementary to the 3′ end.

### Energetically favorable binding sites are not restricted to 3′UTRs

Miranda *et al*. predicted that a significant number of targets occur in 5′UTRs and CDS and hypothesized that many are functional, although they could not estimate a false positive rate of prediction [9]. We likewise find many high-quality binding sites in all mRNA regions (5′UTR, CDS and 3′UTR), with the number of sites and S∶N ratio of prediction stable over the range of filtering parameters examined. For the human and mouse transcriptomes, target abundance roughly matches availability (length of mRNA regions). As false-positive as well as true-positive target sites in higher-GC regions tend to have lower (more-favorable) energies (above), additional filtering or weighting may be required to predict target sites in these regions.

### Binding sites are often reassigned or lost in alternative transcript isoforms

At least in mouse [31] and human [32], most protein-coding genes are transcribed in different isoforms. Here we report that among isoform sets of multi-transcript human genes, almost 10% of predicted miRNA target sites are situated in one mRNA region in one isoform but in a different region in another isoform, *i.e.* reassigned between isoforms, as the result of differential splicing. Within those transcript isoform sets having exactly two isoforms, the frequency of such reassignment is some 76% (6.0%/3.4%) greater than expected under a simple transcript length-proportional model. In almost all such cases, reassignment is observed between the 5′UTR and CDS, or between the CDS and 3′UTR. We also find that more than 20% of our predicted miRNA target sites are present in some but not all isoforms that arise from the same transcriptional region. As many more mRNA isoforms exist than are represented in existing databases, the instances of miRNA binding site reassignment or gain/loss that we predict here almost certainly under-represent the true number in human (and, by extension, other complex animals) that arise as a consequence of transcriptional complexity.

### Implication for transcriptional regulation

Some investigations have tried to capture the intersection of miRNA and transcriptional regulation. For example, Stark *et al*. reported that in *Drosophila*, miRNAs and genes encoding predicted miRNA targets are expressed in a largely mutually exclusive manner, and that many genes for basic cellular processes have short 3′UTRs and thereby avoid miRNA regulation [33]. Farh *et al*. reported that mRNAs expressed in the same tissue as miRNAs are selectively depleted in sites that match these miRNAs [34].

Our results show that high-quality potential miRNA binding sites exist in all regions of mRNAs. Indeed, in a complex transcriptional program such as that of human or mouse, there is some ambiguity in assigning a target site to a specific mRNA region; as shown above, potential target sites are frequently reassigned among mRNA regions, or indeed lost or gained entirely, in alternative transcript isoforms, opening the possibility that miRNAs can mediate transcript-specific regulation, *e.g.* in different cell types, tissues or developmental conditions. The observed frequency of site involvement in gain/loss (22.4% of predicted sites) appears somewhat greater than expected under a simple length-proportional model (a maximum of 18.5% of nucleotides are similarly gained/lost, based on the shorter transcript in pairwise comparisons); further analysis is required to determine whether this difference is real, and if so whether it might reflect positive selection in these transcriptional regions or a subset thereof. For one of the experimentally validated miRNAs in this study, miR-17-5p, we predicted target sites in both known transcript isoforms of TNFSF12, but in the CDS of one and the 3′UTR of the other.

### Most predicted miRNA targets in human and mouse have no counterpart in the orthologous mRNA of the other species

Predicted miRNA targets in human mRNAs that have orthologs in mouse show a higher S∶N ratio than do targets in human mRNAs that lack mouse orthologs. Of these predicted sites, most (83–87%) occur in the same mRNA region in both species, *i.e.* most miRNA targets in orthologous mRNAs are in this sense themselves orthologous. However, limiting our analysis to mRNAs with orthologs in the other species diminishes the number of predicted sites by 72–81%: most potential miRNA target sites found by searching only human have no counterpart in an orthologous mouse mRNA (and vice-versa), implying that many miRNA-mRNA interactions are potentially taxon-specific. Similar or greater ratios of genome-specific to evolutionarily conserved potential target sites have been inferred by others [34].

### Experimental validation of miRNA-mRNA interaction

We selected, for experimental validation, six miRNA-mRNA interactions predicted by our method, including one previously validated interaction [29] and another identified as a possibility [14]. Using a luciferase assay in HEK293 cells, we showed that four of these six transcripts are bound by endogenous miRNAs, with the corresponding mRNA level reduced by an average of 73% (range 59.6%–86.2%) when assayed at 50 nM miRNA. These four include the previously validated interaction, plus three of the five (60%) newly predicted interactions for which the mRNA level was reduced by an average of 77.5% (range 65.4%–86.2%) when assayed at 50 nM miRNA. A fifth construct showed a ∼32% mean reduction, which could be biologically (functionally) relevant but falls short of statistical significance as assessed by the *p*-value criterion.

Regardless of where the predicted target site is located in the mRNA, in our experimental protocol the (synthetic) site was inserted into the construct so it could be expressed from the 3′UTR of luciferase mRNA. Thus we can validate that a target site is bound by endogenous miRNA and that the mRNA level is reduced as a consequence, but we do not specifically validate interaction with the site in its original location or native folded environment. Duplex formation is prerequisite to biological function, but these assays examine only the effect of binding on mRNA level, not its functional consequences.

### Conclusions

It has been an open question whether low (strong) energy of the miRNA-mRNA duplex can serve as a good indicator of miRNA targets. We have developed and implemented an energy-based computational approach to miRNA target prediction, applied it to a standard set of known human mRNA sequences at whole-transcriptome scale, and experimentally measured the change in mRNA level for a small number of selected predictions. We showed that short (6–7 nt) sequence motifs matching mRNAs with perfect WC complementarity occur more frequently in the 5′ end of miRNAs than in the 3′ end, and that high-quality binding sites are present in all regions of mRNA (5′UTR, CDS and 3′UTR) in both human and mouse. We also present evidence that many high-quality potential miRNA binding sites become located in different mRNA regions, or are gained or lost altogether, depending on transcript isoform. These observations open the possibility that further complexity of genetic regulation arises at the interface among miRNAs, expression and alternative splicing in morphologically complex animals. Future directions include incorporating predicted mRNA structure at the target site, miRNA and mRNA expression levels, and correlations with protein expression and phenotype.

## Materials and Methods

### Data

313 mature human and 233 mature mouse miRNA sequences were obtained from miRBase release 7.0 [35] (www.sanger.ac.uk/software/Rfam) and are available as Supplementary Table S8. mRNA and gene data were obtained from hgdownload.cse.ucsc.edu/downloads.html (file mrnaRefseq.txt) and represent 22947 unique mRNAs and 17751 protein-coding genes in human, and 17510 and 16627 in mouse. mRNAs were mapped to gene identifiers using the files refFlat and refLink also from UCSC, and coordinates of miRNA genes and miRNA targets were assigned based on the NCBI Human (hg17, Build 35) and Mouse (mm6, Build 34) Genome Sequencing Consortium genome builds. Relative sizes of mRNA regions (5′UTR, CDS and 3′UTR) were calculated using annotations in the files rna.gbff downloaded from NCBI (ftp.ncbi.nih.gov/refseq).

### Computational approach

We implemented a two-stage prediction process. First we used FASTH to search for potential targets in the mRNAs by duplex free energy, and to rank the results by energy. Then in a second stage we remove (filter) results that fail to meet further criteria not expressed in terms of energy score *per se*, *e.g.* the minimum number of contiguous Watson-Crick base pairs in the so-called seed region, or the maximum number of unpaired bases, bulges and GU pairs. This second-stage filtering was implemented outside FASTH *via* Perl scripts. Detailed descriptions of our computational approach, including FASTH, are presented in Supplementary Text. Our target-site predictions with selected parameters in human and mouse are available as Supplementary Table S9, Supplementary Table S10, Supplementary Table S11, and Supplementary Table S12.

### Statistical description of results

To estimate the false positive rate of prediction we calculate a signal-to-noise (S∶N) ratio, dividing the number of target sites predicted using known (empirical) miRNAs by the mean number predicted using controls. Our motivation for generating these controls by two different approaches, mononucleotide shuffling (MS) and a first-order Markov process (FOM), is presented in the Supplementary Text. For each known miRNA, the MS control set was constructed by random permutation, without replacement, of its nucleotides, preserving both length and nucleotide composition (but not dinucleotide frequencies, except by chance). The FOM control set, generated using Sean Eddy's ‘shuffle’ program in the Squid package (http://selab.janelia.org/software.html), preserves length and dinucleotide frequencies (but not mononucleotide count, except by chance). For each miRNA we generated 10 MS and 10 FOM control sequences. In all, 2× 3130 control sequences were generated for human, and 2× 2330 for mouse. These control sequences were then used to search against the target database under the same second-stage filtering conditions as for the real miRNA. Results are presented in Table 5 and Supplementary Table S1.

We additionally used, as a further set of controls, the shuffled sequences generated by Lewis *et al*. so as to preserve the expected frequency of random matching between miRNA seed sequences and complementary 3′UTR sequences [11]. Results are presented in Table 5 and Supplementary Table S2.

Sensitivity is calculated as true positives/(true positives + false negatives), and specificity as true negatives/(true negatives + false positives).

### Mapping target locations to regions of mRNAs

Numbers of predicted targets located in the three regions of mRNAs (5′UTR, CDS and 3′UTR) were determined by retrieving sizes (lengths measured by number of nucleotides *nt*) of each region in each mRNA from the rna.gbff files for human and mouse (above), and mapping target sites to these regions using the coordinates provided.

### Modelling the expected frequencies of site reassignment and gain/loss among isoforms

Genomic coordinates of the first and last nucleotides for each transcript region (5′UTR, CDS, 3′UTR, introns if any) in human were obtained from the refFlat file downloaded from UCSC (hgdownload.cse.ucsc.edu/downloads.html). The number of nucleotides in each mRNA was computed from these coordinates. For transcriptional regions (genes) with exactly two known isoforms in this file, comparison is straightforward. For genes with ≥3 isoforms, we made all pairwise comparisons, *i.e.* a gene with four isoforms requires 4*(4−1)/2 = 6 pairwise comparisons. To simplify calculation we set transcript length (including introns) equal to that of the longest transcript, so the total number of nucleotides shown in the Intron/Intron cells is slightly exaggerated (because missing nucleotides in the shorter transcripts are counted as introns); otherwise the computation proceeded as for binary transcript sets. In summarising results (Supplementary Table S3), pairwise comparisons relative to the longer member of each mRNA pair were grouped, as were comparisons relative to the shorter member of each pair. Corresponding calculations were also made correcting for edge effects (disregarding predicted miRNA target sites that overlap any nt position within 5 or 10 nt of the edge of a miRNA region); see Supplementary Table S3 for details.

### miRNA target sites with orthologs in mouse

Most of the 233 miRNAs known in mouse have counterparts extremely similar in sequence (*i.e.* putative orthologs) among the 313 miRNAs known for human. From among these we extracted the 181 pairs that have identical sequences at positions 1–8 inclusive (Supplementary Table S13). The sequence of each human miRNA was randomized, and the S∶N ratio calculated, as described above. Given the computational complexity of the target search, we used the same sets of random sequences to search both human and mouse mRNA databases.

Ortholog pairs between the human and mouse mRNA sets were identified using BioMart (www.biomart.org). A miRNA target site (*e.g.* in human) is considered to have an ortholog in the other species (*e.g.* mouse) if the miRNAs and targeted mRNAs are orthologs in the two species. For example, the human miRNA has-miR-378 is predicted to have a target site in the mRNA corresponding to human gene BCL7A, while mmu-miR-378, the mouse ortholog of has-miR-378, has a target site in the mRNA for Bcl7a (the orthologous gene in mouse). We thus consider the has-miR-378 target site in BCL7A to have an ortholog in mouse, and infer that regulation of these genes by this miRNA has been evolutionarily conserved between human and mouse.

### GO terms

GO terms were retrieved and analysed using DAVID [36].

### Experimental validation

To determine the effect of each miRNA on its predicted targets, synthetic oligonucleotides corresponding to 60 nt around the target sequence were cloned into the *Spe*I and *Hin*dIII sites of pMIR-REPORT Luciferase (Ambion). All constructs were verified by sequencing. HEK293 cells were maintained in DMEM (GibcoBRL) containing 10% (v/v) foetal calf serum in a 5% CO_2_ atmosphere at 37°C. Cells were transfected using Effectene (Qiagen) according to manufacturer's instructions. In each well, 5×10^4^ cells were co-transfected with 100 ng of a pMIR-REPORT Luciferase construct, 100 ng of pMIR-REPORT β-galactosidase (Ambion), and either 10 or 50 nM of the appropriate pre-miR miRNA precursor (Ambion). After transfection, cells were incubated for 42 hours prior to harvesting.

Luciferase activity was measured in the presence of either the test miRNA or a control miRNA. *p*-values were derived using Student's t-test on the mean and standard deviations of the normalized luciferase signal of test miRNAs, and on the negative control miRNAs for the same reporter construct. Before averaging, luciferase activity was normalized to the corresponding signal from the β-galactosidase reporter. Luciferase activity was assayed using the Luciferase Assay System (Promega) and detected on a Wallac 1420 luminometer (Perkin Elmer). β-galactosidase activity was determined using the β-Galactosidase Enzyme Assay System (Promega) and detected on a PowerWave XS spectrophotometer (BioTek). Each assay was repeated at least three times.

Predicted mRNA targets were selected for experimental validation based on our groups' ongoing interest in cancer and in Wnt signalling, as described further in the text.

### Availability

The FASTH source code is available by request from MZ (zukerm@rpi.edu). It is configured to run in a Unix or Linux environment.

## Supporting Information

Supplementary Material S1 Description of Supplementary Material
(0.03 MB DOC)

Text S1 Specification and generation of control sequences, and technical description of approach
(0.08 MB DOC)

Figure S1 Energetically favourable miRNA-mRNA hybrid secondary structure predicted (FASTH) for each target subjected to experimental validation in this study
(0.03 MB DOC)

Table S1 Number of predicted targets and signal-to-noise ratio with different filtering parameters for native miRNAs, mononucleotide shuffled (MS) and first-order Markov (FOM) control sequences
(0.06 MB DOC)

Table S2 Number of predicted targets and signal-to-noise ratio for 74 miRNAs, compared with control sequences from the Lewis et al. supplemental material
(0.04 MB DOC)

Table S3 Numbers of nucleotides assigned to different mRNA regions (5′UTR, CDS, 3′UTR and intron), or gained or lost, among different isoforms of the same transcriptional region (gene)
(0.12 MB DOC)

Table S4 Degree of overlap between FASTH prediction sets and those of other methods
(0.05 MB DOC)

Table S5 Degree of overlap among prediction sets of three methods
(0.03 MB DOC)

Table S6 Top ten over-represented Gene Ontology terms for Biological Process (BP), Cellular Component (CC) and Molecular Function (MF) among mRNAs predicted as miRNA targets
(0.07 MB DOC)

Table S7 miRNAs and their predicted target sites selected for experimental validation
(0.03 MB DOC)

Table S8 The 313 human miRNAs and 233 mouse miRNAs used as queries in this work (from miRBase release 7.0)
(0.07 MB DOC)

Table S9 Targets predicted for 313 human miRNAs with parameter: Watson-Crick matches at nucleotide positions 2–7 inclusive, <6 mismatches-and-GU-pairs at nucleotide positions ≥15, and 40% free energy threshold
(3.16 MB ZIP)

Table S10 Targets predicted for 313 human miRNAs with parameter: Watson-Crick matches at nucleotide positions 2–8, <6 mismatches-and-GU-pairs at nucleotide positions ≥15, and 40% free energy threshold
(2.02 MB ZIP)

Table S11 Targets predicted for 233 mouse miRNAs with parameter: Watson-Crick matches at nucleotide positions 2–7 inclusive, <6 mismatches-and-GU-pairs at nucleotide positions ≥15, and 40% free energy threshold
(2.26 MB ZIP)

Table S12 Targets predicted for 233 mouse miRNAs with parameter: Watson-Crick matches at nucleotide positions 2–8, <6 mismatches-and-GU-pairs at nucleotide positions ≥15, and 40% free energy threshold
(1.48 MB ZIP)

Table S13 The 181 orthologous human and mouse miRNAs that are identical in sequences at nucleotide positions 1–8
(0.04 MB DOC)
