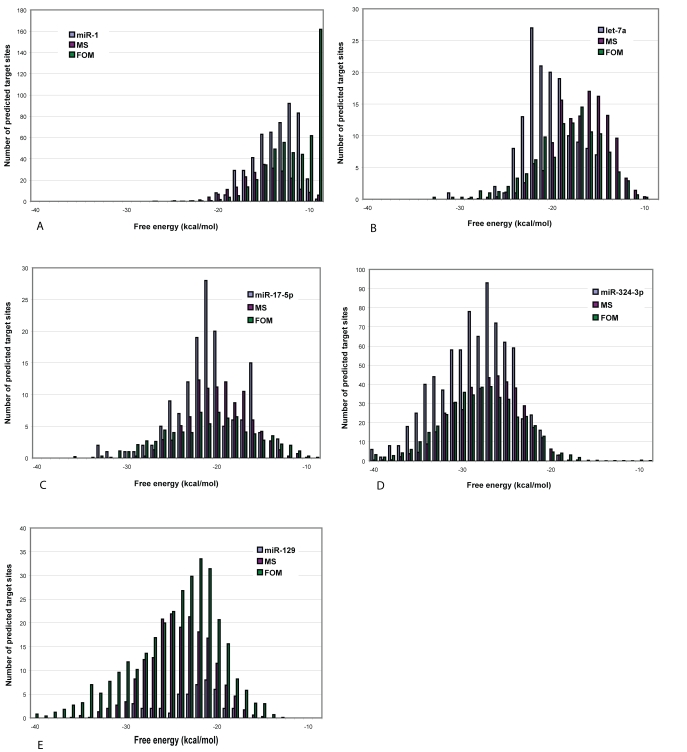# Correction: Transcriptome-Wide Prediction of miRNA Targets in Human and Mouse Using FASTH

**DOI:** 10.1371/annotation/e0842765-3cae-4737-8b5b-96aeb12d7fb5

**Published:** 2009-07-16

**Authors:** Chikako Ragan, Nicole Cloonan, Sean M. Grimmond, Michael Zuker, Mark A. Ragan

Figure 1 is a duplicate of Figure 8. Please view the correct Figure 1 here: 

**Figure:**