# Supplementary material for: Transcriptome-Wide Prediction of miRNA Targets in Human and Mouse Using FASTH
Source: PLoS One. 2009 May 29;4(5):e5745. doi: 10.1371/journal.pone.0005745 (PMC2684643; doi:10.1371/journal.pone.0005745)
Supplement: Supplementary Material S1 — Description of Supplementary Material (0.03 MB DOC) [file pone.0005745.s001.doc]

# Description of Supplementary Material.

## Supplementary Text

**Supplementary Table S1**

Number of predicted targets and signal-to-noise ratio with different filtering parameters for native miRNAs, mononucleotide shuffled (MS) and first-order Markov (FOM) control sequences.

**Supplementary Table S2**

Number of predicted targets and signal-to-noise ratio for 74 miRNAs, compared with control sequences from the Lewis *et al.* supplemental material.

## Supplementary Table S3

Numbers of nucleotides assigned to different mRNA regions (5′UTR, CDS, 3′UTR and intron), or gained or lost, among different isoforms of the same transcriptional region (gene).

## Supplementary Table S4

Degree of overlap between FASTH prediction sets and those of other methods.

## Supplementary Table S5

Degree of overlap among prediction sets of three methods.

**Supplementary Table S6**

Top ten over-represented Gene Ontology terms for Biological Process (BP), Cellular Component (CC) and Molecular Function (MF) among mRNAs predicted as miRNA targets.

## Supplementary Table S7

miRNAs and their predicted target sites selected for experimental validation.

## Supplementary Table S8

The 313 human miRNAs and 233 mouse miRNAs used as queries in this work (from miRBase release 7.0).

## Supplementary Table S9

Targets predicted for 313 human miRNAs with parameter: Watson-Crick matches at nucleotide positions 2-7 inclusive, <6 mismatches-and-GU-pairs at nucleotide positions ≥15, and 40% free energy threshold.

## Supplementary Table S10

Targets predicted for 313 human miRNAs with parameter: Watson-Crick matches at nucleotide positions 2-8, <6 mismatches-and-GU-pairs at nucleotide positions ≥15, and 40% free energy threshold.

## Supplementary Table S11

Targets predicted for 233 mouse miRNAs with parameter: Watson-Crick matches at nucleotide positions 2-7 inclusive, <6 mismatches-and-GU-pairs at nucleotide positions ≥15, and 40% free energy threshold.

## Supplementary Table S12

Targets predicted for 233 mouse miRNAs with parameter: Watson-Crick matches at nucleotide positions 2-8, <6 mismatches-and-GU-pairs at nucleotide positions ≥15, and 40% free energy threshold.

## Supplementary Table S13

The 181 orthologous human and mouse miRNAs that are identical in sequences at nucleotide positions 1-8.

## Supplementary Figure S1

Energetically favourable miRNA-mRNA hybrid secondary structure predicted (FASTH) for each target subjected to experimental validation in this study.
