# Supplementary material for: Transcriptome-Wide Prediction of miRNA Targets in Human and Mouse Using FASTH
Source: PLoS One. 2009 May 29;4(5):e5745. doi: 10.1371/journal.pone.0005745 (PMC2684643; doi:10.1371/journal.pone.0005745)
Supplement: Text S1 — Specification and generation of control sequences, and technical description of approach (0.08 MB DOC) [file pone.0005745.s002.doc]

**Supplementary Text**

Included in this supplementary text are:

**1** Specification and generation of control sequences

**2** Technical description of approach

**2.1** Stage 1: Energy-based prediction of optimally hybridizing regions using FASTH

**2.1.1** Pre-processing

**2.1.2** Matching a query to the database

**2.1.3** Calculation of free energy

**2.2** Stage 2: Identification of candidate miRNA binding sites by filtering the list of initial targets

###### **1. Specification and generation of control sequences**

As described above, our approach is based on minimizing the free energy of duplex structure. The hybridization energy at which a miRNA binds a target site is determined by the length of the duplex interaction region, the nucleotide composition within this region, and base-pair stacking. To estimate the false positive rate of prediction we calculate a S:N ratio, and for this calculation we generated two different sets of control sequences by randomization of known miRNAs.

The first set, intended for use in describing miRNA target-site specificity vis-à-vis unrelated background sequences, was generated by randomly permuting (shuffling) the nucleotides of each known miRNA [S1, S2]. Each is matched with the corresponding true miRNA in length and mononucleotide composition (count), but not in dinucleotide frequency or (except by chance) composition. The second set, intended for use in describing the one-to-one specificity of miRNA-mRNA binding, was generated *via* a first-order Markov process [S2] that preserves sequence length and, on average, dinucleotide frequency, but not (except by chance) mononucleotide composition (count). In both cases, ten randomized sequences were generated for each true miRNA. Following Workman & Krogh (1999) we refer to these sets as mononucleotide shuffled (MS) and first-order Markov (FOM) controls respectively.

miRNA-mRNA duplexes are stabilized by free energies arising from base-pairing and base-stacking. The former can be resolved into the contributions arising from the number of base pairs involved, and the mean free energy that each contributes. In both our MS and FOM control sets, the length of each random sequence is identical with that of the corresponding real miRNA. In generating the MS set we also controlled for mean energy contribution of each base pair by preserving mononucleotide compositions, thus expected proportions of strong (*e.g.* G-C), weaker (*e.g.* A-U) and mismatched base pairs. Again, our randomized sequences are imperfect controls in this regard, as it is computationally infeasible to ensure that mononucleotide composition is matched specifically within the regions of actual or potential intra-molecular hybridization. As miRNAs are short (19-25 nt), we believe it important to control explicitly for composition (count), not frequency (as would result from generation *via* a zero-order Markov model). Control of mononucleotide composition furthermore contributes, albeit not strongly, to the distribution of nearest-neighbor frequencies, hence to control of the free energy contribution from base-stacking.

Base-stacking energy arises from electronic interactions between bases at adjacent positions, and is thus primarily influenced by the nearest-neighbor (dinucleotide) frequency distribution, which we capture here *via* a first-order Markov process (the FOM sets). The mononucleotide composition is not preserved, although examination of a sample of the actual randomized sequences revealed that in most cases, nucleotide count differs by no more than one or two. Unlike the FOM approach, dinucleotide shuffling [S2] preserves dinucleotide (hence mononucleotide) composition, but for short sequences reduces the available state space to a perhaps unacceptable extent (unpublished).

###### Neither set of randomized sequences is a perfect control. A sequence in our MS set would be unlikely to find as many low-energy targets as the corresponding true miRNA due to the absence (except by chance) of stabilization by base-stacking. Our MS set will be “too random” in this context, and S:N ratios based thereupon will consequently be inflated. By contrast, the first-order Markov process generates sequences (our FOM sets) identical in dinucleotide frequency distribution to known miRNAs, which themselves may be anomalous vis-à-vis background. Except in mononucleotide count per se, our FOM sequences are thus as carefully matched as possible to real miRNAs, and thus are potentially much better-stabilized by base-stacking than even length- and mononucleotide composition-matched sequences from the random cellular background. S:N ratios based on the FOM-set ratios may correspondingly be unfairly depressed.

**2. Technical description of approach**

As this is the primary description of the FASTH algorithm and software, we present FASTH here in some detail.

**2.1.** Stage 1: Energy-based prediction of optimally hybridizing regions using FASTH

The FASTH program (FAST Hybridization) was created to provide a flexible, scalable alternative to the use of programs such as BLAST [S3] when searching for potential targets of oligonucleotides in large sequence databases. It can be used for DNA as well as for RNA. FASTH employs a search strategy similar to that of FASTA [S4], allowing genome-scale databases to be searched efficiently.

The execution time of FASTH scales linearly in number of queries, sub-linearly in database size, and linearly in query length. OnAMD64 hardware (1 GB RAM) in a Linux cluster, searching a database of 500 mRNAs (~1.5M bp) and recovering the 2000 energetically most-favourable miRNA target-site predictions in each requires 4 seconds per miRNA using the FASTH default parameters,. This time includes 1 second to re-calculate energies using hybrid-min in the UNAFold package [S5] (see below), but does not include the initial reading phase in which pre-processed database files are loaded. RNAhybrid (version 2.1) under default parameters takes 125 seconds per miRNA to search the same set of mRNAs, recovering only the top 3 predicted target sites in each.

In this first stage, putative match sites (*targets*) of oligonucleotides (*queries*) are selected and ranked using binding free energy as the criterion. Following two pre-processing steps, the complement of the query sequence is located in the database, and the MFE is computed, first approximately, then rigorously using the program hybrid-min in the UNAFold package [S5], taking into account base-pair and base-pair-stacking free energies in perfect helices, unfavorable energy contributions from interior loops and bulges, favorable contributions from single-stranded bases or mismatched pairs adjacent to a base pair, and initiation energy.

##### 2.1.1. Pre-processing

Before FASTH can be run on a collection of queries, two preprocessing steps are required. Any nucleic acid database in FASTA format can be used. The letters A, C, G, T, U and N are allowed, in uppercase or lowercase. The letters T and U are considered to be equivalent. The free energy tables that are read by FASTH treat T as U when RNA free energies are used, and U as T when DNA parameters are used. The letter N is treated as neutral. It does not pair, but an X·Y mismatch is not penalized if either X or Y (or both) are N. The use of any other character in the database or in query sequences will generate an error. In addition, FASTH searches only the “sense-strand” that it is given.

The Preprocessing program takes a FASTA-formatted database and turns it into a long string of characters with no spaces or line feed characters. Every title line in the database is processed into a string that begins with the character # and terminates with the character $. For this reason, the characters # and $ are not permitted to appear in the FASTA-formatted database. The beginning > characters are removed. The sequence information that appears in subsequent lines is turned into a continuous string of characters, with all blank spaces and line feed characters removed. It is concatenated to the title and begins after the $. The # character indicates the beginning of the next sequence. For example, the simple file

>M poly

ggaUgCggUCaUa

>P trich

CgUgCUgCaUCCa

>L het

gaUgUCaUaCCaN

would be converted to

#M poly$GGAUGCGGUCAUA#P trich$CGUGCUGCAUCCA#L het$GAUGUCAUACCAN

Note that spaces in titles are preserved, but are eliminated in the sequences, and that all sequence characters have been converted to uppercase. The suffix “.database” is appended to the name of the database, and the output is stored in a file with this name.

The second preprocessing step is the separation of titles and sequences into two files and the

creation of a hash table. This is accomplished by the Data_Hash program. A word size (WS) is selected and Data_Hash chops the entire database into all words of length WS (WS-mers). A hash of the 15-mer 5′-ACCGUUACGACUUAC-3′ into all words of size 2 yields 14 2-mers, labeled consecutively from the 5′ end beginning with zero: 0 AC, 1 CC, 2 CG, 3 GU, 4 UU, 5 UA, 6 AC, 7 CG, 8 GA, 9 AC, 10 CU, 11 UU, 12 UA and 13 AC. A hash table stores the positions in the sequence where each word occurs. This list is sorted lexicographically (as in a dictionary) by 2-words. Although a word can occur more than once in the database, it will appear only once in the hash table. For this simple example, the hash table is as follows:

| AC | 0, 6, 9, 13 | 0, 6, 3, 4 |
| --- | --- | --- |
| CC | 1 | 1 |
| CG | 2, 7 | 2, 5 |
| CU | 10 | 10 |
| GA | 8 | 8 |
| GU | 3 | 3 |
| UA | 5, 12 | 5, 7 |
| UU | 4, 11 | 4, 7 |

**A hash table of 2-mers for the 15-mer ACCGUUACGACUUAC**

Column one contains each distinct word (2-mer), column two contains the position(s) in the database (sequence) at which each word is found, and column three compresses the information in the previous column by storing the relative positions of each occurrence of a word after the first. In a human chromosome, the numbers in column 2 may be 9 digits long, whereas those in column 3 would be much smaller on average.

The hash table immediately below, for the 15-mer 5′-ACCGUUACGACUUAC-3′, is derived using a two-letter alphabet, where R stands for puRine and Y for pYrimidine:

| RR | 8 | 8 |
| --- | --- | --- |
| RY | 0, 3, 6, 9, 13 | 0, 3, 3, 3, 4 |
| YR | 2, 5, 7, 12 | 2, 3, 2, 5 |
| YY | 1, 4, 10, 11 | 1, 3, 6, 1 |

These two tables are equivalent if a two-letter R,Y alphabet is used in place of A, C, G and U.

Running Data_Hash on the three-sequence database above yields three files. If the name of the database file is Example.database, a *lookup file*, a *concatenated sequence file* and a *hash data file* are created.

Example concat_seq: in this case, it is

$$$$$GGAUGCGGUCAUA$$$$$CGUGCUGCAUCCA$$$$$GAUGUCAUACCAN

The sequence labels have been removed and a string of dollar signs separates one sequence from the other. The number of these sequence separators is chosen by the user and ensures that a hit (putative target) in one sequence will not overlap with a hit in an adjacent sequence.

Example lookup_file: this file stores the sequence names and links each sequence to a position in the database. There is one entry for each sequence. The entry for sequence 2, “P trich”, is

#s

2

Start Position: 23

End Position: 35

Length: 13

NAME: P trich

#e

Using these files, FASTH can easily translate “position 9 in P trich” to position 32 in the concatenated sequence file (23 + 9 = 32). Translating the other way is also straightforward, because the Start Positions are in ascending order in this file.

Example hash_data: only four two-letter words are possible in this simple case. Each word is referred to as a KEY. The hash table is:

KEY 1 , number of occurrences: 4 , KEY string is RR

$

5 1 5 30

*

KEY 2 , number of occurrences: 13 , KEY string is YR

$

8 2 4 2 7 2 3 2 4 9 3 2 3

*

KEY 3 , number of occurrences: 12 , KEY string is RY

$

7 2 3 3 9 2 3 2 11 2 3 2

*

KEY 4 , number of occurrences: 6 , KEY string is YY

$

13 14 5 1 12 5

*

FASTH compares words from the reverse complement of each query to words in the database. Exact matches are tagged. If a four-letter alphabet were used, then only Watson-Crick base pairs could be detected in exact matches. G·U wobble pairs would not be found since they would be treated as mismatches. The result is a loss of sensitivity that FASTH seeks to avoid. Using a two-letter R,Y alphabet, searching for exact matches between words in the database and words in the reverse complement of query sequences yields duplexes that contain Watson-Crick, G·U and A·C base pairs. The G·U base pairs are desirable; all word matches containing A·C base pairs are discarded. This can be done because FASTH has access to the original databases as well as to the hash table. The example below, a comparison of a database 8-mer (5′-CGAGUAUU-3′) with three different query words, illustrates how FASTH detects complementary regions:

| Two-letter alphabet Four-letter alphabet |
| --- |
| 5′-YRRRYRYY-3′ 5′-CGAGUAUU-3′  A 5′-YRRRYRYY-3′ 5′-CGAGUAUU-3′ Accept  3′-RYYYRYRR-5′ 3′-GCUCAUAA-5′ |
| 5′-YRRRYRYY-3′ 5′-CGAGUAUU-3′  B 5′-YRRRYRYY-3′ 5′-CGAAUAUU-3′ Accept with G·U pair  3′-RYYYRYRR-5′ 3′-GCUUAUAA-5′ |
| 5′-YRRRYRYY-3′ 5′-CGAGUAUU-3′  C 5′-YRRRYRYY-3′ 5′-CGAGUGUU-3′ Reject! A·C pair  3′-RYYYRYRR-5′ 3′-GCUCACAA-5′ |

In each block A, B and C, column two contains the database word, the complement of a query word and the query word itself (reversed) in rows one, two and three respectively. The two-letter R,Y alphabet is used. Column three contains the identical information, but uses the standard four-letter alphabet. In each of the three blocks, rows one and two of column two are identical, indicating a perfect match. Rows one and three contain Y·R or R·Y “base pairs”. All three queries are initially tagged as perfect matches (perfect hybridization). In block A, the query is 5′-AAUACUCG-3′. Rows one and two in column three are identical, indicating perfect WC pairing; this match is accepted. In block B, rows one and two are non-identical, but the mismatch is a G·U base pair, so the match is accepted. The example in Block C is similar to B, except that an A·C mismatch is detected, so the mismatch is rejected.

##### 2.1.2 Matching a query to the database

Preprocessing is required once only for a given database and word size. FASTH may be run multiple times with files containing multiple queries. Given a query, FASTH computes all words in the reverse complement. In the two-letter R,Y alphabet, every exact match in the database of these words is quickly tabulated. If *w* is the word size, then a perfect match means that there is an index, *j*, in the database and an index, *i*, in the query, such that bases *j*, *j* + 1, …, *j* + *w* -1 in the database are potentially complementary to bases *i*, *i* - 1, …, *i* - w + 1 in the query, respectively. The word *potentially* is used because these matches may contain forbidden base pairs, as explained above. The match is said to occur in diagonal *i* + *j*. This is because base *i* + *k* in the query pairs with base *j* - *k* in the database, for *k* = 0, 1, …, *w* -1. That is, the sum of the indices of paired bases is constant. This is visually represented in the figure below, where the row index *i* corresponds to query numbering 5′ to 3′ (top to bottom), and the where the column index *j* corresponds to database numbering 5′ to 3′ (left to right). FASTH flags those diagonals where at least *c* matches occur, where *c* (cutoff) and is set by the user. All other positions in the database are ignored. It is this initial filtering procedure that enables FASTH to search a large database in reasonable time.

**2.1.3** Calculation of free energy

For every diagonal *d* that is flagged, FASTH considers hybridization on neighboring diagonals *d* - *b*, *d* – *b* + 1, …, *d*, *d* + 1, …, *d* + *f* , where *b* is the “backward search parameter” and *f* is the “forward search parameter”. Both are set by the user. A primitive *ad hoc* procedure is used to determine a crude initial hybridization and a corresponding free energy. The word matches on diagonal *d* are merged if they overlap and all of these base pairs are retained. Any base pairs in matches found in the adjacent diagonals are added to the hybridization if they do not conflict with existing base pairs. The addition of these extra base pairs introduces small bulges or interior loops (mismatches). The score of the resulting hybridization is the sum of the hybridization free energies of the resulting helices. That is, base-pair and base-pair-stacking energies are considered, but loop and mismatch effects are ignored. The stacking free energies used for the miRNA searches are those used by MFOLD [S6].

FASTH stores all of these initial free energies, denoted by Sort dG. All of the initial hybridizations are sorted using the Sort dG values, and the top *r* results are placed in a file named score_sort.txt, where *r* is chosen by the user. Alternatively, the user is allowed to select a percent *p*, in which case the top *p*% of the hybridizations are placed in the score_sort.txt file. (The results for each query are placed in individual subdirectories, enabling the use of common names for the output files.) Another computed file, score_array.txt, contains a pair of numbers (n*_i_* , e*_i_*) in line *i*, where *n_i_* is the number of hybridizations that have initial free energies equal to *e_i_*. These free energies increase by 1 kcal/mol, starting with the minimum Sort dG on line 1 and ending with the highest initial free energy on the last line. A search of the negative strand of *Homo sapiens* chromosome 17, using mir-196a as a query, was performed (note that mir-196a targets an mRNA in a Hox B gene cluster found on chromosome 17 [S7, S8]). The parameter values used were: *w* = 7, *c* = 2, *f* = 2 and *b* = 2. Only the first two affect the number of flagged diagonals. This chromosome (May 2004 assembly) has 78774742 bp. The number of flagged diagonals was 948955, meaning that only 1.2% of the diagonals were retained for further analysis.

The top-scoring results are subsequently re-analyzed using the rigorous, free-energy-minimizing program hybrid-min [S5]. That is, MFE hybridizations are computed for each putative target, expanded by *b* upstream and by *f* downstream, *versus* the query. This results in new hybridizations and free energies. These free energies are labeled Min dG and allow the top results to be reordered. Other quantities are computed as well. The match decrement *Mdec* is the number of bases of the query that are not paired. The match deficiency *Def* is Mdec plus the number of target bases in bulges or interior loops. All of these results are placed in tab-delimited files that allow for easy importation to spreadsheets, or for sorting by a variety of other criteria.

Visually representing the best initial hybridization between a simple “database” (29 nt) and query, using *w* = 5, *c* = 2, *b* = *f* = 3, the initial raw result is

Diag#:33, Sort dG = -34.50, Hit 1

Db seq: database, From 1-29

CGGACGCACACCGGGGAGAAGCGCUUCCC

uguguguuuucccuucgugaaaa

uguguguuuucccuucgugaaaa

uguguguuuucccuucgugaaaa

UGUGUGUuuUCCCUUcgugaaaa

uguguguuuucccuucgugaaaa

uguguguuuucccuuCGUGAAaa

uguguguuuucccuucgugaaaa

The database sequence is written 5′ to 3′ (left to right) in uppercase, third row down. The subsequent 7 (3 + 1 + 3) lines contain the query sequence, written 3′ to 5′ (left to right). Only those bases that pair in the initial hybridization are written using uppercase letters. The indentation assures that an uppercase query nucleotide can pair with the database nucleotide above it in the same column. The flagged diagonal corresponds to the fourth copy of the reversed query. A total of 5 exact matches were found for this diagonal. They were merged into two larger matches of sizes 7 and 6. The second forward diagonal contributes another six matches. This implies that a bulge of size two must occur in database sequence. The initial hybridization is thus

Sort dG = -29.9

CGG CC AA CCC

ACGCACA GGGGAG GCGCUU

UGUGUGU UCCCUU CGUGAA

--- UU -- AA-

In this example, Mdec = 4 and Def = 8. The revised hybridization, together with a higher (worse) free energy that now counts unfavorable loop effects, is

Min dG = -24.4

CGG CC A CCC

ACGCACA GGGG GAAGCGCUU

UGUGUGU UUCC CUUCGUGAA

--- U- - AA-

Note that the true free energy of the initial hybridization is -24.4 kcal/mol. For the MFE hybridization, Mdec = 3 and Def = 6.

**2.2** Stage 2: Identification of candidate miRNA binding sites by filtering the list of initial targets

In the second stage of our approach, we refine the first-stage predictions by applying further criteria *e.g.* based on biologically motivated information about externally validated miRNA target sites, for example constraints on length of the seed region, number of non-WC base pairs involving the 3′ end of miRNAs, and relative free energy score. As our approach to experimental validation (below) could not readily be extended to targets in *Alu* elements, at this stage we discarded those first-stage predictions identified by RepeatMasker version open-3.0.8 (www.repeatmasker.org) as corresponding to *Alu* regions in mRNAs; this should not be interpreted as disallowing the possibility that miRNAs may target the expression of genes located in *Alu* elements [S9]. Where for a given miRNA-mRNA pair we predict multiple target sites having seed regions offset by ≤6 nt, we consider only the lowest-energy interaction [S10]. In more detail:

*Base-pairing in the seed region*, defined alternatively as nt positions 2-7 or 2-8 from the 5′ end of the miRNA. The seed region is defined as a region of perfect or near-perfect WC matches, so we allowed either perfect WC base-pairing, or alternatively only a single GU pair.

*Base-pairing in the 3′ end of miRNA*, defined as beginning at nt position 15. Many targets have been validated where complementarity between miRNA and the mRNA target is sub-optimal in this region. To focus our search on the more energetically favorable candidates, we sometimes imposed WC base-pairing criteria in this region. *Mismatches* include unpaired bases (including energetically unfavorable canonical pairs, where forcing a base pair results in a higher MFE) and bulges.

*Free energy score* relative to the perfect WC reverse complement duplex with mRNA over the entire length of each miRNA, sometimes required to be ≥40% of the best score. For example, if the best energy score of a particular miRNA is -40 kcal/mol, we sometimes selected only potential targets having free energy below -40 × 0.4 = -16 kcal/mol.

The five filtering conditions we applied (Results and Fig. 3) derive from combinations of these criteria. For each miRNA, potential targets were then ranked by MFE score. Although many miRNAs can bind to more than one mRNA, and some mRNAs are known to be targeted by multiple miRNAs, here we treat interactions as if they involved only a single miRNA and a single mRNA; *i.e.* we did not adjust scores to reflect multiple matches. Further filtering could in principle be based on transcriptional profiles of miRNAs and/or target mRNAs, reflecting the assumption that regulation can occur only if both miRNA and mRNA are expressed simultaneously in the same cell and subcellular compartment (spatio-temporal domain); however, few relevant data are available.

**References to Supplementary Text**

S1. Altschul SF, Erickson BW (1985) Significance of nucleotide sequence alignments: a method for random sequence permutation that preserves dinucleotide and codon usage. Mol Biol Evol 2: 526-38.

S2. Workman C, Krogh A (1999) No evidence that mRNAs have lower folding free energies than random sequences with the same dinucleotide distribution. Nucl Acids Res 27: 4816-22.

S3. Altschul SF, Gish W, Miller W, Myers EW, Lipman DJ (1990) Basic local alignment search tool. J Mol Biol 215: 403-10.

S4. Pearson WR, Lipman DJ. (1988) Improved tools for biological sequence comparison. Proc Natl Acad Sci U S A 85: 2444-2448.

S5. Markham NR, Zuker M (2008) UNAFold: software for nucleic acid folding and hybridization. Methods Mol Biol 453:3-31.

# S6. Zuker M (2003) Mfold web server for nucleic acid folding and hybridization prediction. **Nucl Acids Res** 31: 3406-3415.

S7. Manfield JH, Harf BD, Nissen R, Obenauer J, Srineel J, *et al.* (2004) MicroRNA-responsive ‘sensor’ transgenes uncover Hox-like and other developmentally regulated patterns of vertebrate microRNA expression. Nat Genet 36: 1079-83.

S8. Yekta S, Shih, I, Bartel DP (2004) MicroRNA-directed cleavage of HOXB8 mRNA. Science 304: 594-596.

# S9. Smalheiser NR, Torvik VI (2006) Alu elements within human mRNAs are probable microRNAs targets. Trends Genet 22: 532-536.

S10. Doench JG, Sharp PA (2004) Specificity of microRNA target selection in translational repression. Genes Dev 18: 504-511.
