# Supplementary material for: Transcriptome-Wide Prediction of miRNA Targets in Human and Mouse Using FASTH
Source: PLoS One. 2009 May 29;4(5):e5745. doi: 10.1371/journal.pone.0005745 (PMC2684643; doi:10.1371/journal.pone.0005745)
Supplement: Figure S1 — Energetically favourable miRNA-mRNA hybrid secondary structure predicted (FASTH) for each target subjected to experimental validation in this study (0.03 MB DOC) [file pone.0005745.s003.doc]

Supplementary Figure S1.

Energetically favourable miRNA-mRNA hybrid secondary structure predicted (FASTH) for each target subjected to experimental validation in this study.

* hsa-miR-17-5p / TNFSF12 interaction is predicted in two different mRNAs.

hsa-miR-15a

mRNA_ID: NM_022117 UTR: 3 Gene_Symbol: TSPYL2

Query_Seq: UAGCAGCACAUAAUGGUUUGUG

Target_Sequence: CCCCGCAGGCUUCUGUGUGCUGCUAACU

Min_dG: -29.6

CCC UC ACU

CGCAGGCU UGUGUGCUGCUA

GUGUUUGG AUACACGACGAU

UA ---

hsa-miR-15a

mRNA_ID: NM_000633 UTR: 3 Gene_Symbol: BCL2

Query_Seq: UAGCAGCACAUAAUGGUUUGUG

Target_Sequence: UGGAAUAUCCAAUCCUGUGCUGCUAUCC

Min_dG: -19.0

UGGA U- AUCC UCC

AUA CCA UGUGCUGCUA

UGU GGU ACACGACGAU

G UU AAU- ---

hsa-miR-17-5p*

mRNA_ID: NM_003809 UTR: CDS Gene_Symbol: TNFSF12

Query_Seq: CAAAGUGCUUACAGUGCAGGUAGU

Target_Sequence: CUACUACCUGUACUGUCAGGUGCACUUUGA

Min_dG: -34.1

CU CAGGU A

ACUACCUGUACUGU GCACUUUG

UGAUGGACGUGACA CGUGAAAC

UU--- -

hsa-miR-17-5p*

mRNA_ID: NM_153012 UTR: 3 Gene_Symbol: TNFSF12

Query_Seq: CAAAGUGCUUACAGUGCAGGUAGU

Target_Sequence: CUACUACCUGUACUGUCAGGUGCACUUUGA

Min_dG: -34.1

CU CAGGU A

ACUACCUGUACUGU GCACUUUG

UGAUGGACGUGACA CGUGAAAC

UU--- -

hsa-miR-324-3p

mRNA_ID: NM_004422 UTR: CDS Gene_Symbol: DVL2

Query_Seq: CCACUGCCCCAGGUGCUGCUGG

Target_Sequence: GGGUAGCAGCACUGGGGGCGGUGGGGUU

Min_dG: -41.5

GGG G GGUU

UAGCAGCACU GGGGCGGUGG

GUCGUCGUGG CCCCGUCACC

G A ----

hsa-miR-324-3p

mRNA_ID: NM_004380 UTR: CDS Gene_Symbol: CREBBP

Query_Seq: CCACUGCCCCAGGUGCUGCUGG

Target_Sequence: GCCCAGCAUGCCUCCCGGGCAGUGGCAG

Min_dG: -33.2

GC - CCC CAG

CCAGCA UGCCU GGGCAGUGG

GGUCGU GUGGA CCCGUCACC

C C-- ---

hsa-miR-324-3p

mRNA_ID: NM_003396 UTR: CDS Gene_Symbol: WNT9B

Query_Seq: CCACUGCCCCAGGUGCUGCUGG

Target_Sequence: GAGAGCCGGCAGGCCUGGCAGUGGGGCG

Min_dG: -32.6

GAG - -- GGCG

CCGGCAG GCCU GGCAGUGG

GGUCGUC UGGA CCGUCACC

G CC ----
