# Supplementary material for: Transcriptome-Wide Prediction of miRNA Targets in Human and Mouse Using FASTH
Source: PLoS One. 2009 May 29;4(5):e5745. doi: 10.1371/journal.pone.0005745 (PMC2684643; doi:10.1371/journal.pone.0005745)
Supplement: Table S1 — Number of predicted targets and signal-to-noise ratio with different filtering parameters for native miRNAs, mononucleotide shuffled (MS) and first-order Markov (FOM) control sequences (0.06 MB DOC) [file pone.0005745.s004.doc]

**Supplementary Table S1.** Number of predicted targets and signal-to-noise ratio with different filtering parameters for native miRNAs, mononucleotide shuffled (MS) and first-order Markov (FOM) control sequences. Human results are based on 313 miRNAs, mouse results on 230 miRNAs. Defining conditions are:

**A.** WC base pairs within nt 2-7 only

**B.** WC base pairs within nt 2-8 only

**C.** WC base pairs within nt 2-7 only + 40% FE threshold

**D.** WC base pairs within nt 2-8 only + 40% FE threshold

**E.** WC base pairs within nt 2-7 and < 6 mismatches and GU pairs at nt ≥ 15

**F.** WC base pairs within nt 2-8 and < 6 mismatches and GU pairs at nt ≥ 15

**G.** WC base pairs within nt 2-7, < 6 mismatches and GU pairs at nt ≥ 15, and 40% FE threshold

**H.** WC base pairs within nt 2-8, < 6 mismatches and GU pairs at nt ≥ 15, and 40% FE threshold

**I.** WC base pairs with ≤ 1 GU pair within nt 2-7, < 6 mismatches and GU pairs at nt ≥ 15, and 40% FE threshold

**J.** WC base pairs with ≤ 1 GU pair within nt 2-8, < 6 mismatches and GU pairs at nt ≥ 15, and 40% threshold

**K.** WC base pairs within nt 2-8, ≤ 1 loop within nt 9-14, < 6 mismatches and GU pairs at nt ≥ 15, and 40% FE threshold

**A-Rev.** Parameter A applied from the 3’ end of miRNA

**B-Rev.** Parameter B applied from the 3’ end of miRNA

**G-Rev.** Parameter G applied from the 3’ end of miRNA

**H-Rev.** Parameter H applied from the 3’ end of miRNA

| **Transcriptome** | **Condition** | **Number of targets miRNA** | **Number of targets MS** | **S:N ratio**  **MS** | **Number of targets FOM** | **S:N ratio**  **FOM** |
| --- | --- | --- | --- | --- | --- | --- |
| Human | A | 397673 | 297535 | 1.34 | 376635 | 1.06 |
|  | B | 256092 | 172613 | 1.48 | 222847 | 1.15 |
|  | C | 316967 | 216725 | 1.46 | 274056 | 1.16 |
|  | D | 207738 | 128924 | 1.61 | 168745 | 1.23 |
|  | E | 201366 | 133356 | 1.51 | 171060 | 1.18 |
|  | F | 125223 | 71280 | 1.76 | 97132 | 1.29 |
|  | G | 175207 | 107377 | 1.63 | 142285 | 1.23 |
|  | H | 111981 | 59933 | 1.87 | 84396 | 1.33 |
|  | I | 423963 | 322722 | 1.31 | 389153 | 1.09 |
|  | J | 282231 | 203229 | 1.39 | 250059 | 1.13 |
|  | K | 89416 | 48672 | 1.84 | 71755 | 1.25 |
|  | A-Rev | 315288 | 302340 | 1.04 | 377207 | 0.84 |
|  | B-Rev | 203553 | 178668 | 1.14 | 243385 | 0.84 |
|  | G-Rev | 126886 | 104694 | 1.21 | 153111 | 0.83 |
|  | H-Rev | 73474 | 61200 | 1.20 | 93447 | 0.79 |
|  | | | | | | |
| Mouse | A | 287040 | 210829 | 1.36 | 257018 | 1.12 |
|  | B | 187673 | 120245 | 1.56 | 161538 | 1.16 |
|  | G | 114035 | 67910 | 1.68 | 89651 | 1.27 |
|  | H | 74455 | 38375 | 1.94 | 55484 | 1.34 |
