# Supplementary material for: Transcriptome-Wide Prediction of miRNA Targets in Human and Mouse Using FASTH
Source: PLoS One. 2009 May 29;4(5):e5745. doi: 10.1371/journal.pone.0005745 (PMC2684643; doi:10.1371/journal.pone.0005745)
Supplement: Table S2 — Number of predicted targets and signal-to-noise ratio for 74 miRNAs, compared with control sequences from the Lewis et al. supplemental material (0.04 MB DOC) [file pone.0005745.s005.doc]

**Supplementary Table S2.** Number of predicted targets and signal-to-noise ratio for 74 miRNAs, compared with control sequences from the Lewis *et al.* supplemental material. Conditions as in Supplementary Table S1.

| **Transcriptome** | **Condition** | **Number of targets miRNA** | **Number of targets shuffled** | **S:N ratio** |
| --- | --- | --- | --- | --- |
| Human | A | 112797 | 97907 | 1.15 |
|  | B | 76953 | 67599 | 1.14 |
|  | C | 82913 | 71215 | 1.16 |
|  | D | 58109 | 50967 | 1.14 |
|  | E | 55762 | 40974 | 1.36 |
|  | F | 35879 | 25898 | 1.39 |
|  | G | 46469 | 32306 | 1.44 |
|  | H | 31519 | 21680 | 1.45 |
|  | A - Rev | 75814 | 80773 | 0.94 |
|  | B - Rev | 49155 | 43594 | 1.13 |
|  | G - Rev | 24943 | 27595 | 0.90 |
|  | H - Rev | 20108 | 16835 | 1.19 |
|  | | | | |
| Mouse | A | 101058 | 91892 | 1.10 |
|  | B | 68035 | 62426 | 1.09 |
|  | G | 41207 | 28608 | 1.44 |
|  | H | 27342 | 19012 | 1.44 |

*Reference*

Lewis BP, Burge C, Bartel DP (2005) Conserved seed pairing, often flanked by adenosines, indicates that thousands of human genes are microRNAs targets. Cell 120: 15-20.
