# Supplementary material for: Transcriptome-Wide Prediction of miRNA Targets in Human and Mouse Using FASTH
Source: PLoS One. 2009 May 29;4(5):e5745. doi: 10.1371/journal.pone.0005745 (PMC2684643; doi:10.1371/journal.pone.0005745)
Supplement: Table S3 — Numbers of nucleotides assigned to different mRNA regions (5′UTR, CDS, 3′UTR and intron), or gained or lost, among different isoforms of the same transcriptional region (gene) (0.12 MB DOC) [file pone.0005745.s006.doc]

**Supplementary Table S3.** Numbers of nucleotides assigned to different mRNA regions (5′UTR, CDS, 3′UTR and intron), or gained or lost, among different isoforms of the same transcriptional region (gene). Numbers in the shorter-length isoforms are shown in columns, and numbers in the longer isoforms are shown in rows. Comparison sets: **A, C and D**, genes with exactly 2 isoforms (1947 genes, 3894 mRNAs, 1947 comparisons); **B**, **E** and **F**, genes with ≥ 3 isoforms (range 3 – 23; 996 genes 4071 mRNAs, 8251 comparisons). N/A, not applicable.

| **Set** | **Overlap exclude at edge of CDS (# nt)** | **Number of nucleotides…** | **In introns** | **In 5′UTRs** | **In CDSs** | **In 3′UTRs** | **In mRNAs without gain/loss** | **Reassigned to different regions** | **Gained or lost in different isoforms** | **Number of nucleotides in mRNAs** |
| --- | --- | --- | --- | --- | --- | --- | --- | --- | --- | --- |
| A | None | In introns | (138234542) | 115118 | 174373 | 269279 | N/A | N/A | 558770  (9.8%)** | N/A |
|  | None | In 5′UTRs | 125249 | 280899 | 18911 | 25 | 299835 | 18936 | N/A | 425084 |
|  | None | In CDSs | 493109 | 21019 | 3032632 | 47923 | 3101574 | 68942 |  | 3594683 |
|  | None | In 3′UTRs | 546013 | 1435 | 82295 | 1634717 | 1718447 | 83730 |  | 2264460 |
|  | None | In mRNAs w/o gain/loss | N/A | 303353 | 3133838 | 1682665 | 5119856 | N/A | N/A | N/A |
|  | None | Reassigned to diff. regions | N/A | 22454 | 101206 | 47948 | N/A | 171608  (3.4%)* | N/A | N/A |
|  | None | Gained/lost in diff. isoforms | 1164371  (18.5%)** | N/A | | | N/A | N/A | N/A | 6284227 |
|  | None | In mRNAs in total | N/A | 418471 | 3308211 | 1951944 | N/A | N/A | 5678626 |  |
|  | | | | | | | | | | |
| B | None | In introns | (962270741) | 278786 | 407680 | 574610 | N/A | N/A | 1261076  (4.7%)** | N/A |
|  | None | In 5′UTRs | 367913 | 1842894 | 132744 | 16289 | 1991927 | 149033 | N/A | 2359840 |
|  | None | In CDSs | 1538017 | 1051390 | 12727860 | 828891 | 14608141 | 1880281 |  | 16146158 |
|  | None | In 3′UTRs | 982297 | 21563 | 648811 | 8124443 | 8794817 | 670374 |  | 9777114 |
|  | None | In mRNAs w/o gain/loss | N/A | 2915847 | 13509415 | 8969623 | 25394885 | N/A | N/A | N/A |
|  | None | Reassigned to diff. regions | N/A | 1072953 | 781555 | 845180 | 25394885 | 2699688  (10.6%)* | N/A | N/A |
|  | None | Gained/lost in diff. isoforms | 2888227  (10.2 %)** | N/A | | | N/A | 28283112 | N/A |  |
|  | None | In mRNAs in total | N/A | 3194633 | 13917095 | 9544233 | N/A | N/A | 26655961 |  |
|  | | | | | | | | | | |
| C | 5 | In introns | N/A | N/A | N/A | N/A | N/A | N/A | N/A | N/A |
|  | 5 | In 5′UTRs | 1033 | 7976 | 711 | 15 | 8702 | 726 | N/A | 9735 |
|  | 5 | In CDSs | 3549 | 415 | 18190 | 1210 | 19815 | 1625 |  | 23364 |
|  | 5 | In 3′UTRs | 1721 | 5 | 376 | 7633 | 8014 | 381 |  | 9735 |
|  | 5 | In mRNAs w/o gain/loss | N/A | 8396 | 19277 | 8858 | 36531 | N/A | N/A | N/A |
|  | 5 | Reassigned to diff. regions | N/A | 420 | 1087 | 1225 | N/A | 2732  (7.5%)* | N/A | N/A |
|  | 5 | Gained/lost in diff. isoforms | 6303  (14.7%)** | N/A | | | N/A | 42834 | N/A |  |
|  | 5 | In mRNAs in total | N/A | 8396 | 19277 | 8858 | N/A | N/A | 36531 |  |
|  | | | | | | | | | | |
| D | 10 | In introns | N/A | N/A | N/A | N/A | N/A | N/A | N/A | N/A |
|  | 10 | In 5′UTRs | 2235 | 15863 | 1342 | 30 | 17235 | 1372 | N/A | 19470 |
|  | 10 | In CDSs | 6328 | 748 | 33553 | 2205 | 36506 | 2953 |  | 42834 |
|  | 10 | In 3′UTRs | 3554 | 10 | 741 | 15165 | 15916 | 751 |  | 19470 |
|  | 10 | In mRNAs w/o gain/loss | N/A | 16621 | 35636 | 17400 | 69657 | N/A | N/A | N/A |
|  | 10 | Reassigned to diff. regions | N/A | 758 | 2083 | 2235 | N/A | 5076  (7.3%)* | N/A | N/A |
|  | 10 | Gained/lost in diff. isoforms | 12117  (14.8%)** | N/A | | | N/A | 81774 | N/A |  |
|  | 10 | In mRNAs in total | N/A | 16621 | 35636 | 17400 | N/A | N/A | 69657 |  |
|  | | | | | | | | | | |
| E | 5 | N/A | N/A | N/A | N/A | N/A | N/A | N/A | N/A | N/A |
|  | 5 | 3739 | 34047 | 3379 | 90 | 37516 | 3469 | N/A | 41255 | 3739 |
|  | 5 | 9568 | 2020 | 79922 | 7502 | 89444 | 9522 |  | 99012 | 9568 |
|  | 5 | 4039 | 92 | 2183 | 34941 | 37216 | 2275 |  | 41255 | 4039 |
|  | 5 | N/A | 36159 | 85484 | 42533 | 164176 | N/A | N/A | N/A | N/A |
|  | 5 | N/A | 2112 | 5562 | 7592 | N/A | 15266  (9.3%)* | N/A | N/A | N/A |
|  | 5 | 17346  (9.6%)** | N/A | N/A | N/A | N/A | 181522 | N/A | N/A | 17346  (9.6%)** |
|  | 5 | N/A | 36159 | 85484 | 42533 | N/A | N/A | 164176 |  | N/A |
|  | | | | | | | | | | |
| F | 10 | N/A | N/A | N/A | N/A | N/A | N/A | N/A | N/A | N/A |
|  | 10 | 7439 | 68378 | 6552 | 141 | 75071 | 6693 | N/A | 82510 | 7439 |
|  | 10 | 16857 | 3602 | 147485 | 13578 | 164665 | 17180 |  | 181522 | 16857 |
|  | 10 | 8047 | 172 | 4306 | 69985 | 74463 | 4478 |  | 82510 | 8047 |
|  | 10 | N/A | 72152 | 158343 | 83704 | 314199 | N/A | N/A | N/A | N/A |
|  | 10 | N/A | 3774 | 10858 | 13719 | N/A | 28351  (9.0%)* | N/A | N/A | N/A |
|  | 10 | 32343  (9.3%)** | N/A | N/A | N/A | N/A | 346542 | N/A | N/A | 32343  (9.3%)** |
|  | 10 | N/A | 72152 | 158343 | 83704 | N/A | N/A | 314199 |  | N/A |

***Notes:***

* The proportion of nucleotides assigned into different regions of mRNAs is calculated as:

(total number of nucleotides assigned into different regions / total number of nucleotides in all mRNAs without gain/loss) * 100.

** The proportion of nucleotides lost in different isoforms is calculated as:

(total number of nucleotides assigned in introns in the collection of shorter isoforms [or the collection of long isoforms] / total number of nucleotides in the collection of longer isoforms [or the collection of short isoforms]) * 100.

About 0.07% of predicted target sites overlap, by at least 1 nt, a region flanking the CDS start or end position when window size = 5 nt, and about 1.0% when window size = 10 nt.
