# Supplementary material for: Transcriptome-Wide Prediction of miRNA Targets in Human and Mouse Using FASTH
Source: PLoS One. 2009 May 29;4(5):e5745. doi: 10.1371/journal.pone.0005745 (PMC2684643; doi:10.1371/journal.pone.0005745)
Supplement: Table S4 — Degree of overlap between FASTH prediction sets and those of other methods (0.05 MB DOC) [file pone.0005745.s007.doc]

**Supplementary Table S4**: Degree of overlap between FASTH prediction sets and those of other methods. Number of miRNA targets in human 3′ UTRs predicted by FASTH (this work), PicTar [1], TargetScan [2] and MiRanda [3]. In the *Shared targets %* column, the left-hand side gives the proportion of shared targets based on FASTH (*i.e*. number of shared targets / number of targets in FASTH), and the right-hand side gives the proportion of shared targets based on the other method (*i.e*. number of shared targets / number of targets in *e.g.* PicTar). TargetScan reports target sites for miRNA family members, each consisting of 1-8 miRNAs; here, if both the second method (for a single miRNA) and TargetScan (for a family) predict the same miRNA target, that target is counted as shared. Comparison sets: **A**, PicTar *versus* FASTH (168 miRNAs); **B**, TargetScan (205 families) *versus* FASTH (278 miRNAs); **C**, MiRanda versus FASTH (157 miRNAs).

| **Set** |  | **Number of targets from FASTH** | **Number of targets from other method** | **Number shared targets** | **Shared targets %** |
| --- | --- | --- | --- | --- | --- |
| A | WC bp at nt 2-7 | 77682 | 61820 | 12161 | 16 / 20 |
|  | WC bp at nt 2-8 | 50595 | 61820 | 9557 | 19 / 15 |
|  | WC bp at nt 2-7, <6 mismatches-and-GU-pairs at nt ≥15 | 38482 | 61820 | 5677 | 15 / 9 |
|  | WC bp at nt 2-8, <6 mismatches-and-GU-pairs at nt ≥15 | 24043 | 61820 | 4304 | 18 / 7 |
|  | | | | | |
| B | WC bp at nt 2-7 | 115456 | 46475 (44657*) | 14856 | 13 / - |
|  | WC bp at nt 2-8 | 73925 | 46475 (44657*) | 11917 | 16 / - |
|  | WC bp at nt 2-7, <6 mismatches-and-GU-pairs at nt ≥15 | 57597 | 46475 (44657*) | 7280 | 13 / - |
|  | WC bp at nt 2-8, <6 mismatches-and-GU-pairs at nt ≥15 | 35213 | 46475 (44657*) | 5668 | 16 / - |
|  | | | | | |
| C | WC bp at nt 2-7 | 68162 | 55887 (22896*) | 2543 | 4 / 11 |
|  | WC bp at nt 2-8 | 43601 | 55887 (22896*) | 1978 | 5 / 9 |
|  | WC bp at nt 2-7, <6 mismatches-and-GU-pairs at nt ≥15 | 33675 | 55887 (22896*) | 1340 | 4 / 6 |
|  | WC bp at nt 2-8, <6 mismatches-and-GU-pairs at nt ≥15 | 20878 | 55887 (22896*) | 979 | 5 / 4 |

***Note***

* Number of targets genes that could mapped to refSeq mRNAs.

***References***

1. Krek A, Grun D, Poy MN, Wolf R, Rosenberg L, *et al.* (2005) Combinatorial microRNA target predictions. Nat Genet 37: 495-500.

2. Lewis BP, Shih I, Jones-Rhoades MW, Bartel DP, Burge CB (2003) Prediction of mammalian microRNA targets. Cell 115: 787-798.

3. John B, Enright, AJ, Avavin A, Tuschl T, Sander C, Marks D (2004) Human microRNAs targets. PloS Biol 2: e363.
