# Supplementary material for: Transcriptome-Wide Prediction of miRNA Targets in Human and Mouse Using FASTH
Source: PLoS One. 2009 May 29;4(5):e5745. doi: 10.1371/journal.pone.0005745 (PMC2684643; doi:10.1371/journal.pone.0005745)
Supplement: Table S5 — Degree of overlap among prediction sets of three methods (0.03 MB DOC) [file pone.0005745.s008.doc]

**Supplementary Table S5:** Degree of overlap among prediction sets of three methods. Number of miRNA targets in human 3′ UTRs predicted by PicTar [1], TargetScan [2] and MiRanda [3]. TargetScan reports target sites for miRNA family members, each consisting of 1-8 miRNAs; here, if both the second method (for a single miRNA) and TargetScan (for a family) predict the same miRNA target, that target is counted as shared. References as in Supplementary Table S4.

|  | **Number of targets in PicTar** | **Number of targets in TargetScan** | **Number of targets in MiRanda** | **Shared with PicTar** | **Shared with TargetScan** | **Shared with MiRanda** |
| --- | --- | --- | --- | --- | --- | --- |
| **PicTar** | 61820 | 44657 | 22896 | - | 55%  (33723 / 61820) | 8%  (5026 / 61820) |
| **MiRanda** | 61820 | 44657 | 22896 | 22%  (5026 / 22896) | 26%  (5870 / 22896) | - |
