# Supplementary material for: Transcriptome-Wide Prediction of miRNA Targets in Human and Mouse Using FASTH
Source: PLoS One. 2009 May 29;4(5):e5745. doi: 10.1371/journal.pone.0005745 (PMC2684643; doi:10.1371/journal.pone.0005745)
Supplement: Table S6 — Top ten over-represented Gene Ontology terms for Biological Process (BP), Cellular Component (CC) and Molecular Function (MF) among mRNAs predicted as miRNA targets (0.07 MB DOC) [file pone.0005745.s009.doc]

**Supplementary Table 6.** Top ten over-represented Gene Ontology terms for Biological Process (BP), Cellular Component (CC) and Molecular Function (MF) among mRNAs predicted as miRNA targets. The number and proportion of predicted target genes with annotations within each category are shown at top of each section. The columns are: GO term, number and percent of predicted target genes associated with that term, p-value as given by DAVID, fold enrichment (FXR), and false discovery rate (FDR). In all, 10994 (69%), 10553 (67%) and 12055 (76%) of DAVID-annotated genes were associated with the GO-BP, GO-CC and GO-MF domains respectively.

| **Domain** | **Gene Ontology term** | **#** | **%** | **P-value** | **FXR** | **FDR** |
| --- | --- | --- | --- | --- | --- | --- |
| BP | protein kinase cascade | 268 | 1.7 | 6.10E-19 | 1.3 | 0 |
|  | enzyme linked receptor protein signalling pathway | 176 | 1.1 | 4.60E-12 | 1.3 | 0 |
|  | second-messenger-mediated signalling | 181 | 1.2 | 1.80E-11 | 1.3 | 0 |
|  | negative regulation of transcription | 159 | 1.0 | 8.90E-10 | 1.3 | 0 |
|  | transmembrane receptor protein tyrosine kinase signalling p/wy | 126 | 0.8 | 1.40E-08 | 1.3 | 0 |
|  | anti-apoptosis | 117 | 0.7 | 3.40E-08 | 1.3 | 0 |
|  | nucleocytoplasmic transport | 109 | 0.7 | 2.20E-07 | 1.3 | 0 |
|  | I-kappaB kinase/NF-kappaB cascade | 109 | 0.7 | 2.20E-07 | 1.3 | 0 |
|  | negative regulation of transcription, DNA-dependent | 107 | 0.7 | 3.50E-07 | 1.3 | 0 |
|  | Wnt receptor signalling pathway | 96 | 0.6 | 3.80E-07 | 1.3 | 0 |
|  | | | | | | |
| CC | plasma membrane | 1590 | 10.1 | 3.00E-60 | 1.2 | 0 |
|  | intrinsic to plasma membrane | 1143 | 7.3 | 2.10E-53 | 1.2 | 0 |
|  | integral to plasma membrane | 1135 | 7.2 | 1.20E-52 | 1.2 | 0 |
|  | cell fraction | 709 | 4.5 | 2.30E-23 | 1.2 | 0 |
|  | Golgi apparatus | 435 | 2.8 | 1.50E-19 | 1.2 | 0 |
|  | Golgi stack | 343 | 2.2 | 1.20E-18 | 1.2 | 0 |
|  | membrane fraction | 538 | 3.4 | 3.10E-18 | 1.2 | 0 |
|  | endoplasmic reticulum | 485 | 3.1 | 3.40E-13 | 1.2 | 0 |
|  | nucleoplasm | 235 | 1.5 | 1.80E-07 | 1.2 | 0 |
|  | soluble fraction | 179 | 1.1 | 8.70E-06 | 1.2 | 0 |
|  | | | | | | |
| MF | transcription factor binding | 296 | 1.9 | 5.70E-27 | 1.4 | 0 |
|  | transcription cofactor activity | 248 | 1.6 | 2.30E-23 | 1.4 | 0 |
|  | enzyme binding | 179 | 1.1 | 1.60E-19 | 1.4 | 0 |
|  | RNA polymerase II transcription factor activity | 202 | 1.3 | 1.90E-19 | 1.4 | 0 |
|  | alkali metal ion binding | 173 | 1.1 | 1.20E-18 | 1.4 | 0 |
|  | transcription coactivator activity | 154 | 1.0 | 1.40E-16 | 1.4 | 0 |
|  | identical protein binding | 163 | 1.0 | 5.40E-14 | 1.4 | 0 |
|  | potassium ion binding | 105 | 0.7 | 8.30E-11 | 1.4 | 0 |
|  | sodium ion binding | 84 | 0.5 | 1.40E-10 | 1.4 | 0 |
|  | manganese ion binding | 101 | 0.6 | 3.10E-10 | 1.4 | 0 |
