# Supplementary material for: Transcriptome-Wide Prediction of miRNA Targets in Human and Mouse Using FASTH
Source: PLoS One. 2009 May 29;4(5):e5745. doi: 10.1371/journal.pone.0005745 (PMC2684643; doi:10.1371/journal.pone.0005745)
Supplement: Table S7 — miRNAs and their predicted target sites selected for experimental validation (0.03 MB DOC) [file pone.0005745.s010.doc]

**Supplementary Table S7**. miRNAs and their predicted target sites selected for experimental validation.

***hsa-miR-15a:*** miRNAs such as hsa-miR-15a and hsa-miR-16-1 are highly expressed in many tissues. Genes encoding hsa-miR-15a and hsa-miR-16-1 are located at chromosome position 13q14.3 within a 30-kb region of loss in chronic lymphocytic leukemia (CLL) cells, and both are deleted and/or down-regulated in most CLL cell samples that have been analysed [1]. A recent study shows that hsa-miR-15a and miR-16 induce apoptosis by targeting the BCL2 mRNA [2], and our results confirm this targeting for hsa-miR-15a. We validated the TSPYL2 mRNA as a target of hsa-miR-15a; the binding site is predicted to be in its 3′UTR. TSPYL2, also known as cell division autoantigen 1 (CDA1), is a negative regulator of cell growth, and its over-expression inhibits cell growth [3, 4].

***hsa-miR-17-5p:*** hsa-miR-17-5p is located at chromosome 13q32-33, a region that is sometimes amplified in B cell malignancy. B cell lymphoma is generated by activation of the Myc oncogene. c-Myc binds directly in this region, activating the expression of a cluster of six miRNAs including hsa-miR-17-5p which, together with another of these miRNAs (hsa-miR-20a), negatively regulates expression of another c-Myc target, transcription factor E2F1 [5]. We predicted and have validated the mRNA for TNFSF12, also known as tumor necrosis factor-like weak inducer of apoptosis (TWEAK), as another target of hsa-miR-17-5p. TNFSF12 can induce apoptosis *via* multiple pathways, and stimulates numerous cellular responses including cell proliferation, migration, and pro-inflammatory molecule production [6]. hsa-miR-17-5p targets both known transcript isoforms; in one isoform the target is located in the coding region, and the 3′UTR in the other isoform.

***hsa-miR-324-3p:*** Given the over-representation of cell signalling genes as miRNA targets, and the high ranking of the Wnt signalling pathway in particular, we sought to validate the accuracy of predictions for a candidate miRNA that targets multiple mRNAs encoding components of the Wnt pathway. Wnt signalling is one of the master regulating signalling cascades involved in embryonic development and also plays important roles in homeostasis and tissue repair [7]. We predicted that the targets of hsa-miR-324-3p include mRNAs encoding proteins along much or all of the Wnt signalling pathway, including WNT9B (a secreted ligand that initiates Wnt signalling), DVL2 (a multi-module protein interior to the pathway), and CREBBP (a nuclear co-factor molecule involved the regulation of effector gene expression for the pathway). The gene encoding hsa-miR-324-3p is located about 17 kbp upstream of the gene encoding DVL2 on human chromosome 17. Both genes are transcribed from the same (minus) strand, possibly in the same primary transcript. However, our validation shows that the DVL2 message is not a target of miR-324. Although the predicted free energy of hsa -miR-324-3p binding to the DVL2 mRNA is very low (-41.5 kcal/mol), the duplex is predicted to have a single GU pair in the seed region. For this as well as for the two validated targets, the predicted target site is in the coding region.

1. Calin GA, Dumitru CD, Shimizu,M. Bichi,R. Zupo,S, *et al.* (2002) Frequent deletions and down-regulation of micro-RNA genes miR15 and miR16 at 13q14 in chronic lymphocytic leukaemia. Proc Natl Acad Sci U S A 99: 15524–15529.

2. Cimmino A, Calin GA, Fabbri M, Iorio MV, Ferracin M, *et al.* (2005) miR-15 and miR-16 induce apoptosis by targeting BCL2. Proc Natl Acad Sci U S A 102: 13944-133949.

3. Chai Z, Sarcevic B, Mawson A, Toh BH, (2001) SET-related cell division autoantigen-1 (CDA1) arrests cell growth. J Biol Chem 276: 33665-33674.

4. Tu Y, Wu W, Wu T, Cao Z, Wilkins R, *et al.* (2007) Antiproliferative autoantigen CDA1 transcriptionally up-regulates p21(Waf1/Cip1) by activating p53 and MEK/ERK1/2 MAPK pathways. J Biol Chem 282: 11722-11731.

5. O’Donnell KA, Wentzel EA, Zeller KI, Dang CV, Mendell JT (2005) c-Myc-regulated microRNAs modulate E2F1 expression. Nature 435: 839-843.

6. Winkles JA, Tran NL, and Berens ME (2006) TWEAK and Fn14: new molecular targets for cancer therapy? Cancer Lett 235: 11-17.

7. Nusse R (2005) Wnt signaling in disease and in development. Cell Res 15: 28-32.
