# Supplementary material for: Transcriptome-Wide Prediction of miRNA Targets in Human and Mouse Using FASTH
Source: PLoS One. 2009 May 29;4(5):e5745. doi: 10.1371/journal.pone.0005745 (PMC2684643; doi:10.1371/journal.pone.0005745)
Supplement: Table S8 — The 313 human miRNAs and 233 mouse miRNAs used as queries in this work (from miRBase release 7.0) (0.07 MB DOC) [file pone.0005745.s011.doc]

Human miRNA sequences from miRBase release 7.0

hsa-let-7a UGAGGUAGUAGGUUGUAUAGUU

hsa-let-7b UGAGGUAGUAGGUUGUGUGGUU

hsa-let-7c UGAGGUAGUAGGUUGUAUGGUU

hsa-let-7d AGAGGUAGUAGGUUGCAUAGU

hsa-let-7e UGAGGUAGGAGGUUGUAUAGU

hsa-let-7f UGAGGUAGUAGAUUGUAUAGUU

hsa-let-7g UGAGGUAGUAGUUUGUACAGU

hsa-let-7i UGAGGUAGUAGUUUGUGCUGU

hsa-miR-1 UGGAAUGUAAAGAAGUAUGUA

hsa-miR-100 AACCCGUAGAUCCGAACUUGUG

hsa-miR-101 UACAGUACUGUGAUAACUGAAG

hsa-miR-103 AGCAGCAUUGUACAGGGCUAUGA

hsa-miR-105 UCAAAUGCUCAGACUCCUGU

hsa-miR-106a AAAAGUGCUUACAGUGCAGGUAGC

hsa-miR-106b UAAAGUGCUGACAGUGCAGAU

hsa-miR-107 AGCAGCAUUGUACAGGGCUAUCA

hsa-miR-10a UACCCUGUAGAUCCGAAUUUGUG

hsa-miR-10b UACCCUGUAGAACCGAAUUUGU

hsa-miR-122a UGGAGUGUGACAAUGGUGUUUGU

hsa-miR-124a UUAAGGCACGCGGUGAAUGCCA

hsa-miR-125a UCCCUGAGACCCUUUAACCUGUG

hsa-miR-125b UCCCUGAGACCCUAACUUGUGA

hsa-miR-126 UCGUACCGUGAGUAAUAAUGC

hsa-miR-126* CAUUAUUACUUUUGGUACGCG

hsa-miR-127 UCGGAUCCGUCUGAGCUUGGCU

hsa-miR-128a UCACAGUGAACCGGUCUCUUUU

hsa-miR-128b UCACAGUGAACCGGUCUCUUUC

hsa-miR-129 CUUUUUGCGGUCUGGGCUUGC

hsa-miR-130a CAGUGCAAUGUUAAAAGGGCAU

hsa-miR-130b CAGUGCAAUGAUGAAAGGGCAU

hsa-miR-132 UAACAGUCUACAGCCAUGGUCG

hsa-miR-133a UUGGUCCCCUUCAACCAGCUGU

hsa-miR-133b UUGGUCCCCUUCAACCAGCUA

hsa-miR-134 UGUGACUGGUUGACCAGAGGG

hsa-miR-135a UAUGGCUUUUUAUUCCUAUGUGA

hsa-miR-135b UAUGGCUUUUCAUUCCUAUGUG

hsa-miR-136 ACUCCAUUUGUUUUGAUGAUGGA

hsa-miR-137 UAUUGCUUAAGAAUACGCGUAG

hsa-miR-138 AGCUGGUGUUGUGAAUC

hsa-miR-139 UCUACAGUGCACGUGUCU

hsa-miR-140 AGUGGUUUUACCCUAUGGUAG

hsa-miR-141 UAACACUGUCUGGUAAAGAUGG

hsa-miR-142-3p UGUAGUGUUUCCUACUUUAUGGA

hsa-miR-142-5p CAUAAAGUAGAAAGCACUAC

hsa-miR-143 UGAGAUGAAGCACUGUAGCUCA

hsa-miR-144 UACAGUAUAGAUGAUGUACUAG

hsa-miR-145 GUCCAGUUUUCCCAGGAAUCCCUU

hsa-miR-146a UGAGAACUGAAUUCCAUGGGUU

hsa-miR-146b UGAGAACUGAAUUCCAUAGGCU

hsa-miR-147 GUGUGUGGAAAUGCUUCUGC

hsa-miR-148a UCAGUGCACUACAGAACUUUGU

hsa-miR-148b UCAGUGCAUCACAGAACUUUGU

hsa-miR-149 UCUGGCUCCGUGUCUUCACUCC

hsa-miR-150 UCUCCCAACCCUUGUACCAGUG

hsa-miR-151 ACUAGACUGAAGCUCCUUGAGG

hsa-miR-152 UCAGUGCAUGACAGAACUUGGG

hsa-miR-153 UUGCAUAGUCACAAAAGUGA

hsa-miR-154 UAGGUUAUCCGUGUUGCCUUCG

hsa-miR-154* AAUCAUACACGGUUGACCUAUU

hsa-miR-155 UUAAUGCUAAUCGUGAUAGGGG

hsa-miR-15a UAGCAGCACAUAAUGGUUUGUG

hsa-miR-15b UAGCAGCACAUCAUGGUUUACA

hsa-miR-16 UAGCAGCACGUAAAUAUUGGCG

hsa-miR-17-3p ACUGCAGUGAAGGCACUUGU

hsa-miR-17-5p CAAAGUGCUUACAGUGCAGGUAGU

hsa-miR-181a AACAUUCAACGCUGUCGGUGAGU

hsa-miR-181b AACAUUCAUUGCUGUCGGUGGG

hsa-miR-181c AACAUUCAACCUGUCGGUGAGU

hsa-miR-181d AACAUUCAUUGUUGUCGGUGGGUU

hsa-miR-182 UUUGGCAAUGGUAGAACUCACA

hsa-miR-182* UGGUUCUAGACUUGCCAACUA

hsa-miR-183 UAUGGCACUGGUAGAAUUCACUG

hsa-miR-184 UGGACGGAGAACUGAUAAGGGU

hsa-miR-185 UGGAGAGAAAGGCAGUUC

hsa-miR-186 CAAAGAAUUCUCCUUUUGGGCUU

hsa-miR-187 UCGUGUCUUGUGUUGCAGCCG

hsa-miR-188 CAUCCCUUGCAUGGUGGAGGGU

hsa-miR-189 GUGCCUACUGAGCUGAUAUCAGU

hsa-miR-18a UAAGGUGCAUCUAGUGCAGAUA

hsa-miR-18b UAAGGUGCAUCUAGUGCAGUUA

hsa-miR-190 UGAUAUGUUUGAUAUAUUAGGU

hsa-miR-191 CAACGGAAUCCCAAAAGCAGCU

hsa-miR-191* GCUGCGCUUGGAUUUCGUCCCC

hsa-miR-192 CUGACCUAUGAAUUGACAGCC

hsa-miR-193a AACUGGCCUACAAAGUCCCAG

hsa-miR-193b AACUGGCCCUCAAAGUCCCGCUUU

hsa-miR-194 UGUAACAGCAACUCCAUGUGGA

hsa-miR-195 UAGCAGCACAGAAAUAUUGGC

hsa-miR-196a UAGGUAGUUUCAUGUUGUUGG

hsa-miR-196b UAGGUAGUUUCCUGUUGUUGG

hsa-miR-197 UUCACCACCUUCUCCACCCAGC

hsa-miR-198 GGUCCAGAGGGGAGAUAGG

hsa-miR-199a CCCAGUGUUCAGACUACCUGUUC

hsa-miR-199a* UACAGUAGUCUGCACAUUGGUU

hsa-miR-199b CCCAGUGUUUAGACUAUCUGUUC

hsa-miR-19a UGUGCAAAUCUAUGCAAAACUGA

hsa-miR-19b UGUGCAAAUCCAUGCAAAACUGA

hsa-miR-200a UAACACUGUCUGGUAACGAUGU

hsa-miR-200a* CAUCUUACCGGACAGUGCUGGA

hsa-miR-200b UAAUACUGCCUGGUAAUGAUGAC

hsa-miR-200c UAAUACUGCCGGGUAAUGAUGG

hsa-miR-202 AGAGGUAUAGGGCAUGGGAAAA

hsa-miR-202* UUUCCUAUGCAUAUACUUCUUU

hsa-miR-203 GUGAAAUGUUUAGGACCACUAG

hsa-miR-204 UUCCCUUUGUCAUCCUAUGCCU

hsa-miR-205 UCCUUCAUUCCACCGGAGUCUG

hsa-miR-206 UGGAAUGUAAGGAAGUGUGUGG

hsa-miR-208 AUAAGACGAGCAAAAAGCUUGU

hsa-miR-20a UAAAGUGCUUAUAGUGCAGGUAG

hsa-miR-20b CAAAGUGCUCAUAGUGCAGGUAG

hsa-miR-21 UAGCUUAUCAGACUGAUGUUGA

hsa-miR-210 CUGUGCGUGUGACAGCGGCUGA

hsa-miR-211 UUCCCUUUGUCAUCCUUCGCCU

hsa-miR-212 UAACAGUCUCCAGUCACGGCC

hsa-miR-213 ACCAUCGACCGUUGAUUGUACC

hsa-miR-214 ACAGCAGGCACAGACAGGCAG

hsa-miR-215 AUGACCUAUGAAUUGACAGAC

hsa-miR-216 UAAUCUCAGCUGGCAACUGUG

hsa-miR-217 UACUGCAUCAGGAACUGAUUGGAU

hsa-miR-218 UUGUGCUUGAUCUAACCAUGU

hsa-miR-219 UGAUUGUCCAAACGCAAUUCU

hsa-miR-22 AAGCUGCCAGUUGAAGAACUGU

hsa-miR-220 CCACACCGUAUCUGACACUUU

hsa-miR-221 AGCUACAUUGUCUGCUGGGUUUC

hsa-miR-222 AGCUACAUCUGGCUACUGGGUCUC

hsa-miR-223 UGUCAGUUUGUCAAAUACCCC

hsa-miR-224 CAAGUCACUAGUGGUUCCGUUUA

hsa-miR-23a AUCACAUUGCCAGGGAUUUCC

hsa-miR-23b AUCACAUUGCCAGGGAUUACC

hsa-miR-24 UGGCUCAGUUCAGCAGGAACAG

hsa-miR-25 CAUUGCACUUGUCUCGGUCUGA

hsa-miR-26a UUCAAGUAAUCCAGGAUAGGC

hsa-miR-26b UUCAAGUAAUUCAGGAUAGGUU

hsa-miR-27a UUCACAGUGGCUAAGUUCCGC

hsa-miR-27b UUCACAGUGGCUAAGUUCUGC

hsa-miR-28 AAGGAGCUCACAGUCUAUUGAG

hsa-miR-296 AGGGCCCCCCCUCAAUCCUGU

hsa-miR-299-3p UAUGUGGGAUGGUAAACCGCUU

hsa-miR-299-5p UGGUUUACCGUCCCACAUACAU

hsa-miR-29a UAGCACCAUCUGAAAUCGGUU

hsa-miR-29b UAGCACCAUUUGAAAUCAGUGUU

hsa-miR-29c UAGCACCAUUUGAAAUCGGU

hsa-miR-301 CAGUGCAAUAGUAUUGUCAAAGC

hsa-miR-302a UAAGUGCUUCCAUGUUUUGGUGA

hsa-miR-302a* UAAACGUGGAUGUACUUGCUUU

hsa-miR-302b UAAGUGCUUCCAUGUUUUAGUAG

hsa-miR-302b* ACUUUAACAUGGAAGUGCUUUCU

hsa-miR-302c UAAGUGCUUCCAUGUUUCAGUGG

hsa-miR-302c* UUUAACAUGGGGGUACCUGCUG

hsa-miR-302d UAAGUGCUUCCAUGUUUGAGUGU

hsa-miR-30a-3p CUUUCAGUCGGAUGUUUGCAGC

hsa-miR-30a-5p UGUAAACAUCCUCGACUGGAAG

hsa-miR-30b UGUAAACAUCCUACACUCAGCU

hsa-miR-30c UGUAAACAUCCUACACUCUCAGC

hsa-miR-30d UGUAAACAUCCCCGACUGGAAG

hsa-miR-30e-3p CUUUCAGUCGGAUGUUUACAGC

hsa-miR-30e-5p UGUAAACAUCCUUGACUGGA

hsa-miR-31 GGCAAGAUGCUGGCAUAGCUG

hsa-miR-32 UAUUGCACAUUACUAAGUUGC

hsa-miR-320 AAAAGCUGGGUUGAGAGGGCGAA

hsa-miR-323 GCACAUUACACGGUCGACCUCU

hsa-miR-324-3p CCACUGCCCCAGGUGCUGCUGG

hsa-miR-324-5p CGCAUCCCCUAGGGCAUUGGUGU

hsa-miR-325 CCUAGUAGGUGUCCAGUAAGUGU

hsa-miR-326 CCUCUGGGCCCUUCCUCCAG

hsa-miR-328 CUGGCCCUCUCUGCCCUUCCGU

hsa-miR-329 AACACACCUGGUUAACCUCUUU

hsa-miR-33 GUGCAUUGUAGUUGCAUUG

hsa-miR-330 GCAAAGCACACGGCCUGCAGAGA

hsa-miR-331 GCCCCUGGGCCUAUCCUAGAA

hsa-miR-335 UCAAGAGCAAUAACGAAAAAUGU

hsa-miR-337 UCCAGCUCCUAUAUGAUGCCUUU

hsa-miR-338 UCCAGCAUCAGUGAUUUUGUUGA

hsa-miR-339 UCCCUGUCCUCCAGGAGCUCA

hsa-miR-340 UCCGUCUCAGUUACUUUAUAGCC

hsa-miR-342 UCUCACACAGAAAUCGCACCCGUC

hsa-miR-345 UGCUGACUCCUAGUCCAGGGC

hsa-miR-346 UGUCUGCCCGCAUGCCUGCCUCU

hsa-miR-34a UGGCAGUGUCUUAGCUGGUUGUU

hsa-miR-34b UAGGCAGUGUCAUUAGCUGAUUG

hsa-miR-34c AGGCAGUGUAGUUAGCUGAUUGC

hsa-miR-361 UUAUCAGAAUCUCCAGGGGUAC

hsa-miR-362 AAUCCUUGGAACCUAGGUGUGAG

hsa-miR-363 AUUGCACGGUAUCCAUCUGUAA

hsa-miR-365 UAAUGCCCCUAAAAAUCCUUAU

hsa-miR-367 AAUUGCACUUUAGCAAUGGUGA

hsa-miR-368 ACAUAGAGGAAAUUCCACGUUU

hsa-miR-369-3p AAUAAUACAUGGUUGAUCUUU

hsa-miR-369-5p AGAUCGACCGUGUUAUAUUCGC

hsa-miR-370 GCCUGCUGGGGUGGAACCUGG

hsa-miR-371 GUGCCGCCAUCUUUUGAGUGU

hsa-miR-372 AAAGUGCUGCGACAUUUGAGCGU

hsa-miR-373 GAAGUGCUUCGAUUUUGGGGUGU

hsa-miR-373* ACUCAAAAUGGGGGCGCUUUCC

hsa-miR-374 UUAUAAUACAACCUGAUAAGUG

hsa-miR-375 UUUGUUCGUUCGGCUCGCGUGA

hsa-miR-376a AUCAUAGAGGAAAAUCCACGU

hsa-miR-376b AUCAUAGAGGAAAAUCCAUGUU

hsa-miR-377 AUCACACAAAGGCAACUUUUGU

hsa-miR-378 CUCCUGACUCCAGGUCCUGUGU

hsa-miR-379 UGGUAGACUAUGGAACGUA

hsa-miR-380-3p UAUGUAAUAUGGUCCACAUCUU

hsa-miR-380-5p UGGUUGACCAUAGAACAUGCGC

hsa-miR-381 UAUACAAGGGCAAGCUCUCUGU

hsa-miR-382 GAAGUUGUUCGUGGUGGAUUCG

hsa-miR-383 AGAUCAGAAGGUGAUUGUGGCU

hsa-miR-384 AUUCCUAGAAAUUGUUCAUA

hsa-miR-409-3p CGAAUGUUGCUCGGUGAACCCCU

hsa-miR-409-5p AGGUUACCCGAGCAACUUUGCA

hsa-miR-410 AAUAUAACACAGAUGGCCUGUU

hsa-miR-412 ACUUCACCUGGUCCACUAGCCGU

hsa-miR-422a CUGGACUUAGGGUCAGAAGGCC

hsa-miR-422b CUGGACUUGGAGUCAGAAGGCC

hsa-miR-423 AGCUCGGUCUGAGGCCCCUCAG

hsa-miR-424 CAGCAGCAAUUCAUGUUUUGAA

hsa-miR-425 AUCGGGAAUGUCGUGUCCGCC

hsa-miR-429 UAAUACUGUCUGGUAAAACCGU

hsa-miR-431 UGUCUUGCAGGCCGUCAUGCA

hsa-miR-432 UCUUGGAGUAGGUCAUUGGGUGG

hsa-miR-432* CUGGAUGGCUCCUCCAUGUCU

hsa-miR-433 AUCAUGAUGGGCUCCUCGGUGU

hsa-miR-448 UUGCAUAUGUAGGAUGUCCCAU

hsa-miR-449 UGGCAGUGUAUUGUUAGCUGGU

hsa-miR-450 UUUUUGCGAUGUGUUCCUAAUA

hsa-miR-451 AAACCGUUACCAUUACUGAGUUU

hsa-miR-452 UGUUUGCAGAGGAAACUGAGAC

hsa-miR-452* UCAGUCUCAUCUGCAAAGAAG

hsa-miR-453 GAGGUUGUCCGUGGUGAGUUCG

hsa-miR-485-3p GUCAUACACGGCUCUCCUCU

hsa-miR-485-5p AGAGGCUGGCCGUGAUGAAUUC

hsa-miR-488 CCCAGAUAAUGGCACUCUCAA

hsa-miR-489 AGUGACAUCACAUAUACGGCAGC

hsa-miR-490 CAACCUGGAGGACUCCAUGCUG

hsa-miR-491 AGUGGGGAACCCUUCCAUGAGGA

hsa-miR-492 AGGACCUGCGGGACAAGAUUCUU

hsa-miR-493 UUGUACAUGGUAGGCUUUCAUU

hsa-miR-494 UGAAACAUACACGGGAAACCUCUU

hsa-miR-495 AAACAAACAUGGUGCACUUCUUU

hsa-miR-496 AUUACAUGGCCAAUCUC

hsa-miR-497 CAGCAGCACACUGUGGUUUGU

hsa-miR-498 UUUCAAGCCAGGGGGCGUUUUUC

hsa-miR-499 UUAAGACUUGCAGUGAUGUUUAA

hsa-miR-500 AUGCACCUGGGCAAGGAUUCUG

hsa-miR-501 AAUCCUUUGUCCCUGGGUGAGA

hsa-miR-502 AUCCUUGCUAUCUGGGUGCUA

hsa-miR-503 UAGCAGCGGGAACAGUUCUGCAG

hsa-miR-504 AGACCCUGGUCUGCACUCUAU

hsa-miR-505 GUCAACACUUGCUGGUUUCCUC

hsa-miR-506 UAAGGCACCCUUCUGAGUAGA

hsa-miR-507 UUUUGCACCUUUUGGAGUGAA

hsa-miR-508 UGAUUGUAGCCUUUUGGAGUAGA

hsa-miR-509 UGAUUGGUACGUCUGUGGGUAGA

hsa-miR-510 UACUCAGGAGAGUGGCAAUCACA

hsa-miR-511 GUGUCUUUUGCUCUGCAGUCA

hsa-miR-512-3p AAGUGCUGUCAUAGCUGAGGUC

hsa-miR-512-5p CACUCAGCCUUGAGGGCACUUUC

hsa-miR-513 UUCACAGGGAGGUGUCAUUUAU

hsa-miR-514 AUUGACACUUCUGUGAGUAG

hsa-miR-515-3p GAGUGCCUUCUUUUGGAGCGU

hsa-miR-515-5p UUCUCCAAAAGAAAGCACUUUCUG

hsa-miR-516-3p UGCUUCCUUUCAGAGGGU

hsa-miR-516-5p AUCUGGAGGUAAGAAGCACUUU

hsa-miR-517* CCUCUAGAUGGAAGCACUGUCU

hsa-miR-517a AUCGUGCAUCCCUUUAGAGUGUU

hsa-miR-517b UCGUGCAUCCCUUUAGAGUGUU

hsa-miR-517c AUCGUGCAUCCUUUUAGAGUGU

hsa-miR-518a AAAGCGCUUCCCUUUGCUGGA

hsa-miR-518a-2* UCUGCAAAGGGAAGCCCUUU

hsa-miR-518b CAAAGCGCUCCCCUUUAGAGGU

hsa-miR-518c CAAAGCGCUUCUCUUUAGAGUG

hsa-miR-518c* UCUCUGGAGGGAAGCACUUUCUG

hsa-miR-518d CAAAGCGCUUCCCUUUGGAGC

hsa-miR-518e AAAGCGCUUCCCUUCAGAGUGU

hsa-miR-518f AAAGCGCUUCUCUUUAGAGGA

hsa-miR-518f* CUCUAGAGGGAAGCACUUUCUCU

hsa-miR-519a AAAGUGCAUCCUUUUAGAGUGUUAC

hsa-miR-519b AAAGUGCAUCCUUUUAGAGGUUU

hsa-miR-519c AAAGUGCAUCUUUUUAGAGGAU

hsa-miR-519d CAAAGUGCCUCCCUUUAGAGUGU

hsa-miR-519e AAAGUGCCUCCUUUUAGAGUGU

hsa-miR-519e* UUCUCCAAAAGGGAGCACUUUC

hsa-miR-520a AAAGUGCUUCCCUUUGGACUGU

hsa-miR-520a* CUCCAGAGGGAAGUACUUUCU

hsa-miR-520b AAAGUGCUUCCUUUUAGAGGG

hsa-miR-520c AAAGUGCUUCCUUUUAGAGGGUU

hsa-miR-520d AAAGUGCUUCUCUUUGGUGGGUU

hsa-miR-520d* UCUACAAAGGGAAGCCCUUUCUG

hsa-miR-520e AAAGUGCUUCCUUUUUGAGGG

hsa-miR-520f AAGUGCUUCCUUUUAGAGGGUU

hsa-miR-520g ACAAAGUGCUUCCCUUUAGAGUGU

hsa-miR-520h ACAAAGUGCUUCCCUUUAGAGU

hsa-miR-521 AACGCACUUCCCUUUAGAGUGU

hsa-miR-522 AAAAUGGUUCCCUUUAGAGUGUU

hsa-miR-523 AACGCGCUUCCCUAUAGAGGG

hsa-miR-524 GAAGGCGCUUCCCUUUGGAGU

hsa-miR-524* CUACAAAGGGAAGCACUUUCUC

hsa-miR-525 CUCCAGAGGGAUGCACUUUCU

hsa-miR-525* GAAGGCGCUUCCCUUUAGAGC

hsa-miR-526a CUCUAGAGGGAAGCACUUUCU

hsa-miR-526b CUCUUGAGGGAAGCACUUUCUGUU

hsa-miR-526b* AAAGUGCUUCCUUUUAGAGGC

hsa-miR-526c CUCUAGAGGGAAGCGCUUUCUGUU

hsa-miR-527 CUGCAAAGGGAAGCCCUUUCU

hsa-miR-7 UGGAAGACUAGUGAUUUUGUUG

hsa-miR-9 UCUUUGGUUAUCUAGCUGUAUGA

hsa-miR-9* UAAAGCUAGAUAACCGAAAGU

hsa-miR-92 UAUUGCACUUGUCCCGGCCUG

hsa-miR-93 AAAGUGCUGUUCGUGCAGGUAG

hsa-miR-95 UUCAACGGGUAUUUAUUGAGCA

hsa-miR-96 UUUGGCACUAGCACAUUUUUGC

hsa-miR-98 UGAGGUAGUAAGUUGUAUUGUU

hsa-miR-99a AACCCGUAGAUCCGAUCUUGUG

hsa-miR-99b CACCCGUAGAACCGACCUUGCG

Mouse miRNA sequences from miRBase release 7.0

mmu-let-7a UGAGGUAGUAGGUUGUAUAGU

mmu-let-7b UGAGGUAGUAGGUUGUGUGGUU

mmu-let-7c UGAGGUAGUAGGUUGUAUGGUU

mmu-let-7d AGAGGUAGUAGGUUGCAUAGU

mmu-let-7d* CUAUACGACCUGCUGCCUUUCU

mmu-let-7e UGAGGUAGGAGGUUGUAUAGU

mmu-let-7f UGAGGUAGUAGAUUGUAUAGU

mmu-let-7g UGAGGUAGUAGUUUGUACAGU

mmu-let-7i UGAGGUAGUAGUUUGUGCUGU

mmu-miR-1 UGGAAUGUAAAGAAGUAUGUA

mmu-miR-100 AACCCGUAGAUCCGAACUUGUG

mmu-miR-101a UACAGUACUGUGAUAACUGAAG

mmu-miR-101b UACAGUACUGUGAUAGCUGAAG

mmu-miR-103 AGCAGCAUUGUACAGGGCUAUGA

mmu-miR-106a CAAAGUGCUAACAGUGCAGGUA

mmu-miR-106b UAAAGUGCUGACAGUGCAGAU

mmu-miR-107 AGCAGCAUUGUACAGGGCUAUCA

mmu-miR-10a UACCCUGUAGAUCCGAAUUUGUG

mmu-miR-10b CCCUGUAGAACCGAAUUUGUGU

mmu-miR-122a UGGAGUGUGACAAUGGUGUUUGU

mmu-miR-124a UAAGGCACGCGGUGAAUGCC

mmu-miR-125a UCCCUGAGACCCUUUAACCUGUG

mmu-miR-125b UCCCUGAGACCCUAACUUGUGA

mmu-miR-126-3p UCGUACCGUGAGUAAUAAUGC

mmu-miR-126-5p CAUUAUUACUUUUGGUACGCG

mmu-miR-127 UCGGAUCCGUCUGAGCUUGGC

mmu-miR-128a UCACAGUGAACCGGUCUCUUUU

mmu-miR-128b UCACAGUGAACCGGUCUCUUUC

mmu-miR-129-3p AAGCCCUUACCCCAAAAAGCAU

mmu-miR-129-5p CUUUUUGCGGUCUGGGCUUGCU

mmu-miR-130a CAGUGCAAUGUUAAAAGGGCAU

mmu-miR-130b CAGUGCAAUGAUGAAAGGGCAU

mmu-miR-132 UAACAGUCUACAGCCAUGGUCG

mmu-miR-133a UUGGUCCCCUUCAACCAGCUGU

mmu-miR-133b UUGGUCCCCUUCAACCAGCUA

mmu-miR-134 UGUGACUGGUUGACCAGAGGGG

mmu-miR-135a UAUGGCUUUUUAUUCCUAUGUGA

mmu-miR-135b UAUGGCUUUUCAUUCCUAUGUG

mmu-miR-136 ACUCCAUUUGUUUUGAUGAUGGA

mmu-miR-137 UUAUUGCUUAAGAAUACGCGUAG

mmu-miR-138 AGCUGGUGUUGUGAAUC

mmu-miR-139 UCUACAGUGCACGUGUCU

mmu-miR-140 CAGUGGUUUUACCCUAUGGUAG

mmu-miR-140* UACCACAGGGUAGAACCACGGA

mmu-miR-141 UAACACUGUCUGGUAAAGAUGG

mmu-miR-142-3p UGUAGUGUUUCCUACUUUAUGG

mmu-miR-142-5p CAUAAAGUAGAAAGCACUAC

mmu-miR-143 UGAGAUGAAGCACUGUAGCUCA

mmu-miR-144 UACAGUAUAGAUGAUGUACUAG

mmu-miR-145 GUCCAGUUUUCCCAGGAAUCCCUU

mmu-miR-146 UGAGAACUGAAUUCCAUGGGUU

mmu-miR-148a UCAGUGCACUACAGAACUUUGU

mmu-miR-148b UCAGUGCAUCACAGAACUUUGU

mmu-miR-149 UCUGGCUCCGUGUCUUCACUCC

mmu-miR-150 UCUCCCAACCCUUGUACCAGUG

mmu-miR-151 CUAGACUGAGGCUCCUUGAGG

mmu-miR-152 UCAGUGCAUGACAGAACUUGGG

mmu-miR-153 UUGCAUAGUCACAAAAGUGAUC

mmu-miR-154 UAGGUUAUCCGUGUUGCCUUCG

mmu-miR-155 UUAAUGCUAAUUGUGAUAGGGG

mmu-miR-15a UAGCAGCACAUAAUGGUUUGUG

mmu-miR-15b UAGCAGCACAUCAUGGUUUACA

mmu-miR-16 UAGCAGCACGUAAAUAUUGGCG

mmu-miR-17-3p ACUGCAGUGAGGGCACUUGUA

mmu-miR-17-5p CAAAGUGCUUACAGUGCAGGUAGU

mmu-miR-18 UAAGGUGCAUCUAGUGCAGAUA

mmu-miR-181a AACAUUCAACGCUGUCGGUGAGU

mmu-miR-181b AACAUUCAUUGCUGUCGGUGGG

mmu-miR-181c AACAUUCAACCUGUCGGUGAGU

mmu-miR-182 UUUGGCAAUGGUAGAACUCACA

mmu-miR-183 UAUGGCACUGGUAGAAUUCACUG

mmu-miR-184 UGGACGGAGAACUGAUAAGGGU

mmu-miR-185 UGGAGAGAAAGGCAGUUC

mmu-miR-186 CAAAGAAUUCUCCUUUUGGGCUU

mmu-miR-187 UCGUGUCUUGUGUUGCAGCCGG

mmu-miR-188 CAUCCCUUGCAUGGUGGAGGGU

mmu-miR-189 GUGCCUACUGAGCUGAUAUCAGU

mmu-miR-190 UGAUAUGUUUGAUAUAUUAGGU

mmu-miR-191 CAACGGAAUCCCAAAAGCAGCU

mmu-miR-192 CUGACCUAUGAAUUGACA

mmu-miR-193 AACUGGCCUACAAAGUCCCAG

mmu-miR-194 UGUAACAGCAACUCCAUGUGGA

mmu-miR-195 UAGCAGCACAGAAAUAUUGGC

mmu-miR-196a UAGGUAGUUUCAUGUUGUUGG

mmu-miR-196b UAGGUAGUUUCCUGUUGUUGG

mmu-miR-199a CCCAGUGUUCAGACUACCUGUUC

mmu-miR-199a* UACAGUAGUCUGCACAUUGGUU

mmu-miR-199b CCCAGUGUUUAGACUACCUGUUC

mmu-miR-19a UGUGCAAAUCUAUGCAAAACUGA

mmu-miR-19b UGUGCAAAUCCAUGCAAAACUGA

mmu-miR-20 UAAAGUGCUUAUAGUGCAGGUAG

mmu-miR-200a UAACACUGUCUGGUAACGAUGU

mmu-miR-200b UAAUACUGCCUGGUAAUGAUGAC

mmu-miR-200c UAAUACUGCCGGGUAAUGAUGG

mmu-miR-201 UACUCAGUAAGGCAUUGUUCU

mmu-miR-202 AGAGGUAUAGCGCAUGGGAAGA

mmu-miR-203 UGAAAUGUUUAGGACCACUAG

mmu-miR-204 UUCCCUUUGUCAUCCUAUGCCUG

mmu-miR-205 UCCUUCAUUCCACCGGAGUCUG

mmu-miR-206 UGGAAUGUAAGGAAGUGUGUGG

mmu-miR-207 GCUUCUCCUGGCUCUCCUCCCUC

mmu-miR-208 AUAAGACGAGCAAAAAGCUUGU

mmu-miR-21 UAGCUUAUCAGACUGAUGUUGA

mmu-miR-210 CUGUGCGUGUGACAGCGGCUGA

mmu-miR-211 UUCCCUUUGUCAUCCUUUGCCU

mmu-miR-212 UAACAGUCUCCAGUCACGGCC

mmu-miR-213 ACCAUCGACCGUUGAUUGUACC

mmu-miR-214 ACAGCAGGCACAGACAGGCAG

mmu-miR-215 AUGACCUAUGAUUUGACAGAC

mmu-miR-216 UAAUCUCAGCUGGCAACUGUG

mmu-miR-217 UACUGCAUCAGGAACUGACUGGAU

mmu-miR-218 UUGUGCUUGAUCUAACCAUGU

mmu-miR-219 UGAUUGUCCAAACGCAAUUCU

mmu-miR-22 AAGCUGCCAGUUGAAGAACUGU

mmu-miR-221 AGCUACAUUGUCUGCUGGGUUU

mmu-miR-222 AGCUACAUCUGGCUACUGGGUCUC

mmu-miR-223 UGUCAGUUUGUCAAAUACCCC

mmu-miR-224 UAAGUCACUAGUGGUUCCGUUUA

mmu-miR-23a AUCACAUUGCCAGGGAUUUCC

mmu-miR-23b AUCACAUUGCCAGGGAUUACC

mmu-miR-24 UGGCUCAGUUCAGCAGGAACAG

mmu-miR-25 CAUUGCACUUGUCUCGGUCUGA

mmu-miR-26a UUCAAGUAAUCCAGGAUAGGC

mmu-miR-26b UUCAAGUAAUUCAGGAUAGGUU

mmu-miR-27a UUCACAGUGGCUAAGUUCCGC

mmu-miR-27b UUCACAGUGGCUAAGUUCUGC

mmu-miR-28 AAGGAGCUCACAGUCUAUUGAG

mmu-miR-290 CUCAAACUAUGGGGGCACUUUUU

mmu-miR-291-3p AAAGUGCUUCCACUUUGUGUGCC

mmu-miR-291-5p CAUCAAAGUGGAGGCCCUCUCU

mmu-miR-292-3p AAGUGCCGCCAGGUUUUGAGUGU

mmu-miR-292-5p ACUCAAACUGGGGGCUCUUUUG

mmu-miR-293 AGUGCCGCAGAGUUUGUAGUGU

mmu-miR-294 AAAGUGCUUCCCUUUUGUGUGU

mmu-miR-295 AAAGUGCUACUACUUUUGAGUCU

mmu-miR-296 AGGGCCCCCCCUCAAUCCUGU

mmu-miR-297 AUGUAUGUGUGCAUGUGCAUG

mmu-miR-298 GGCAGAGGAGGGCUGUUCUUCC

mmu-miR-299 UGGUUUACCGUCCCACAUACAU

mmu-miR-29a UAGCACCAUCUGAAAUCGGUU

mmu-miR-29b UAGCACCAUUUGAAAUCAGUGUU

mmu-miR-29c UAGCACCAUUUGAAAUCGGU

mmu-miR-300 UAUGCAAGGGCAAGCUCUCUUC

mmu-miR-301 CAGUGCAAUAGUAUUGUCAAAGC

mmu-miR-302 UAAGUGCUUCCAUGUUUUGGUGA

mmu-miR-30a-3p CUUUCAGUCGGAUGUUUGCAGC

mmu-miR-30a-5p UGUAAACAUCCUCGACUGGAAG

mmu-miR-30b UGUAAACAUCCUACACUCAGCU

mmu-miR-30c UGUAAACAUCCUACACUCUCAGC

mmu-miR-30d UGUAAACAUCCCCGACUGGAAG

mmu-miR-30e UGUAAACAUCCUUGACUGGA

mmu-miR-30e* CUUUCAGUCGGAUGUUUACAG

mmu-miR-31 AGGCAAGAUGCUGGCAUAGCUG

mmu-miR-32 UAUUGCACAUUACUAAGUUGC

mmu-miR-320 AAAAGCUGGGUUGAGAGGGCGAA

mmu-miR-322 AAACAUGAAGCGCUGCAACA

mmu-miR-323 GCACAUUACACGGUCGACCUCU

mmu-miR-324-3p CCACUGCCCCAGGUGCUGCUGG

mmu-miR-324-5p CGCAUCCCCUAGGGCAUUGGUG

mmu-miR-325 CCUAGUAGGUGCUCAGUAAGUGU

mmu-miR-326 CCUCUGGGCCCUUCCUCCAGU

mmu-miR-328 CUGGCCCUCUCUGCCCUUCCGU

mmu-miR-329 AACACACCCAGCUAACCUUUUU

mmu-miR-33 GUGCAUUGUAGUUGCAUUG

mmu-miR-330 GCAAAGCACAGGGCCUGCAGAGA

mmu-miR-331 GCCCCUGGGCCUAUCCUAGAA

mmu-miR-335 UCAAGAGCAAUAACGAAAAAUGU

mmu-miR-337 UUCAGCUCCUAUAUGAUGCCUUU

mmu-miR-338 UCCAGCAUCAGUGAUUUUGUUGA

mmu-miR-339 UCCCUGUCCUCCAGGAGCUCA

mmu-miR-340 UCCGUCUCAGUUACUUUAUAGCC

mmu-miR-341 UCGAUCGGUCGGUCGGUCAGU

mmu-miR-342 UCUCACACAGAAAUCGCACCCGUC

mmu-miR-344 UGAUCUAGCCAAAGCCUGACUGU

mmu-miR-345 UGCUGACCCCUAGUCCAGUGC

mmu-miR-346 UGUCUGCCCGAGUGCCUGCCUCU

mmu-miR-34a UGGCAGUGUCUUAGCUGGUUGUU

mmu-miR-34b UAGGCAGUGUAAUUAGCUGAUUG

mmu-miR-34c AGGCAGUGUAGUUAGCUGAUUGC

mmu-miR-350 UUCACAAAGCCCAUACACUUUCA

mmu-miR-351 UCCCUGAGGAGCCCUUUGAGCCUG

mmu-miR-361 UUAUCAGAAUCUCCAGGGGUAC

mmu-miR-363 AUUGCACGGUAUCCAUCUGUAA

mmu-miR-365 UAAUGCCCCUAAAAAUCCUUAU

mmu-miR-370 GCCUGCUGGGGUGGAACCUGGUU

mmu-miR-375 UUUGUUCGUUCGGCUCGCGUGA

mmu-miR-376a AUCGUAGAGGAAAAUCCACGU

mmu-miR-376b AUCAUAGAGGAACAUCCACUUU

mmu-miR-377 AUCACACAAAGGCAACUUUUGU

mmu-miR-378 CUCCUGACUCCAGGUCCUGUGU

mmu-miR-379 UGGUAGACUAUGGAACGUAGG

mmu-miR-380-3p UAUGUAGUAUGGUCCACAUCUU

mmu-miR-380-5p UGGUUGACCAUAGAACAUGCGC

mmu-miR-381 UAUACAAGGGCAAGCUCUCUGU

mmu-miR-382 GAAGUUGUUCGUGGUGGAUUCG

mmu-miR-383 AGAUCAGAAGGUGACUGUGGCU

mmu-miR-384 AUUCCUAGAAAUUGUUCACA

mmu-miR-409 GAAUGUUGCUCGGUGAACCCCUU

mmu-miR-410 AAUAUAACACAGAUGGCCUGUU

mmu-miR-411 AACACGGUCCACUAACCCUCAGU

mmu-miR-412 ACUUCACCUGGUCCACUAGCCGU

mmu-miR-424 CAGCAGCAAUUCAUGUUUUGGA

mmu-miR-425 AUCGGGAAUGUCGUGUCCGCC

mmu-miR-429 UAAUACUGUCUGGUAAUGCCGU

mmu-miR-431 UGUCUUGCAGGCCGUCAUGCAGG

mmu-miR-433-3p AUCAUGAUGGGCUCCUCGGUGU

mmu-miR-433-5p UACGGUGAGCCUGUCAUUAUUC

mmu-miR-434-3p UUUGAACCAUCACUCGACUCC

mmu-miR-434-5p AGCUCGACUCAUGGUUUGAACC

mmu-miR-448 UUGCAUAUGUAGGAUGUCCCAU

mmu-miR-449 UGGCAGUGUAUUGUUAGCUGGU

mmu-miR-450 UUUUUGCGAUGUGUUCCUAAUA

mmu-miR-451 AAACCGUUACCAUUACUGAGUU

mmu-miR-452 UGUUUGCAGAGGAAACUGAGAC

mmu-miR-463 UACCUAAUUUGUUGUCCAUCA

mmu-miR-464 UACCAAGUUUAUUCUGUGAGAUA

mmu-miR-465 UAUUUAGAAUGGCACUGAUGUGA

mmu-miR-466 AUACAUACACGCACACAUAAGAC

mmu-miR-467 AUAUACAUACACACACCUACAC

mmu-miR-468 UAUGACUGAUGUGCGUGUGUCUG

mmu-miR-469 UGCCUCUUUCAUUGAUCUUGGUGUCC

mmu-miR-470 UUCUUGGACUGGCACUGGUGA

mmu-miR-471 UACGUAGUAUAGUGCUUUUCACA

mmu-miR-7 UGGAAGACUAGUGAUUUUGUUG

mmu-miR-7b UGGAAGACUUGUGAUUUUGUU

mmu-miR-9 UCUUUGGUUAUCUAGCUGUAUG

mmu-miR-9* UAAAGCUAGAUAACCGAAAGU

mmu-miR-92 UAUUGCACUUGUCCCGGCCUG

mmu-miR-93 CAAAGUGCUGUUCGUGCAGGUAG

mmu-miR-96 UUUGGCACUAGCACAUUUUUGCU

mmu-miR-98 UGAGGUAGUAAGUUGUAUUGUU

mmu-miR-99a ACCCGUAGAUCCGAUCUUGU

mmu-miR-99b CACCCGUAGAACCGACCUUGCG
