# Supplementary material for: Transcriptome-Wide Prediction of miRNA Targets in Human and Mouse Using FASTH
Source: PLoS One. 2009 May 29;4(5):e5745. doi: 10.1371/journal.pone.0005745 (PMC2684643; doi:10.1371/journal.pone.0005745)
Supplement: Table S13 — The 181 orthologous human and mouse miRNAs that are identical in sequences at nucleotide positions 1–8 (0.04 MB DOC) [file pone.0005745.s016.doc]

Orthologous human and mouse miRNA sequences

hsa-let-7a UGAGGUAGUAGGUUGUAUAGUU mmu-let-7a UGAGGUAGUAGGUUGUAUAGUU

hsa-let-7b UGAGGUAGUAGGUUGUGUGGUU mmu-let-7b UGAGGUAGUAGGUUGUGUGGUU

hsa-let-7c UGAGGUAGUAGGUUGUAUGGUU mmu-let-7c UGAGGUAGUAGGUUGUAUGGUU

hsa-let-7d AGAGGUAGUAGGUUGCAUAGU mmu-let-7d AGAGGUAGUAGGUUGCAUAGU

hsa-let-7e UGAGGUAGGAGGUUGUAUAGU mmu-let-7e UGAGGUAGGAGGUUGUAUAGUU

hsa-let-7f UGAGGUAGUAGAUUGUAUAGUU mmu-let-7f UGAGGUAGUAGAUUGUAUAGU

hsa-let-7g UGAGGUAGUAGUUUGUACAGU mmu-let-7g UGAGGUAGUAGUUUGUACAGU

hsa-let-7i UGAGGUAGUAGUUUGUGCUGU mmu-let-7i UGAGGUAGUAGUUUGUGCUGU

hsa-miR-1 UGGAAUGUAAAGAAGUAUGUA mmu-miR-1 UGGAAUGUAAAGAAGUAUGUA

hsa-miR-100 AACCCGUAGAUCCGAACUUGUG mmu-miR-100 AACCCGUAGAUCCGAACUUGUG

hsa-miR-101 UACAGUACUGUGAUAACUGAAG mmu-miR-101a UACAGUACUGUGAUAACUGAAG

hsa-miR-103 AGCAGCAUUGUACAGGGCUAUGA mmu-miR-103 AGCAGCAUUGUACAGGGCUAUGA

hsa-miR-106b UAAAGUGCUGACAGUGCAGAU mmu-miR-106b UAAAGUGCUGACAGUGCAGAU

hsa-miR-107 AGCAGCAUUGUACAGGGCUAUCA mmu-miR-107 AGCAGCAUUGUACAGGGCUAUCA

hsa-miR-10a UACCCUGUAGAUCCGAAUUUGUG mmu-miR-10a UACCCUGUAGAUCCGAAUUUGUG

hsa-miR-122a UGGAGUGUGACAAUGGUGUUUGU mmu-miR-122a UGGAGUGUGACAAUGGUGUUUGU

hsa-miR-125a UCCCUGAGACCCUUUAACCUGUG mmu-miR-125a UCCCUGAGACCCUUUAACCUGUG

hsa-miR-125b UCCCUGAGACCCUAACUUGUGA mmu-miR-125b UCCCUGAGACCCUAACUUGUGA

hsa-miR-126 UCGUACCGUGAGUAAUAAUGC mmu-miR-126-3p UCGUACCGUGAGUAAUAAUGC

hsa-miR-126* CAUUAUUACUUUUGGUACGCG mmu-miR-126-5p CAUUAUUACUUUUGGUACGCG

hsa-miR-127 UCGGAUCCGUCUGAGCUUGGCU mmu-miR-127 UCGGAUCCGUCUGAGCUUGGCU

hsa-miR-128a UCACAGUGAACCGGUCUCUUUU mmu-miR-128a UCACAGUGAACCGGUCUCUUUU

hsa-miR-128b UCACAGUGAACCGGUCUCUUUC mmu-miR-128b UCACAGUGAACCGGUCUCUUUC

hsa-miR-129 CUUUUUGCGGUCUGGGCUUGCU mmu-miR-129-5p CUUUUUGCGGUCUGGGCUUGCU

hsa-miR-130a CAGUGCAAUGUUAAAAGGGCAU mmu-miR-130a CAGUGCAAUGUUAAAAGGGCAU

hsa-miR-130b CAGUGCAAUGAUGAAAGGGCAU mmu-miR-130b CAGUGCAAUGAUGAAAGGGCAU

hsa-miR-132 UAACAGUCUACAGCCAUGGUCG mmu-miR-132 UAACAGUCUACAGCCAUGGUCG

hsa-miR-133a UUGGUCCCCUUCAACCAGCUGU mmu-miR-133a UUGGUCCCCUUCAACCAGCUGU

hsa-miR-133b UUGGUCCCCUUCAACCAGCUA mmu-miR-133b UUGGUCCCCUUCAACCAGCUA

hsa-miR-134 UGUGACUGGUUGACCAGAGGGG mmu-miR-134 UGUGACUGGUUGACCAGAGGGG

hsa-miR-135a UAUGGCUUUUUAUUCCUAUGUGA mmu-miR-135a UAUGGCUUUUUAUUCCUAUGUGA

hsa-miR-135b UAUGGCUUUUCAUUCCUAUGUG mmu-miR-135b UAUGGCUUUUCAUUCCUAUGUG

hsa-miR-136 ACUCCAUUUGUUUUGAUGAUGGA mmu-miR-136 ACUCCAUUUGUUUUGAUGAUGGA

hsa-miR-138 AGCUGGUGUUGUGAAUC mmu-miR-138 AGCUGGUGUUGUGAAUC

hsa-miR-139 UCUACAGUGCACGUGUCU mmu-miR-139 UCUACAGUGCACGUGUCU

hsa-miR-141 UAACACUGUCUGGUAAAGAUGG mmu-miR-141 UAACACUGUCUGGUAAAGAUGG

hsa-miR-142-3p UGUAGUGUUUCCUACUUUAUGGA mmu-miR-142-3p UGUAGUGUUUCCUACUUUAUGG

hsa-miR-142-5p CAUAAAGUAGAAAGCACUAC mmu-miR-142-5p CAUAAAGUAGAAAGCACUAC

hsa-miR-143 UGAGAUGAAGCACUGUAGCUCA mmu-miR-143 UGAGAUGAAGCACUGUAGCUCA

hsa-miR-144 UACAGUAUAGAUGAUGUACUAG mmu-miR-144 UACAGUAUAGAUGAUGUACUAG

hsa-miR-145 GUCCAGUUUUCCCAGGAAUCCCUU mmu-miR-145 GUCCAGUUUUCCCAGGAAUCCCUU

hsa-miR-146a UGAGAACUGAAUUCCAUGGGUU mmu-miR-146 UGAGAACUGAAUUCCAUGGGUU

hsa-miR-148a UCAGUGCACUACAGAACUUUGU mmu-miR-148a UCAGUGCACUACAGAACUUUGU

hsa-miR-148b UCAGUGCAUCACAGAACUUUGU mmu-miR-148b UCAGUGCAUCACAGAACUUUGU

hsa-miR-149 UCUGGCUCCGUGUCUUCACUCC mmu-miR-149 UCUGGCUCCGUGUCUUCACUCC

hsa-miR-150 UCUCCCAACCCUUGUACCAGUG mmu-miR-150 UCUCCCAACCCUUGUACCAGUG

hsa-miR-152 UCAGUGCAUGACAGAACUUGGG mmu-miR-152 UCAGUGCAUGACAGAACUUGGG

hsa-miR-153 UUGCAUAGUCACAAAAGUGAUC mmu-miR-153 UUGCAUAGUCACAAAAGUGAUC

hsa-miR-154 UAGGUUAUCCGUGUUGCCUUCG mmu-miR-154 UAGGUUAUCCGUGUUGCCUUCG

hsa-miR-155 UUAAUGCUAAUCGUGAUAGGGG mmu-miR-155 UUAAUGCUAAUUGUGAUAGGGG

hsa-miR-15a UAGCAGCACAUAAUGGUUUGUG mmu-miR-15a UAGCAGCACAUAAUGGUUUGUG

hsa-miR-15b UAGCAGCACAUCAUGGUUUACA mmu-miR-15b UAGCAGCACAUCAUGGUUUACA

hsa-miR-16 UAGCAGCACGUAAAUAUUGGCG mmu-miR-16 UAGCAGCACGUAAAUAUUGGCG

hsa-miR-17-3p ACUGCAGUGAAGGCACUUGUA mmu-miR-17-3p ACUGCAGUGAGGGCACUUGUA

hsa-miR-17-5p CAAAGUGCUUACAGUGCAGGUAGU mmu-miR-17-5p CAAAGUGCUUACAGUGCAGGUAGU

hsa-miR-181a AACAUUCAACGCUGUCGGUGAGU mmu-miR-181a AACAUUCAACGCUGUCGGUGAGU

hsa-miR-181b AACAUUCAUUGCUGUCGGUGGG mmu-miR-181b AACAUUCAUUGCUGUCGGUGGG

hsa-miR-181c AACAUUCAACCUGUCGGUGAGU mmu-miR-181c AACAUUCAACCUGUCGGUGAGU

hsa-miR-182 UUUGGCAAUGGUAGAACUCACA mmu-miR-182 UUUGGCAAUGGUAGAACUCACA

hsa-miR-183 UAUGGCACUGGUAGAAUUCACUG mmu-miR-183 UAUGGCACUGGUAGAAUUCACUG

hsa-miR-184 UGGACGGAGAACUGAUAAGGGU mmu-miR-184 UGGACGGAGAACUGAUAAGGGU

hsa-miR-185 UGGAGAGAAAGGCAGUUC mmu-miR-185 UGGAGAGAAAGGCAGUUC

hsa-miR-186 CAAAGAAUUCUCCUUUUGGGCUU mmu-miR-186 CAAAGAAUUCUCCUUUUGGGCUU

hsa-miR-187 UCGUGUCUUGUGUUGCAGCCGG mmu-miR-187 UCGUGUCUUGUGUUGCAGCCGG

hsa-miR-188 CAUCCCUUGCAUGGUGGAGGGU mmu-miR-188 CAUCCCUUGCAUGGUGGAGGGU

hsa-miR-189 GUGCCUACUGAGCUGAUAUCAGU mmu-miR-189 GUGCCUACUGAGCUGAUAUCAGU

hsa-miR-190 UGAUAUGUUUGAUAUAUUAGGU mmu-miR-190 UGAUAUGUUUGAUAUAUUAGGU

hsa-miR-191 CAACGGAAUCCCAAAAGCAGCU mmu-miR-191 CAACGGAAUCCCAAAAGCAGCU

hsa-miR-192 CUGACCUAUGAAUUGACAGCC mmu-miR-192 CUGACCUAUGAAUUGACAGCC

hsa-miR-193a AACUGGCCUACAAAGUCCCAG mmu-miR-193 AACUGGCCUACAAAGUCCCAG

hsa-miR-194 UGUAACAGCAACUCCAUGUGGA mmu-miR-194 UGUAACAGCAACUCCAUGUGGA

hsa-miR-195 UAGCAGCACAGAAAUAUUGGC mmu-miR-195 UAGCAGCACAGAAAUAUUGGC

hsa-miR-196a UAGGUAGUUUCAUGUUGUUGG mmu-miR-196a UAGGUAGUUUCAUGUUGUUGG

hsa-miR-196b UAGGUAGUUUCCUGUUGUUGG mmu-miR-196b UAGGUAGUUUCCUGUUGUUGG

hsa-miR-199a CCCAGUGUUCAGACUACCUGUUC mmu-miR-199a CCCAGUGUUCAGACUACCUGUUC

hsa-miR-199a* UACAGUAGUCUGCACAUUGGUU mmu-miR-199a* UACAGUAGUCUGCACAUUGGUU

hsa-miR-199b CCCAGUGUUUAGACUAUCUGUUC mmu-miR-199b CCCAGUGUUUAGACUACCUGUUC

hsa-miR-19a UGUGCAAAUCUAUGCAAAACUGA mmu-miR-19a UGUGCAAAUCUAUGCAAAACUGA

hsa-miR-19b UGUGCAAAUCCAUGCAAAACUGA mmu-miR-19b UGUGCAAAUCCAUGCAAAACUGA

hsa-miR-200a UAACACUGUCUGGUAACGAUGU mmu-miR-200a UAACACUGUCUGGUAACGAUGU

hsa-miR-200b UAAUACUGCCUGGUAAUGAUGAC mmu-miR-200b UAAUACUGCCUGGUAAUGAUGAC

hsa-miR-200c UAAUACUGCCGGGUAAUGAUGG mmu-miR-200c UAAUACUGCCGGGUAAUGAUGG

hsa-miR-202 AGAGGUAUAGGGCAUGGGAAAA mmu-miR-202 AGAGGUAUAGCGCAUGGGAAGA

hsa-miR-204 UUCCCUUUGUCAUCCUAUGCCUG mmu-miR-204 UUCCCUUUGUCAUCCUAUGCCUG

hsa-miR-205 UCCUUCAUUCCACCGGAGUCUG mmu-miR-205 UCCUUCAUUCCACCGGAGUCUG

hsa-miR-206 UGGAAUGUAAGGAAGUGUGUGG mmu-miR-206 UGGAAUGUAAGGAAGUGUGUGG

hsa-miR-208 AUAAGACGAGCAAAAAGCUUGU mmu-miR-208 AUAAGACGAGCAAAAAGCUUGU

hsa-miR-20a UAAAGUGCUUAUAGUGCAGGUAG mmu-miR-20 UAAAGUGCUUAUAGUGCAGGUAG

hsa-miR-21 UAGCUUAUCAGACUGAUGUUGA mmu-miR-21 UAGCUUAUCAGACUGAUGUUGA

hsa-miR-210 CUGUGCGUGUGACAGCGGCUGA mmu-miR-210 CUGUGCGUGUGACAGCGGCUGA

hsa-miR-211 UUCCCUUUGUCAUCCUUCGCCU mmu-miR-211 UUCCCUUUGUCAUCCUUUGCCU

hsa-miR-212 UAACAGUCUCCAGUCACGGCC mmu-miR-212 UAACAGUCUCCAGUCACGGCC

hsa-miR-213 ACCAUCGACCGUUGAUUGUACC mmu-miR-213 ACCAUCGACCGUUGAUUGUACC

hsa-miR-214 ACAGCAGGCACAGACAGGCAG mmu-miR-214 ACAGCAGGCACAGACAGGCAG

hsa-miR-215 AUGACCUAUGAAUUGACAGAC mmu-miR-215 AUGACCUAUGAUUUGACAGAC

hsa-miR-216 UAAUCUCAGCUGGCAACUGUG mmu-miR-216 UAAUCUCAGCUGGCAACUGUG

hsa-miR-217 UACUGCAUCAGGAACUGAUUGGAU mmu-miR-217 UACUGCAUCAGGAACUGACUGGAU

hsa-miR-218 UUGUGCUUGAUCUAACCAUGU mmu-miR-218 UUGUGCUUGAUCUAACCAUGU

hsa-miR-219 UGAUUGUCCAAACGCAAUUCU mmu-miR-219 UGAUUGUCCAAACGCAAUUCU

hsa-miR-22 AAGCUGCCAGUUGAAGAACUGU mmu-miR-22 AAGCUGCCAGUUGAAGAACUGU

hsa-miR-221 AGCUACAUUGUCUGCUGGGUUUC mmu-miR-221 AGCUACAUUGUCUGCUGGGUUUC

hsa-miR-222 AGCUACAUCUGGCUACUGGGUCUC mmu-miR-222 AGCUACAUCUGGCUACUGGGUCUC

hsa-miR-223 UGUCAGUUUGUCAAAUACCCC mmu-miR-223 UGUCAGUUUGUCAAAUACCCC

hsa-miR-23a AUCACAUUGCCAGGGAUUUCC mmu-miR-23a AUCACAUUGCCAGGGAUUUCC

hsa-miR-23b AUCACAUUGCCAGGGAUUACC mmu-miR-23b AUCACAUUGCCAGGGAUUACC

hsa-miR-24 UGGCUCAGUUCAGCAGGAACAG mmu-miR-24 UGGCUCAGUUCAGCAGGAACAG

hsa-miR-25 CAUUGCACUUGUCUCGGUCUGA mmu-miR-25 CAUUGCACUUGUCUCGGUCUGA

hsa-miR-26a UUCAAGUAAUCCAGGAUAGGC mmu-miR-26a UUCAAGUAAUCCAGGAUAGGC

hsa-miR-26b UUCAAGUAAUUCAGGAUAGGUU mmu-miR-26b UUCAAGUAAUUCAGGAUAGGUU

hsa-miR-27a UUCACAGUGGCUAAGUUCCGC mmu-miR-27a UUCACAGUGGCUAAGUUCCGC

hsa-miR-27b UUCACAGUGGCUAAGUUCUGC mmu-miR-27b UUCACAGUGGCUAAGUUCUGC

hsa-miR-28 AAGGAGCUCACAGUCUAUUGAG mmu-miR-28 AAGGAGCUCACAGUCUAUUGAG

hsa-miR-296 AGGGCCCCCCCUCAAUCCUGU mmu-miR-296 AGGGCCCCCCCUCAAUCCUGU

hsa-miR-299-5p UGGUUUACCGUCCCACAUACAU mmu-miR-299 UGGUUUACCGUCCCACAUACAU

hsa-miR-29a UAGCACCAUCUGAAAUCGGUU mmu-miR-29a UAGCACCAUCUGAAAUCGGUU

hsa-miR-29b UAGCACCAUUUGAAAUCAGUGUU mmu-miR-29b UAGCACCAUUUGAAAUCAGUGUU

hsa-miR-29c UAGCACCAUUUGAAAUCGGU mmu-miR-29c UAGCACCAUUUGAAAUCGGU

hsa-miR-301 CAGUGCAAUAGUAUUGUCAAAGC mmu-miR-301 CAGUGCAAUAGUAUUGUCAAAGC

hsa-miR-302a UAAGUGCUUCCAUGUUUUGGUGA mmu-miR-302 UAAGUGCUUCCAUGUUUUGGUGA

hsa-miR-30a-3p CUUUCAGUCGGAUGUUUGCAGC mmu-miR-30a-3p CUUUCAGUCGGAUGUUUGCAGC

hsa-miR-30a-5p UGUAAACAUCCUCGACUGGAAG mmu-miR-30a-5p UGUAAACAUCCUCGACUGGAAG

hsa-miR-30b UGUAAACAUCCUACACUCAGCU mmu-miR-30b UGUAAACAUCCUACACUCAGCU

hsa-miR-30c UGUAAACAUCCUACACUCUCAGC mmu-miR-30c UGUAAACAUCCUACACUCUCAGC

hsa-miR-30d UGUAAACAUCCCCGACUGGAAG mmu-miR-30d UGUAAACAUCCCCGACUGGAAG

hsa-miR-30e-3p CUUUCAGUCGGAUGUUUACAGC mmu-miR-30e* CUUUCAGUCGGAUGUUUACAG

hsa-miR-30e-5p UGUAAACAUCCUUGACUGGA mmu-miR-30e UGUAAACAUCCUUGACUGGA

hsa-miR-32 UAUUGCACAUUACUAAGUUGC mmu-miR-32 UAUUGCACAUUACUAAGUUGC

hsa-miR-320 AAAAGCUGGGUUGAGAGGGCGAA mmu-miR-320 AAAAGCUGGGUUGAGAGGGCGAA

hsa-miR-323 GCACAUUACACGGUCGACCUCU mmu-miR-323 GCACAUUACACGGUCGACCUCU

hsa-miR-324-3p CCACUGCCCCAGGUGCUGCUGG mmu-miR-324-3p CCACUGCCCCAGGUGCUGCUGG

hsa-miR-324-5p CGCAUCCCCUAGGGCAUUGGUGU mmu-miR-324-5p CGCAUCCCCUAGGGCAUUGGUG

hsa-miR-325 CCUAGUAGGUGUCCAGUAAGUGU mmu-miR-325 CCUAGUAGGUGCUCAGUAAGUGU

hsa-miR-326 CCUCUGGGCCCUUCCUCCAGU mmu-miR-326 CCUCUGGGCCCUUCCUCCAGU

hsa-miR-328 CUGGCCCUCUCUGCCCUUCCGU mmu-miR-328 CUGGCCCUCUCUGCCCUUCCGU

hsa-miR-329 AACACACCUGGUUAACCUCUUU mmu-miR-329 AACACACCCAGCUAACCUUUUU

hsa-miR-33 GUGCAUUGUAGUUGCAUUG mmu-miR-33 GUGCAUUGUAGUUGCAUUG

hsa-miR-330 GCAAAGCACACGGCCUGCAGAGA mmu-miR-330 GCAAAGCACAGGGCCUGCAGAGA

hsa-miR-331 GCCCCUGGGCCUAUCCUAGAA mmu-miR-331 GCCCCUGGGCCUAUCCUAGAA

hsa-miR-335 UCAAGAGCAAUAACGAAAAAUGU mmu-miR-335 UCAAGAGCAAUAACGAAAAAUGU

hsa-miR-338 UCCAGCAUCAGUGAUUUUGUUGA mmu-miR-338 UCCAGCAUCAGUGAUUUUGUUGA

hsa-miR-339 UCCCUGUCCUCCAGGAGCUCA mmu-miR-339 UCCCUGUCCUCCAGGAGCUCA

hsa-miR-340 UCCGUCUCAGUUACUUUAUAGCC mmu-miR-340 UCCGUCUCAGUUACUUUAUAGCC

hsa-miR-342 UCUCACACAGAAAUCGCACCCGUC mmu-miR-342 UCUCACACAGAAAUCGCACCCGUC

hsa-miR-346 UGUCUGCCCGCAUGCCUGCCUCU mmu-miR-346 UGUCUGCCCGAGUGCCUGCCUCU

hsa-miR-34a UGGCAGUGUCUUAGCUGGUUGUU mmu-miR-34a UGGCAGUGUCUUAGCUGGUUGUU

hsa-miR-34b UAGGCAGUGUCAUUAGCUGAUUG mmu-miR-34b UAGGCAGUGUAAUUAGCUGAUUG

hsa-miR-34c AGGCAGUGUAGUUAGCUGAUUGC mmu-miR-34c AGGCAGUGUAGUUAGCUGAUUGC

hsa-miR-361 UUAUCAGAAUCUCCAGGGGUAC mmu-miR-361 UUAUCAGAAUCUCCAGGGGUAC

hsa-miR-363 AUUGCACGGUAUCCAUCUGUAA mmu-miR-363 AUUGCACGGUAUCCAUCUGUAA

hsa-miR-365 UAAUGCCCCUAAAAAUCCUUAU mmu-miR-365 UAAUGCCCCUAAAAAUCCUUAU

hsa-miR-370 GCCUGCUGGGGUGGAACCUGGUU mmu-miR-370 GCCUGCUGGGGUGGAACCUGGUU

hsa-miR-375 UUUGUUCGUUCGGCUCGCGUGA mmu-miR-375 UUUGUUCGUUCGGCUCGCGUGA

hsa-miR-376b AUCAUAGAGGAAAAUCCAUGUU mmu-miR-376b AUCAUAGAGGAACAUCCACUUU

hsa-miR-377 AUCACACAAAGGCAACUUUUGU mmu-miR-377 AUCACACAAAGGCAACUUUUGU

hsa-miR-378 CUCCUGACUCCAGGUCCUGUGU mmu-miR-378 CUCCUGACUCCAGGUCCUGUGU

hsa-miR-379 UGGUAGACUAUGGAACGUAGG mmu-miR-379 UGGUAGACUAUGGAACGUAGG

hsa-miR-380-3p UAUGUAAUAUGGUCCACAUCUU mmu-miR-380-3p UAUGUAGUAUGGUCCACAUCUU

hsa-miR-380-5p UGGUUGACCAUAGAACAUGCGC mmu-miR-380-5p UGGUUGACCAUAGAACAUGCGC

hsa-miR-381 UAUACAAGGGCAAGCUCUCUGU mmu-miR-381 UAUACAAGGGCAAGCUCUCUGU

hsa-miR-382 GAAGUUGUUCGUGGUGGAUUCG mmu-miR-382 GAAGUUGUUCGUGGUGGAUUCG

hsa-miR-383 AGAUCAGAAGGUGAUUGUGGCU mmu-miR-383 AGAUCAGAAGGUGACUGUGGCU

hsa-miR-384 AUUCCUAGAAAUUGUUCAUA mmu-miR-384 AUUCCUAGAAAUUGUUCACA

hsa-miR-410 AAUAUAACACAGAUGGCCUGUU mmu-miR-410 AAUAUAACACAGAUGGCCUGUU

hsa-miR-412 ACUUCACCUGGUCCACUAGCCGU mmu-miR-412 ACUUCACCUGGUCCACUAGCCGU

hsa-miR-424 CAGCAGCAAUUCAUGUUUUGAA mmu-miR-424 CAGCAGCAAUUCAUGUUUUGGA

hsa-miR-425 AUCGGGAAUGUCGUGUCCGCC mmu-miR-425 AUCGGGAAUGUCGUGUCCGCC

hsa-miR-429 UAAUACUGUCUGGUAAAACCGU mmu-miR-429 UAAUACUGUCUGGUAAUGCCGU

hsa-miR-431 UGUCUUGCAGGCCGUCAUGCAGG mmu-miR-431 UGUCUUGCAGGCCGUCAUGCAGG

hsa-miR-433 AUCAUGAUGGGCUCCUCGGUGU mmu-miR-433-3p AUCAUGAUGGGCUCCUCGGUGU

hsa-miR-448 UUGCAUAUGUAGGAUGUCCCAU mmu-miR-448 UUGCAUAUGUAGGAUGUCCCAU

hsa-miR-449 UGGCAGUGUAUUGUUAGCUGGU mmu-miR-449 UGGCAGUGUAUUGUUAGCUGGU

hsa-miR-450 UUUUUGCGAUGUGUUCCUAAUA mmu-miR-450 UUUUUGCGAUGUGUUCCUAAUA

hsa-miR-451 AAACCGUUACCAUUACUGAGUUU mmu-miR-451 AAACCGUUACCAUUACUGAGUUU

hsa-miR-452 UGUUUGCAGAGGAAACUGAGAC mmu-miR-452 UGUUUGCAGAGGAAACUGAGAC

hsa-miR-7 UGGAAGACUAGUGAUUUUGUUG mmu-miR-7 UGGAAGACUAGUGAUUUUGUUG

hsa-miR-9 UCUUUGGUUAUCUAGCUGUAUGA mmu-miR-9 UCUUUGGUUAUCUAGCUGUAUGA

hsa-miR-9* UAAAGCUAGAUAACCGAAAGU mmu-miR-9* UAAAGCUAGAUAACCGAAAGU

hsa-miR-92 UAUUGCACUUGUCCCGGCCUG mmu-miR-92 UAUUGCACUUGUCCCGGCCUG

hsa-miR-96 UUUGGCACUAGCACAUUUUUGCU mmu-miR-96 UUUGGCACUAGCACAUUUUUGCU

hsa-miR-98 UGAGGUAGUAAGUUGUAUUGUU mmu-miR-98 UGAGGUAGUAAGUUGUAUUGUU

hsa-miR-99b CACCCGUAGAACCGACCUUGCG mmu-miR-99b CACCCGUAGAACCGACCUUGCG
